# Monodentate Phosphinoamine Nickel Complex Supported on a Metal–Organic Framework for High‐Performance Ethylene Dimerization

**DOI:** 10.1002/advs.202309540

**Published:** 2024-06-04

**Authors:** Wenmiao Chen, Palani Elumalai, Hind Mamlouk, Ángel Rentería‐Gómez, Yempally Veeranna, Sharan Shetty, Dharmesh Kumar, Ma'moun Al‐Rawashdeh, Somil S. Gupta, Osvaldo Gutierrez, Hong‐Cai Zhou, Sherzod T. Madrahimov

**Affiliations:** ^1^ Division of Arts and Sciences Texas A&M University at Qatar Education City, P.O. Box Doha 23874 Qatar; ^2^ Department of Chemistry Texas A&M University College Station Texas 77843‐3255 USA; ^3^ Shell India Markets Pvt Ltd. Bengaluru Karnataka 562149 India; ^4^ Qatar Shell Research and Technology Center Qatar Science and Technology Park Tech 1 Building Doha Qatar; ^5^ Department of Chemical Engineering Texas A&M University at Qatar Education City, P.O. Box Doha 23874 Qatar

**Keywords:** catalysis, ethylene dimerization, mechanistic studies, metal–organic frameworks

## Abstract

Ethylene dimerization is an efficient industrial chemical process to produce 1‐butene, with demanding selectivity and activity requirements on new catalytic systems. Herein, a series of monodentate phosphinoamine‐nickel complexes immobilized on **UiO‐66** are described for ethylene dimerization. These catalysts display extensive molecular tunability of the ligand similar to organometallic catalysis, while maintaining the high stability attributed to the metal–organic framework (MOF) scaffold. The highly flexible postsynthetic modification method enables this study to prepare MOFs functionalized with five different substituted phosphines and 3 N‐containing ligands and identify the optimal catalyst **UiO‐66‐L5‐NiCl_2_** with isopropyl substituted nickel mono‐phosphinoamine complex. This catalyst shows a remarkable activity and selectivity with a TOF of 29 000 (mol_ethyl_/mol_Ni_/h) and 99% selectivity for 1‐butene under ethylene pressure of 15 bar. The catalyst is also applicable for continuous production in the packed column micro‐reactor with a TON of 72 000 (mol_ethyl_/mol_Ni_). The mechanistic insight for the ethylene oligomerization has been examined by density functional theory (DFT) calculations. The calculated energy profiles for homogeneous complexes and truncated MOF models reveal varying rate‐determining step as β‐hydrogen elimination and migratory insertion, respectively. The activation barrier of **UiO‐66‐L5‐NiCl_2_** is lower than other systems, possibly due to the restriction effect caused by clusters and ligands. A comprehensive analysis of the structural parameters of catalysts shows that the cone angle as steric descriptor and butene desorption energy as thermodynamic descriptor can be applied to estimate the reactivity turnover frequency (TOF) with the optimum for **UiO‐66‐L5‐NiCl_2_**. This work represents the systematic optimization of ligand effect through combination of experimental and theoretical data and presents a proof‐of‐concept for ethylene dimerization catalyst through simple heterogenization of organometallic catalyst on MOF.

## Graphical abstract

A system ligand tunning is performed on monodentate phosphinoamine‐nickel complexes tethered **UiO‐66** for ethylene dimerization. The optimized **UiO‐66‐L5‐NiCl_2_** (L5 = (diisopropylphosphino)phosphino amine) exhibits a TOF of 29 000 (mol_ethyl_/mol_Ni_/h) and 99% selectivity for 1‐butene under ethylene pressure of 15 bar. A comprehensive analysis of the structural parameters of catalysts shows that the cone angle as steric descriptor can be applied to estimate the reactivity.

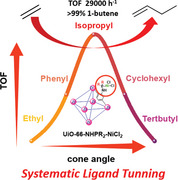

## 1 Introduction

Olefin oligomerization and polymerization reactions are one class of reactions carried out in high volume in industry that are dominated by homogeneous catalysts.^[^ ^1^ ^]^ One key factor prohibiting the application of heterogeneous catalysts is the low activity, commonly denoted as turnover frequency (TOF), in terms of the number of ethylene molecules consumed per catalytically active site per unit time. To enhance the activity and selectivity of the catalytic systems, rational control of the coordination environment of catalysts immobilized on solid supports is necessary.^[^ ^2^ ^]^ The target catalyst is expected to possess precise molecular structure as well as flexible scaffold suitable for functionalization.^[^ ^3^ ^]^

Metal–organic frameworks (MOFs), due to their structural orderliness, high tunability, and synthetic versatility, have found ever‐increasing utility in olefin oligomerization and polymerization reactions.^[^ ^4^ ^]^ The novel post‐synthetic modification (PSM) methods, which are general functionalization strategy in MOFs, COFs, and polymers,^[^ ^3^, ^5^ ^]^ enable researchers to systematically study the coordination environment of ancillary ligands, optimizing the electronic and steric properties of the immobilized catalysts while maintaining the integrity of the framework.^[^ ^6^ ^]^ This is often achieved via substitution of unfunctionalized ligands on the MOF surface with functionalized ligands through ligand exchange.^[^ ^7^ ^]^ Notably, these immobilized systems also enable formation of complexes that are challenging to access in homogeneous systems such as under‐ligated, heteroleptic or *trans*‐coordinated complexes, leading to higher activity or complementary selectivity to those observed for homogeneous systems.^[^ ^8^ ^]^

Bis(diaryl/dialkylphosphino)amine‐type (DPPA) ligands, are classical ancillary ligands in homogeneous dimerization catalysts design.^[^ ^9^, ^10^ ^]^ In examples by Breuil et al, symmetrical bisphosphine nickel complexes, showed relatively high TOF and high C_4_ selectivity (>90%) for ethylene dimerization reactions while varying the bite angles and electronic properties.^[^ ^11^ ^]^ Moreover, researchers found that less coordinated (ex. mono‐phosphine, mono‐imido) metal complexes show enhanced activity due to the presence of an extra coordination site.^[^ ^12^ ^]^ As such, immobilization of these mono‐phosphine complexes on MOFs help generate efficient and portable catalysts. First, it enables us to optimize the catalyst through extensive modification of the ligand structure through facile PSM. Second, immobilization on the MOF stabilizes the molecular underligated catalyst through site isolation effect.^[^ ^13^ ^]^ Overall, the MOF immobilized catalysts aim to achieve activity and selectivity approaching the industrial homogeneous catalysts and superior to the previously reported heterogeneous catalysts.

**Scheme 1:**
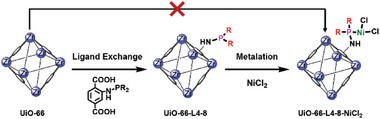
Schematic illustration of the synthesis of monodentate‐phosphinoamine Ni catalysts.

Herein, we report a series of active and selective MOF‐immobilized monodentate‐phosphinoamine Ni complex catalysts for ethylene dimerization reaction under mild conditions. The immobilized catalysts were prepared through the well‐established PSM of zirconium (IV) based MOF **UiOs‐66**, via “ligand exchange” method followed by metalation with Ni.^[^ ^14^ ^]^ The postsynthetic strategy guarantees the formation and maintanence of monodentate‐phosphine Ni structure, preventing the possible dimerization over direct synthesis (**Scheme** **1**). This versatile approach allowed us to install a series of **NHPR_2_** (NHP stands for mono(phosphino)amine R = ‐Et ‐iPr, ‐tBu, ‐Cy and –Ph, **L4‐L8**) ligands with varying substituents to identify the most active catalyst for the ethylene dimerization reaction. As a result, **UiO‐66‐NHPiPr_2_** (**UiO‐66‐L5**) was identified as the optimal catalyst with a TOF up to 29 000 h^−1^ and selectivity for 1‐butene ≈99%. Moreover, quantum mechanical calculations reveal that the high TOF of **NHP*i*Pr_2_**‐supported MOF originates from the differences in the reaction barrier for the migratory insertion as the rate‐determining step, which followed a pseudo‐volcanic relationship in regard of steric effect and butene desorption energy. Finally, to further demonstrate the utility of the **UiO‐66‐NHPiPr_2_** catalysts for recycling and continuous production, a programmed system conducting the ethylene dimerization in the column microreactor was demonstrated.^[^ ^15^ ^]^ The developed system based on MOF catalysts maintained the TOF of 6000 h^−1^ over 12 h.

## 2 Results and Discussion

**Figure 1:**
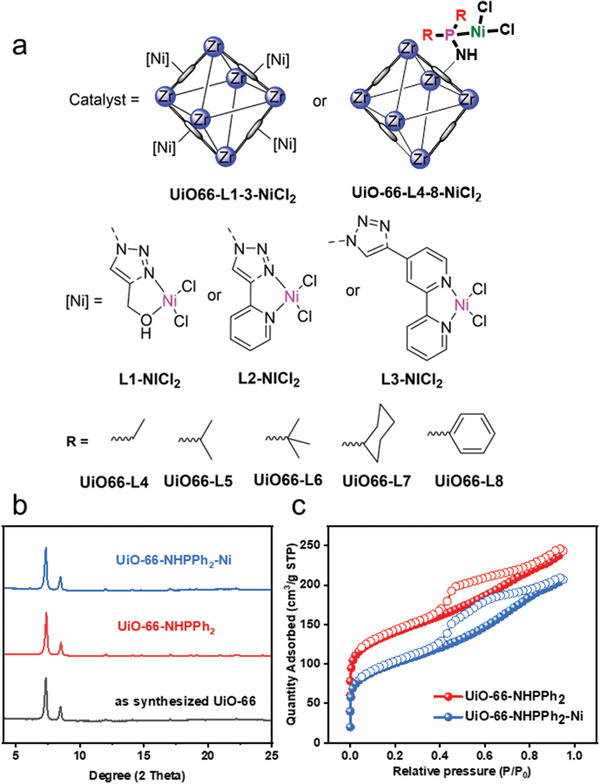
a) Preparation of the UiO‐66‐L1‐L8‐NiCl_2_ through post‐synthetic metalation. b) PXRD of as synthesized UiO‐66, UiO‐66‐NHPh_2_(L8) and UiO‐66‐NHPh_2_(L8)‐Ni c) N_2_ adsorption isotherm of UiO‐66‐NHPh_2_(L8) and UiO‐66‐NHPh_2_(L8)‐Ni.

The **UiO‐66** nanoparticles were prepared in gram scale following previously reported procedure (Section S2.1, Supporting Information).^[^ ^16^ ^]^ The size of nanosized **UiO‐66** was ≈20 nm as observed through SEM image (Figure S1, Supporting Information). The mono‐phosphinoamine ligands were synthesized by a classical aminolysis reaction between amino terephthalic acid (BDC‐NH_2_) and chlorodialkyl‐ or diaryl‐ phosphine in the presence of triethylamine (Section S2.2, Supporting Information). The afforded single‐crystal of the as‐synthesized ligand **BDC‐NHPiPr_2_** and **BDC‐NHPPh_2_**, together with other methods of analysis have confirmed the mono‐phosphinoamine structure(Table S1, Supporting Information). The as‐synthesized ligands were introduced to the MOF through a simple solvent‐assisted ligand exchange procedure (Experimental details in Section S2.3, Supporting Information). Then the ligand exchanged and activated particles were metalated by Ni under inert atmosphere (Experimental details, Section S2.4, Supporting Information). Powder X‐ray diffraction (PXRD) before and after functionalization are almost identical to the as synthesized **UiO‐66**, revealing retention of the framework scaffold (**Figure** **1**b). It is noteworthy that, compared with traditional **UiO‐66**, the nanosized **UiO‐66** we synthesized (20 nm) has lower surface area and higher mesoporosity, which is ascribed to the highly defective structure due to the adopted synthetic procedure.^[^ ^17^ ^]^ On the other hand, this defective nanosized MOF enhances the mass transfer and possesses more exposed surface for functionalization and ethylene dimerization. As shown by N_2_ adsorption isotherms, the hierarchical porosity of original **UiO‐66**‐**nano** was maintained and enlarged in **UiO‐66‐NHPPh_2_ (L8)** and **UiO‐66‐NHPPh_2_(L8)‐Ni**, while at the same time the surface area decreased (Figure 1c; Figure S3a, Supporting Information). The surface area of MOF decreases from 727 m^2^ g^−1^ of **UiO‐66**‐**nano** to 502 m^2^ g^−1^ of **UiO‐66‐NHPPh_2_(L8)** and 356 m^2^ g^−1^ of **UiO‐66‐NHPPh_2_(L8)‐Ni**. The PSM and introduction of phosphine complex likely creates defect and partial deformation of the network leading to the increase in mesopores, while blocking of the micropores by functional groups decreases the total surface area.^[^ ^18^ ^]^ This was evidenced by the distribution of pore volume, where the amount of micropores decreased after PSM while the amount of mesopores increased (Figure S3b, Supporting Information). Although the metal complexes seem to anchor on the MOF surface as the calculated sizes of complexes (0.9 nm, Figure 1a) are bigger than the pocket size of **UiO‐66** (0.7 nm, Figure 1a), the defect‐rich and high level of mesoporosity might lead to catalyst penetration into the MOF crystals. As such, phosphine complexes might exist both on the surface and inside the framework.^[^ ^3^ ^]^ Additionally, three MOFs with *N,O*‐type or *N,N*‐type bipyridine bidentate ligands (**L1‐L3**) were also prepared according to our previous publication (Experimental details, Section S2.5, Supporting Information).^[^ ^14^ ^]^ We verified the formation of each of the corresponding phosphine ligands in **UiO‐66(L4)−(L8)** by matching ^31^P NMR spectra obtained from MOF samples digested in D_2_SO_4_/DMSO‐*d6* to the spectra of independently synthesized methyl esters of diacid linkers. (Figures S4–S9, Supporting Information). We quantified the extent of ligand immobilization via ligand exchange in these five, **UiO‐66(L4)‐(L8)**, samples by integrating the singlet ^31^P peak of the phosphinoamine versus an external phosphine standard with a known concentration (Experimental details, Section S2.3, Supporting Information). As such, the molecular weights calculated per one mole of immobilized ligand were as follows: **UiO‐66‐NHPEt_2_(L4)** 2396 g mol^−1^ **UiO‐66‐NHPiPr_2_(L5)** 3236 g mol^−1^, **UiO‐66‐NHPtBu_2_(L6)** 2327 g mol^−1^, and **UiO‐66‐NHPcyclo_2_(L7)** 2654 g mol^−1^ **UiO‐66‐NHPPh_2_(L8)** 2849 g mol^−1^ (Table S2, Supporting Information).

The metalated MOFs were characterized by energy‐dispersive spectroscopy (EDS) mapping (Figure S8, Supporting Information) and inductively coupled plasma‐mass spectroscopy (ICP‐MS) (Table S3, Supporting Information). The EDS elemental mapping data clearly shows the uniform distribution of Ni and P over the MOF surface, confirming the presence of grafted NHP‐Ni complexes with no Ni agglomeration observed. It is noteworthy that the MOF particles are an aggregation of nano‐sized particles ≈20 nm as shown in the scanning electron microscopy (Figure S1, Supporting Information). The nanosized particles greatly improve the mass transfer of the substrate, as well as exposing more surface anchoring sites with a Ni/Zr ratio 0.140 as shown in the ICP‐MS (Table S3, Supporting Information). Also, from the digested NMR, the exchanged ligand NHP to BDC ratio is 0.5. The relatively high ligand exchange ratio and Ni to Zr ratio further supports that phosphine complexes could be present both on surface and inside the pores. Thus, the calculated molecular formula for **UiO‐66‐NHPPh_2_** is Zr_6_O_4_(OH)_4_(BDC)_4_(BDC‐NHPPPh_2_)_2_Ni_0.8_, which means that not all phosphine ligands are coordinated with the metal. The immobilization of the phosphine ligand was further verified by solid‐state NMR (SS‐NMR) and X‐ray photoelectron spectroscopy (XPS). **UiO‐66‐NHPPh_2_** SS‐NMR show singlet phosphine peak at 24.9, matching the free ligand peak at 23.7 ppm (Figure S9, Supporting Information). The states variation of phosphine ligands was probed by X‐ray photoelectron spectroscopy (XPS). The spectra are deconvoluted through the Gaussian peak fitting method and then interpreted based on binding energy. P 2p spectrum of **UiO‐66‐NHPPh_2_** before metalation shows one peak at 129.7 eV, corresponding to P ligand (Figure S10a, Supporting Information). After metalation, P 2p components of **UiO‐66‐NHPPh_2_‐NiCl_2_** are fitted to two peaks placed at 129.7 and 130.3 eV, in which the new peak corresponds to the 2p_1/2_ of P‐Ni species (Figure S10b, Supporting Information).^[^ ^19^ ^]^ Further Ni 2p spectrum exhibit two peaks at 852.9 and 871.7 eV in Ni 2p_3/2_ and Ni 2p_1/2_ region. Two typical shakeup satellite peaks are observed at 858.2and 878.2 eV, respectively (Figure S10c, Supporting Information). These results support the presence of a characteristic Ni^2+^ state coordinated with phosphine.^[^ ^20^ ^]^

The reaction optimization was conducted with varied solvent temperature, activator selection, and loading (Table S4, Supporting Information). In homogeneous Ni‐based catalysts, organometallic activators (AlMe_3_, Et_2_AlCl) are commonly used due to the chemical compatibility.^[^ ^21^ ^]^ Thus, as the heterogenized Ni‐phosphine complex, **UiO‐66‐L8‐Ni** activated by Et_2_AlCl show much higher TOF compared to that activated by MMAO, with an order of magnitude difference (Table S4, Supporting Information, entry 1 and 5). The loading of Et_2_AlCl correlates positively with the TOF, especially when the quantity is increased from 20 to 50 equiv. (Table S4, Supporting Information, entry 1 and 10). Further increasing the amount of Et_2_AlCl to 100 equiv. leads to only a slight improvement in activity (Table S4, Supporting Information, entry 11). Lower temperature is thermodynamically favorable due to the exothermic nature of ethylene dimerization (ΔH = −52.91 kJ mol^−1^). Using **UiO‐66‐L8‐Ni** as catalyst, the selectivity of 1‐butene reaches 97% at 0 °C, higher than the selectivity observed at 25 °C and 50 °C (Table S4, Supporting Information, entry 1, 8 and 9). On the other hand, the TOF reaches a maximum of 22 500 at 25 °C and 15 bar. Similar temperature‐dependent reactivity can be found in other Ni‐MOF‐based systems, which is caused by opposing thermodynamic and kinetic effects.^[^ ^22^ ^]^ The ethylene dimerization under optimized condition are summarized in **Table** **1**. We conducted the dimerization reaction at room temperature, in a 100 mL Parr reactor under the total pressure of 15 bar, using Heptane as solvent and activated by Et_2_AlCl. We have tried two classes of immobilized molecular catalysts, three catalysts with bidentate N,N or N,O catalysts **UiO‐66‐L1‐L3‐NiCl_2_** that were prepared through a post synthesis functionalization “click” reaction as reported earlier,^[^ ^14^ ^]^ and a series of catalysts with monodentate phosphinoamine ligands **UiO‐66‐L4‐8‐NiCl_2_** (Figure 1a). The complexes with the phosphinoamine ligands clearly showed much higher activities than the immobilized N,N or N,O ligands‐based catalysts. This observation is similar to what is observed for homogeneous systems. Among the systems we tried, bis‐isopropyl substituted catalyst **UiO‐66‐L5‐NiCl_2_** showed the highest TOF of 29 000 (mol ethylene)/(mol Ni·h) (Table 1, entry 5). This observed activity is higher than activities for immobilized catalysts reported in literature and is in the same order of magnitude with the best industrial catalysts (Table S5, Supporting Information)^[^ ^21^, ^23^ ^]^ The TOF and selectivity are comparable with reported homogeneous Ni complexes, which illustrate the strength of monodentate‐Ni complexes. More importantly, formation of underligated‐Ni complexes on MOF relies on the postsynthetic modification strategy. Additionally, the tested immobilized catalysts showed exclusive selectivity toward the formation of the terminal 1‐butene product and the product mixture showed almost no traces of either the isomerized 2‐butene or higher oligomerization products such as hexenes or octenes. We attribute the high activity and stability of the MOF catalysts to immobilized nature of these systems that prevent the complexes from agglomeration and deactivation.^[^ ^13^, ^24^ ^]^ The identity of the immobilized complex as the catalytically active species was also corroborated through a series of control experiments. Both **UiO‐66** and **UiO‐66‐NHPPh_2_(L8)** in the presence of Et_2_AlCl showed no reactivity (entries 9, 10). The **UiO‐66** with Ni salt, system lacking the immobilized ligand, showed low TOF and selectivity, demonstrating the importance of auxiliary ligands (Table 1, entry 11).

**Table 1:** Ethylene dimerization reaction catalyzed by MOF‐immobilized nickel complexes.

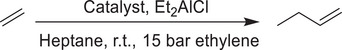			
	MOF catalyst	TOF^a)^, ^b)^ ^)^	1‐butene (%)
1	UiO‐66‐L1‐NiCl_2_	500	97
2	UiO‐66‐L2‐NiCl_2_	2000	95
3	UiO‐66‐L3‐NiCl_2_	900	98
4	UiO‐66‐L4‐NiCl_2_	9500	92
5	UiO‐66‐L5‐NiCl_2_	29 000	99
6	UiO‐66‐L6‐NiCl_2_	9500	90
7	UiO‐66‐L7‐NiCl_2_	18 000	95
8	UiO‐66‐L8‐NiCl_2_	22 500	95
9	UiO‐66	N/A	N/A
10	UiO‐66‐L8	N/A	N/A
11	UiO‐66‐Ni	300	5

^a)^ TOF shown in units of (mol _ethylene_)/(mol _Ni·h_);

^b)^ Calculated from GC‐FID peak integrations of the brominated products against heptane solvent. Condition: The MOF catalysts (6 mg, 2 µmol) were preactivated with 40 µL Et_2_AlCl (20 equiv.) activator solution in 10 mL Heptane for 1 h and used to catalyze the reactions in stainless steel reactor at 15 bar ethylene. Reaction details are provided in S2.6.

One advantage of the heterogeneous catalysts over the homogeneous ones is the ability to operate solventless, under gas‐phase conditions. As such, to probe if the catalyst would display high activity and selectivity similar to solution state reactions, we have tested **UiO‐66‐L5‐NiCl_2_** under gas phase. (Experimental details in Section S2.7 and Table S6, Supporting Information). We have observed the TOF of 10 900 (mol C_2_H_4_)(mol Ni)^−1^ h^−1^ averaged over the total of three runs in a small‐scale batch reactor and similarly to the solution phase reactions, almost exclusive selectivity for 1‐butene. Further recovered catalyst was tested in solution phase and showed TOF of 25 000 (mol C_2_H_4_)(mol Ni)^−1^ h^−1^ (Table S5, Supporting Information, entry 4). Albeit the gas‐phase activity was smaller compared to solution state it was still quite significant indicating that this system can be transferred to a gas‐phase flow reactor for further studies.

**Figure 2:**
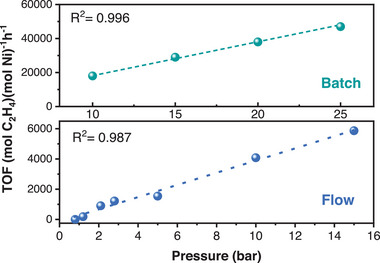
Pressure (bar) versus Reactivity (mol C_2_H_4_)(mol Ni)^−1^ h^−1^ graph of ethylene dimerization for **UiO‐66‐L5‐Ni** in the batch conditions and in the microflow conditions.

Encouraged by the results of gas‐phase batch reactions and to further exploit the advantages of the heterogeneous catalysts, we packed the **UiO‐66‐L5‐Ni** in the microreactor to run the gas phase dimerization reaction under flow condition (Experimental details in Section S2.8, Supporting Information). The catalyst granules for packing into the column were prepared via a grounding and meshing process to afford a particle size that was suitable for application under microflow conditions as described in our previous report (Section S2.6, Supporting Information).^[^ ^15^, ^17^ ^]^ After loading the column the packed catalyst was pretreated with the heptane solution of activator Et_2_AlCl for 30 minutes before passing the ethylene gas through the column at flow rate of 5 mL min^−1^. The reaction pressure was screened in the range from atmospheric pressure to 15 bar and showed a linear increase in TOF with increasing pressure as shown in **Figure** **2**b. A similar linear increase in catalyst activity was also observed for the solution phase batch reactor in the pressure range from 10 to 25 bar Figure 2a. Under both batch and microflow conditions, the selectivity is not affected by pressure (≈99%) within the testing pressure range of: 10–25 bar for batch, and 1–15 bar for the microflow conditions. Within this pressure range, the catalyst productivity is proportional to the pressure. This indicates that the catalyst has not reached the saturation point and activity can increase further with increasing the ethylene pressure demonstrating the great potential for industrial application. Furthermore, the first‐order dependence of activity on gas pressure in both batch reactor and flow microreactor implicates a coordination–insertion pathway for chain growth, confirming the Cossee‐Arlman mechanism.^[^ ^4c^ ^]^ The turnover frequency (TOF) of the flow process was calculated to be 6000 h^−1^ (tested under 15 bar), which gave out a final turnover number (TON) of 72 000 over 12 h based on the stability test (Figure S11, Supporting Information). After the reaction, the packed catalyst was collected and analyzed by PXRD. The peaks remained unchanged indicating the conservation of crystallinity and the broadening of peaks is attributed to the grounding and mechanical pelleting of particles before microflow experiments (Figure S12, Supporting information).

It is noteworthy to mention that these results highlight the strength of our approach to catalyst immobilization that allows us to systematically tune coordination microenvironment of the metal center. To better compare the ligand effect of the catalysts, we separated the candidates into two groups as pyridine type ligands **L1‐3** and phosphine ligands **L4‐8**. Among the *N,N* or *N,O* type ligands (**UiO‐66‐L1‐3**), **L1** is the least donating and **L3** is the bulkiest as reported in the literature, resulting in **L2** as the highest TOF (2000).^[^ ^25^ ^]^ This substituent‐dependent reactivity indicates a fine control of the coordination environment in N‐containing ligands, which is a common strategy for organometallic catalyst design.^[^ ^12^, ^26^ ^]^ Regarding the phosphine ligands **L4‐8**, the immobilized catalyst derived from the isopropyl substituted (phosphino)amine ligand, **L5** proved to possess the highest turnover frequency (29 000). In the traditional catalysts design of (phosphino)amine complexes, the partial charge as well as exposure of active sites influence the activity and selectivity of the catalyst.^[^ ^27^ ^]^ Interestingly, ligand **L5** is neither the most nor the least sterically demanding or electron donating among the ligands tested in this work.

**Scheme 2:**
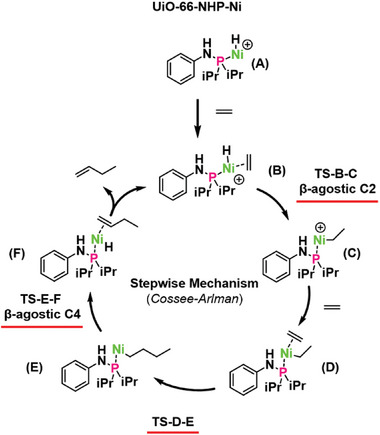
Schematic representation of the reaction pathway following Cossee–Arlman mechanism.

To shed light into the factors controlling reactivity, as shown in **Scheme** **2**, we turn to density functional theory (DFT) calculations using UiO‐66‐NHP‐Ni (**A**) as the nickel model system (See SI for details). In agreement with other known Ni‐based MOF catalysts,^[^ ^4^, ^22^, ^28^ ^]^ the Cossee‐Arlman mechanism has been adapted to describe the ethylene dimerization reaction. (Scheme 2). As shown in Scheme 2, stepwise insertion that proceeds via inital adsorption of ethylene on the activated Ni‐hydride (**A**) to form η−2 bonded intermediate (**B**), followed by the formation of an ethyl linked intermediate (**C**) resulting from migratory insertion transition state (**TS‐B‐C**) that benefits from a β‐agostic‐C2 interaction (vide infra). In turn, a second ethylene molecule is adsorbed to form complex (**D**) followed by alkyl migratory insertion to form the corresponding Ni‐butyl intermediate (**E**). Finally, β‐hydride elimination (via **TS‐E‐F**) will form Ni‐coordinated 1‐butene (**F**) which can then release the product and undergo coordination with another ethylene molecule to restart the catalytic cycle.^[^ ^29^ ^]^

**Figure 3:**
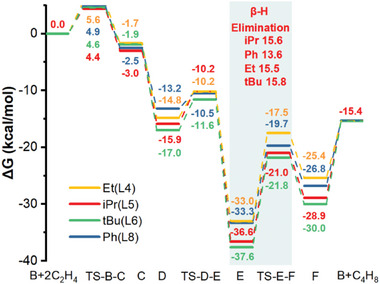
Free energy diagram for the ethylene dimerization on UiO‐66‐NHP‐Ni through Cossee–Arlman reaction pathway at 298K and 1 bar. The intrinsic free energy barriers at RDS measured between the TS and its preceding intermediate are shown. The energies shown were computed by DFT as reported in SI.

To examine the energetic feasibility of this proposed mechanism, in the absence of MOF (vide infra), and evaluate the impact of ligands **L4, L5, L6,** and **L8** on the catalytic cycle (See Figures S15–S18, Supporting Information) we carried out dispersion corrected DFT calculations (See SI for methods and details). For simplicity, we only show and discuss the lowest energy pathway. As shown in **Figure** **3**, the first migratory hydride insertion to ethylene proceeds rapidly via a **TS‐B‐C** (barrier, ≈4–5 kcal mol^−1^) for all ligands. Similarly, the second alkyl migratory insertion to ethylene also proceeds via a low barrier (via **TS‐D‐E**) with lowest Δ*G* ^‡^ of 2.7 kcal mol^−1^ for **L8** (‐Ph) and highest ΔG^‡^ of ≈5.0 kcal mol^−1^ for **L4 – L6**. Remarkably, the β‐hydride elimination step via **TS‐E‐F** exhibits the higher energy barrier with all the ligands (Δ*G* ^‡^ = ≈15.0 kcal mol^−1^), indicating that β‐H elimination is the rate‐determining step (RDS). In addition, we found that isomerization via b‐hydride insertion at the terminal carbon is not thermodynamically favorable (uphill by 9.9 kcal mol^−1^ from **F**, Figure S19, Supporting Information). On the other hand, the calculated barrier for propagation is more favorable (i.e., DG^‡^ = 12.4 kcal mol^−1^) and thermodynamically favored (Figure S20, Supporting Information). Further, given that butyl chains are known to kinetically hinder the olefin approach to the active site in Ni catalysts,^[^ ^28^, ^30^ ^]^ 1‐butene desorption is more likely to occur than hexene formation.

To further explore the role of MOF scaffold beyond the phosphine‐Ni complex, we conducted additional calculations on finite phosphine complexes, with Zr_6_ cluster from UiO‐66 located near the reaction site. Due to the high computational cost, some of the selected linkers were truncated and the spin state was chosen that gives the lowest energy. In general, the calculation of truncated cluster model with *i*Pr ligand reveals the highest energy barrier for migratory insertion for **TS‐D‐E** ΔG^‡^ = 16.2 kcal mol^−1^), indicating migratory insertion as the RDS(Figure S21, Supporting Information). These results agree with the other MOF‐based Ni oligomerization catalysts.^[^ ^28^, ^31^ ^]^ Additional calculations on truncated cluster model with Ph and *t*Bu ligand show similar energy profile and RDS (Figure S22, Supporting Information). Importantly, through comparing the energy barrier at RDS, the *i*Pr (16.2 kcal mol^−1^) exhibits lower activation energy than Ph (22.0 kcal mol^−1^) and *t*Bu (24.7 kcal mol^−1^), matching well with the order of TOF. This clearly indicates the thermodynatic influence on reactivity through ligand optimization.

The variation on RDS and energy profile between truncated cluster model and complex model could be explained by the confinement effect in MOFs, where the cluster restricts the flexibility of the Ni‐intermediate complexes. Additional *distortion energy* analysis^[^ ^32^ ^]^ for **TS‐D‐E** on truncated MOF and complex model with ‐*i*Pr ligand revealed a notable difference in the distortion energy caused by the MOF node (32.4 vs 41.6 kcal mol^−1^, Table S7, Supporting Information). Additionally, the differences in the geometry in **TS‐D‐E** between the two systems indicate the impact of the MOF node. (Figure S23, Supporting Information). The **UiO‐66‐L5‐Ni** exhibits first‐order relation of TOF with respect to ethylene pressure, which is consistent with that the ethylene insertion is RDS and ethylene is the limiting reagent(Figure 2).^[^ ^31b^ ^]^ Moreover, as the chain termination step, β‐H elimination is more favored than ethylene insertion, which explains the high selectivity toward 1‐butene.^[^ ^29^ ^]^ This is also supported by the results from the review by Speiser et al on the higher activity of Ni complexes with phosphine ligands due to facile β‐hydride elimination.^[^ ^33^ ^]^

**Figure 4:**
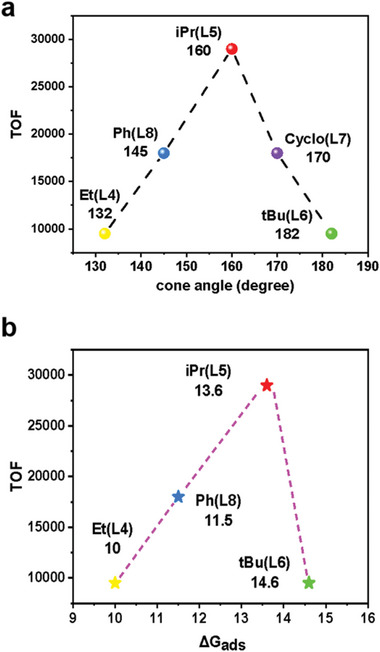
a) Schematic relationship of the TOF versus the cone angle (spheres) and b) butene desorption energy of ligand **L4‐L8**.

Inspired by the regulation effect on the dimerization activities, we tried to interpret the ligand‐dependent reactivity through introducing structural and thermodynamic descriptors. In homogeneous phosphine‐assisted catalytic reactions, cone angle is a common descriptor to quantify the steric effect.^[^ ^34^ ^]^ Cone angle is defined by the degree of cone formed with metal at the vertex and the phosphine substituents at the perimeter of the cone. **Figure** **4**a summarizes the relationship of the cone angle with the TOF of the **UiO‐66(L4)‐(L8)‐Ni** catalysts.^[^ ^35^ ^]^ Notably, a clear volcanic relationship was found between cone angle and TOF, indicating good correlation. This suggests that cone angle could be employed as a descriptor of intrinsic reactivity for ethylene dimerization. Similarly, Passaglia et al. found that, in cationic diphosphine‐Ni complexes, reactivity and selectivity of ethylene oligomerization are dependent on the bite angle of the diphosphine ligand.^[^ ^36^ ^]^ In addition, desorption of butene is endergonic from **F‐B** on energy profiles (Figure S15–S18, Supporting Information). Through plotting the desorption energy of butene (ΔG_des_) with TOF, a quasi‐volcano relationship was found (Figure 4b), indicating thermodynamic effect on the dimerization activity. The moderate adsorption of ethylene on **L5** results in best catalytic performance, reminding us of the well‐known Sabatier principle, that the interaction between catalyst and substrate should be neither too strong nor too weak.^[^ ^37^ ^]^ To the best of our knowledge present work is one of the first attempts to propose a volcanic relationship for the ethylene dimerization, associating activity with the steric and thermodynamic effect of mono‐phosphine Ni immobilized MOF catalysts. More importantly, the energy difference caused by substitution effect of the ligands could be related to the confinement effect of MOF, which is demonstrated in the calculation of *distortion energy* above. It is noteworthy that the volcano‐like plot between TOF and choice of substituents is the result of a group of cumulative effects. Other contributions like the stability of the coordinated metal as well as the mass transfer may be additional reasons affecting the observed activities. For example, ethyl (L4) and tertbutyl (L6) substituted catalysts have comparable activities, while their stabilities maybe different.

## 3 Conclusion

In summary, we developed a series of MOF‐immobilized catalysts for ethylene dimerization and performed optimization of reactivity through substituent screening. The optimized catalyst, **UiO‐66‐L5‐Ni** exhibits a high TOF of 29 000, comparable to some commercial homogeneous catalysts with exclusive selectivity for 1‐butene. This highest activity was attributed to the facilitated migratory insertion, revealed by the DFT calculation of truncated MOF systems. Using cone angle and product desorption energy as descriptors, a pseudo‐volcano relationship was established in regard of activity. Then we applied the catalysts in microreactor under flow condition, which exhibited satisfying TOF and TON. Overall, our results have provided a new scheme for the rational design of MOF‐heterogenized catalyst and offered a quantifiable protocol for estimation of reactivity of monophosphine‐Ni complexes in ethylene dimerization reaction. Additional work on developing MOF catalysts for olefin oligomerizations like trimerization, tetramerization, etc. is underway in our research group.

## Conflict of Interest

The authors declare no conflict of interest.

## Supporting information

Supporting Information

## Data Availability

The data that support the findings of this study are available in the supplementary material of this article.
