# Supplementary material for: Monodentate Phosphinoamine Nickel Complex Supported on a Metal–Organic Framework for High‐Performance Ethylene Dimerization
Source: Adv Sci (Weinh). 2024 Jun 4;11(29):2309540. doi: 10.1002/advs.202309540 (PMC11304313; doi:10.1002/advs.202309540)
Supplement: Supplementary file 1 — Supporting Information [file ADVS-11-2309540-s001.pdf]

ADVANCED SCIENCE
Open Access

Supporting Information

for *Adv. Sci.*, DOI 10.1002/advs.202309540

Monodentate Phosphinoamine Nickel Complex Supported on a Metal–Organic Framework for High-Performance Ethylene Dimerization

Wenmiao Chen, Palani Elumalai, Hind Mamlouk, Ángel Rentería-Gómez, Yempally Veeranna, Sharan Shetty, Dharmesh Kumar, Ma'moun Al-Rawashdeh, Somil S. Gupta, Osvaldo Gutierrez*, Hong-Cai Zhou* and Sherzod T. Madrahimov**

Supporting Information

Aminophosphine-Ni type complex supported on Metal-Organic Framework for high performance ethylene dimerization

Wenmiao Chen,[a, b] † Palani Elumalai,[a] † Hind Mamlouk,[a]† Yempally Veeranna,[a] Ángel Rentería-Gómez, [b] Sharan Shetty,[c] Dharmesh Kumar,[d] Ma'moun Al-Rawashdeh,[e] Somil S. Gupta,[c]* Osvaldo Gutierrez,[b]* Hong-Cai Zhou,[b]* and Sherzod T. Madrahimov[a]*

[a] Department of Science, Texas A&M University at Qatar, Education City, P.O. Box 23874, Doha, Qatar

sherzod.madrahimov@qatar.tamu.edu

[b] Department of Chemistry, Texas A&M University, College Station, Texas 77843-3255, United States

zhou@chem.tamu.edu

[c] Shell India Markets Pvt Ltd., Bengaluru, Karnataka 562149, India

Somil.Gupta@shell.com

[d] Qatar Shell Research and Technology Center, Qatar Science and Technology Park, Tech 1 Building, P. O. Box 3747, Doha, Qatar

[e] Department of Chemical Engineering, Texas A&M University at Qatar, Education City, P.O. Box 23874, Doha, Qatar

KEYWORDS. Metal organic frameworks, Heterogeneous Catalysis, Dimerization, Flow Catalysis

† These authors contributed equally

S1. General Information.

All air- or water-sensitive reactions were carried out under nitrogen atmosphere using oven-dried glassware. All oven-based MOF syntheses were carried out in explosion-proof HERAtherm OMS-100 (Thermo Fisher Scientific, Waltham, MA, USA) ovens that had been pre-heated to a preset temperature. All sonication was carried out with a Fisher Scientific Ultrasonic Cleaner FS60 (Thermo Fisher Scientific, Waltham, MA, USA). All glassware was oven-dried before use.

All flash-column chromatography was carried out using silica gel (MP Silitech 60-200 mesh, MP Biomedicals, LLC, Santa Ana, CA, USA) under a positive pressure of air, unless otherwise noted. Analytical thin layer chromatography (TLC) was performed using glass-backed silica gel 60 F254 plates (Merck EMD-571507, Whitehouse Station, NJ, USA) or aluminum oxide on plastic sheets (J.T. Baker Chemical Co, Phillipsburg, New Jersey). Visualization of TLC results was achieved by observation under UV light (254 nm).

Anhydrous solvents in Sure/Seal™ bottles were purchased from the Aldrich Chemical Company (Milwaukee, WI, USA) and used as received inside a nitrogen-filled Nexus dry box, (Vacuum Atmospheres Company, Hawthorne, CA, USA). All other reagents were purchased from the Aldrich Chemical Company (Milwaukee, WI, USA) and used without further purification, unless otherwise noted. All glassware was oven-dried before use. Deuterated solvents were purchased from Cambridge Isotope Laboratories (Andover, MA, USA) and used without further purification. ICP samples were prepared using DI water additionally purified by Biocel water purification system (EMD Millipore, Billerica, MA, USA).

PXRD was carried out with a Bruker D8-Focus Bragg–Brentano X-ray Powder Diffractometer equipped with a Cu sealed tube (λ=1.54178 Å) at 40 kV and 40 mA. SCXRD was measured on a Bruker Venture CMOS diffractometer equipped with a Cu-Kα sealed-tube X-ray source (λ=1.5406 Å). ICP-MS data were collected with a Perkin Elmer NexION 300D ICP-MS. TGA was conducted on a TGA-50 thermogravimetric analyzer. Field-emission SEM images were collected on the FEI Quanta 600 field-emission SEM at 20 KV. Source: Field emission gun assembly with Schottky emitter source. Beam Current: >100 nA. The high resolution XPS measurements were performed with a Perkin Elmer PHI system. The sample was placed into the XPS chamber with a base pressure of $<1.0 \times 10^{-9}$ Torr. Al Kα (1486.6 eV) X-ray source at a chamber was used to excite photoelectrons. The spectra were recorded by using a 16-channel detector with a hemispherical analyzer.

^{1}H, ^{13}C and ^{31}P NMR spectra were recorded on a Bruker Avance 400 spectrometer and referenced to the residual solvent peak. ^{1}H NMR data are reported as follows: chemical shift (multiplicity (bs = broad singlet, s = singlet, d = doublet, t = triplet, q = quartet, p = pentet and m = multiplet), coupling constant and integration). ^{1}H, ^{13}C and ^{31}P NMR chemical shifts are reported in ppm downfield from tetramethylsilane (TMS, δ scale) using the residual solvent resonances as internal standards.
^{31}P NMR details:
To get quantitative ratio of compound with external standard from the peak ratio. ^{31}P NMR was measured at 400MHz, and 1024 scans, relaxation delay 4 s, an acquisition time of 0.498 sec.

All gas chromatography (GC) analyses at Texas A&M University at Qatar were conducted on a PerkinElmer Inc. - Clarus 500 GC Mass Spectrometer equipped with FID Elite-1 capillary column (30 m × 0.25 m × 0.25 μm film thickness). The GC/MS data were acquired using an Agilent 7890 GC/MSD Gas Chromatograph with 5975 Triple Axis MSD Detector and with a HP-5ms capillary column (30 m × 0.25 mm; film thickness 0.25 μm).

S2. Experimental Section.

S2.1. Synthesis of UiO-66.

UiO-66 was prepared in gram scale and activated according to a previously published procedure by Morris et al.[1] $ZrCl_4$ (200 mg), H_2BDC (100 mg), acetic acid (1.0 mL) and dimethylformamide (DMF 20 mL) were charged in a 40 mL Pyrex vial. The mixture was heated in 120 °C oven for 72 h. After cooling to room temperature, the white crystals were harvested (95 mg, yield: 67%).

S2.2.

Synthesis of mono-(diethylphosphino)amine terephthalate ester ligand (Me-L4)

The $NHPEt_2$ ligand (**L4**) was synthesized according to the literature with slight modification.[2] In a 250 mL flask, Dimethyl amino terephthalate (0.2 g, 1 mmol) and triethyl amine (0.4 g, 4 mmol) were dissolved in 20 mL of dichloromethane. Then the reaction was cooled to 0 ˚C in an Ice bath. To the resulting suspension, Chlorodiethylphosphine (0.31 g, 2.5 mmol) was added dropwise over 15 min. Then the reaction was stirred at room temperature under N_2 protected for 12h. The solution was dried through rotary evaporation and then neutralized with 10% HCl aqueous solution and the product was collected by filtration (0.25 g, yield: 86%).

^{1}H-NMR (400 MHz, DMSO-D_6) δ 9.05 (d, 1H), 8.30 (s, 1H), 8.01 (d, 1H), 3.91 (d, 6H), 1.96 (m, 4H) ,1.18 (m, 6H) . ^{31}P-NMR (400 MHz, $CDCl_3$-d6) δ 40.7 ppm. MW: 297.11

Synthesis of mono-(diethylphosphino) amino terephthalic acid (L4)

The mono-(diethylphosphino) amino terephthalate (0.66 g, 2.46 mmol) was suspended in a 30 mL solution of 1:1:1 mixture of (THF: MeOH: H_2O) in a 100 mL round bottom flask. 0.77 g of KOH (12.5 mmol) pellets were added to the above suspension and stirred at room temperature for 16 h, and then slowly warmed to 40 ˚C, The reaction mixture was stirred at 40 ˚C for additional 4 h. The clear yellow solution obtained was dried through rotary evaporation, and resulting solution was cooled to 0 ˚C using an Ice bath, and 2. 0 M aq HCl solution was added dropwise until yellow color precipitate was observed. The yellow solid was filtered through glass frit and washed with deionized water and finally with cold methanol to obtain light yellow solid (0.69 g, 96 % yield).

^{1}H-NMR (400 MHz, DMSO-D_6) δ 9.21 (d, 1H), 8.39 (s, 1H), 8.10 (d, 1H), 1.87 (m, 4H) ,1.12 (m, 6H) . ^{31}P-NMR (400 MHz, $CDCl_3$-d6) δ 55.08 ppm. MW: 269.16

Synthesis of mono-(diisopropylphosphino)amine terephthalate ester ligand (Me-L5)

The $NHPiPr_2$ ligand (**L5**) was synthesized according to the literature with slight modification.[2] In a 250 mL flask, Dimethyl amino terephthalic acid (1.04 g, 5 mmol), and triethyl amine (2 g, 20 mmol) were dissolved in 50 mL of dichloromethane. Then the reaction was cooled to 0 ˚C in an Ice bath. To the resulting suspension, Chlorodiisopropylphosphine (1.6 g, 10 mmol) was added dropwise over 15 min. Then the reaction was stirred at room temperature under N_2 protected for 12h. The solution was dried through rotary evaporation and then neutralized with 10% HCl aqueous solution and the product was collected by filtration (1.39 g, yield: 85%).

^{1}H-NMR (400 MHz, DMSO-d6) δ 8.24 (d, 1H), 7.92 (d, 2H) 7.28 (d, 1H), 3.90 (d, 6H) 1.87 (m, 2H), 1.09 (m, 12H). ^{31}P-NMR (400 MHz, DMSO-d6) δ 55.8 ppm. MW: 326.22.

Synthesis of mono-(diisopropylphosphino)amine terephthalate acid ligand (L5)

The procedure was revised from synthesis of **L4** with mono-(diisopropylphosphino) amino terephthalate (0.80 g, 2.46 mmol) as reactant.

^{1}H-NMR (400 MHz, DMSO-d6) δ 9.12 (d, 1H), 8.46 (s, 1H), 8.03 (d, 1H), 7.40 (d, 1H) 2.17 (m, 2H) 1.16 (m, 12H). ^{31}P-NMR (400 MHz, DMSO-d6) δ 50.9 ppm. MW: 297.16

Synthesis of mono-(ditertbutylphosphino)amine terephthalate eater ligand (Me-L6)

The $NHPtBu_2$ ligand (**L6**) was synthesized according to the literature with slight modification.[2] In a 250 mL flask, Dimethyl amino terephthalic acid (1.04 g, 5 mmol), and triethyl amine (2 g, 20 mmol) were dissolved in 50 mL of dichloromethane. Then the reaction was cooled to 0 ˚C in an Ice bath. To the resulting suspension, Chloroditertbutylphosphine (1.7 g, 10 mmol) was added dropwise over 15 min. Then the reaction was stirred at room temperature under N_2 protected for 12h. The solution was dried through rotary evaporation and then neutralized with 10% HCl aqueous solution and the product was collected by filtration (1.41 g, yield: 80%).

^{1}H-NMR (400 MHz, DMSO-d6) δ 7.80(d, 1H), 7.45(s, 1H), 7.05 (d, 1H), 3.83 (d, 6H) 1.17 (m, 18H).
^{31}P-NMR (400 MHz, DMSO-d6) δ 66.6 ppm. MW: 353.20.

Synthesis of mono-(ditertbutylphosphino)amine terephthalic acid ligand (L6)

The procedure was revised from synthesis of **L4** with mono-(ditertbutylphosphino) amino terephthalate (0.50 g, 1.46 mmol) as reactant.

^{1}H-NMR (400 MHz, DMSO-d6) δ 7.93 (s, 1H), 7.73 (d, 1H), 7.28 (d, 1H), 6.44 (d, 1H), 1.34-1.14 (m, 18H).
^{31}P-NMR (400 MHz, DMSO-d6) δ 63.2 ppm. MW: 325.36.

Synthesis of mono-(dicyclohexylphosphino)amine terephthalate ester ligand (Me-L7)

The NHPCyclo$_2$ ligand (**L7**) was synthesized according to the literature with slight modification.[2] In a 250 mL flask, Dimethyl amino terephthalic acid (1.04 g, 5 mmol), and triethyl amine (2 g, 20 mmol) were dissolved in 50 mL of dichloromethane. Then the reaction was cooled to 0 ˚C in an Ice bath. To the resulting suspension, Chlorodicyclohexylphosphine (2.3 g, 10 mmol) was added dropwise over 15 min. Then the reaction was stirred at room temperature under N_2 protected for 12h. The solution was dried through rotary evaporation and then neutralized with 10% HCl aqueous solution and the product was collected by filtration (1.52 g, yield: 75%).
^{1}H-NMR (400 MHz, DMSO-d6) δ 8.22 (d, 1H), 7.92 (d, 2H) 7.26 (d, 1H), 3.91 (d, 6H), 1.79 (m, 12H) 1.27 (m, 10H). ^{31}P-NMR (400 MHz, DMSO-d6) δ 41.7 ppm. MW: 405.44.

Synthesis of mono-(dicyclohexylphosphino)amine terephthalic acid ligand (L7)

The procedure was revised from synthesis of **L4** with mono-(dicyclohexylphosphino) amino terephthalate (0.30 g, 0.74 mmol) as reactant.
^{1}H-NMR (400 MHz, DMSO-d6) δ 9.06 (d, 1H), 8.36 (m, 1H), 7.97 (d, 1H), 7.38 (d, 1H), 1.74 (m, 12H) 1.27 (m, 10H). ^{31}P-NMR (400 MHz, DMSO-d6) δ 45.6 ppm. MW: 377.18.

Synthesis of mono-(diphenylphosphino) amine terephthalate ester ligand (Me-L8)

The NHPPh$_2$ ligand (**L8**) was synthesized according to the literature with slight modification.[2] In a 250 mL flask, Dimethyl amino terephthalic acid (1.04 g, 5 mmol), and triethyl amine (2 g, 20 mmol) were dissolved in 50 mL of dichloromethane. Then the reaction was cooled to 0 ˚C in an Ice bath. To the resulting suspension, Chlorodiphenylphosphine (2.2 g, 10 mmol) was added dropwise over 15 min. Then the reaction was stirred at room temperature under N_2 protected for 12h. The solution was dried through rotary evaporation and then neutralized with 10% HCl aqueous solution and the product was collected by filtration (1.77 g, yield: 90%).
^{1}H-NMR (400 MHz, CDCl3) δ 8.72 (d, 1H), 8.27 (d, 1H), 7.99 (d, 1H), 7.50-7.36 (m, 10H), 3.88 (d, 6H) . ^{31}P-NMR (400 MHz, CDCl3-d6) δ 23.7 ppm. MW: 393.38

Synthesis of mono-(diphenylphosphino)amine terephthalic acid ligand (L8)

The procedure was revised from synthesis of **L4** with mono-(diphenylphosphino) amino terephthalate (0.80 g, 2.03 mmol) as reactant.
^{1}H-NMR (400 MHz, DMSO-d6) δ 8.05 (d, 1H), 7.84-7.80 (m, 5H) 7.56-7.46 (m, 8H).
^{31}P-NMR (400 MHz, DMSO-d6) δ 17.8 ppm. MW: 365.08.

S2.3. Synthesis of UiO-66-NHPPh$_2$.

To a solution of **UiO-66** (50 mg, 0.03 mmol) in DMF (10 mL), 25 mg of NHPPh$_2$ (0.063 mmol) ligand was added to form a solution mixture and was then sonicated for 15mins. Then the solution was heated at 80 degrees overnight until the powder changes color from white to pale yellow. After ligand exchange, the powder was washed with DMF (10mL×3) and then soak in 0.1M HCl DMF solution at 80 degrees for 8 hours to remove the excess surface coordinated ligands. Finally, the powder was washed with DMF (10mL×3), acetone (10mL×3) and then dried under vacuum and 100 degrees overnight. 75 mg was collected, with 86% yield calculated from the UiO-66. Ligand exchange ratio was confirmed by digest NMR.
Details of digested NMR with ^{31}P external standard:
Metal-organic framework (MOF) materials were digested for ^{1}H NMR and ^{31}P NMR analysis by sonicating a small amount of MOF material (~3 mg) in conc. D_2SO_4 (5 drops) until all of the MOF disintegrated and a brown heterogeneous solution formed. Upon addition of $(CD_3)_2SO$ (0.5 mL) to this solution and further sonication, a homogeneous pale-yellow solution forms, which was analyzed by ^{1}H NMR spectroscopy. The molecular weights of **UiO-66-(L4)-(L8)** based on immobilized ligands were calculated by the ratio of the phosphine peak coming from the MOF solution against the ^{31}P peak of phosphonic acid D_2O solution of known concentration (0.01 mol/L, chemical shift at 0 ppm) added as an external standard in a capillary tube. We note that it is important to have a homogeneous solution of acid digested MOF of sufficient concentration for accurate integration of the signals.

S2.4. Synthesis of UiO-66-NHPPh$_2$-Ni.

To a mixture of **UiO-66-NHPPh$_2$** (100 mg) and Nickel(II) chloride ethylene glycol dimethyl ether complex (25 mg), 10 mL of methanol was added into the vial and mixed uniformly at room temperature overnight. The solution changed from white to pale green. Furthermore, the solution was washed several times with DMF and then with acetone. Finally, the **UiO-66-NHPPh$_2$-Ni** catalyst obtained was weighed and stored in a vial as light

green powder. Nickel to Zirconium ratio was confirmed by ICP-MS.

S2.5. Synthesis of UiO-66-L1-3.

UiO-66-N_3 MOF with average size of 14 nm was synthesized according to a previously published procedure by Morris et al.[1] Then as synthesized MOF (500 mg, 0.26 mmol) was added to DMF (2 mL) and sonicated for 60 min. After sonication, $CuSO_4.5H_2O$ (6.5 mg, 0.026 mmol) was added to the mixture followed by sodium ascorbate (11 mg, 0.05 mmol). Then, propargyl alcohol (**L1**) (45 mg, 0.78 mmol) or 2-ethynylpyridine (**L2**) (81 mg, 0.78 mmol) or 4-Ethynyl-2,2'-bipyridine (**L3**) (141 mg, 0.78 mmol) were added in DMF (2 mL) to the reaction mixture. The reaction mixture was allowed to stir at 80°C for 72 hours. The resulting brown solid was collected by centrifugation (10000 rpm, 30 min), washed with fresh DMF (20 mL) and centrifuged (10000 rpm, 30 min), after which DMF was decanted. The DMF wash was repeated 2 times. The MOF collected after centrifugation was then placed in acetone and sonicated for 30 min and collected by centrifugation (10000 rpm, 30 min). The acetone wash was also repeated 2 times. The collected solid was then dried under vacuum at 70°C. The collected products were labeled as UiO66(**L1**), UiO-66(**L2**) and UiO-66(**L3**) respectively.

S2.6. Catalyst reactivity test of UiO-66-NHP-Ni for ethylene dimerization reaction

The dimerization product is analyzed by the Bromination method[3]. In the nitrogen glovebox, a 20mL glass vial was charged with 6mg (0.2 µmol) UiO-66-NHPiPr_2-Ni (precatalyst), 10mL Heptane, 40µl (20 euqiv.) Et_2AlCl (activator), and a stir bar. Then the vial was taken out and sonicated for at least 1 hour for activation. The solution was then transferred into the Parr reactor in the glovebox. The reactor was then tightly sealed, transferred out of the glovebox, and pressurized with ethylene (5, 10, 15, 20, 25 bar as required). The pressure was maintained at the specified pressure by keeping the reactor connected to the ethylene tank and stirred in the water bath as set temperature. After the specified amount of time was up, the reaction was cool in acetone dry ice bath until less than 2 bar, opened to air, and then quenched with methanol. The resulting solution was then treated with liquid Bromine until color changed yellow. Then the solution was added Na_2S_2O3 solution until color changed colorless again. The worked-up product was analyzed by GC-FID. The GC spectra of brominated standards are shown in **Figure SI**.

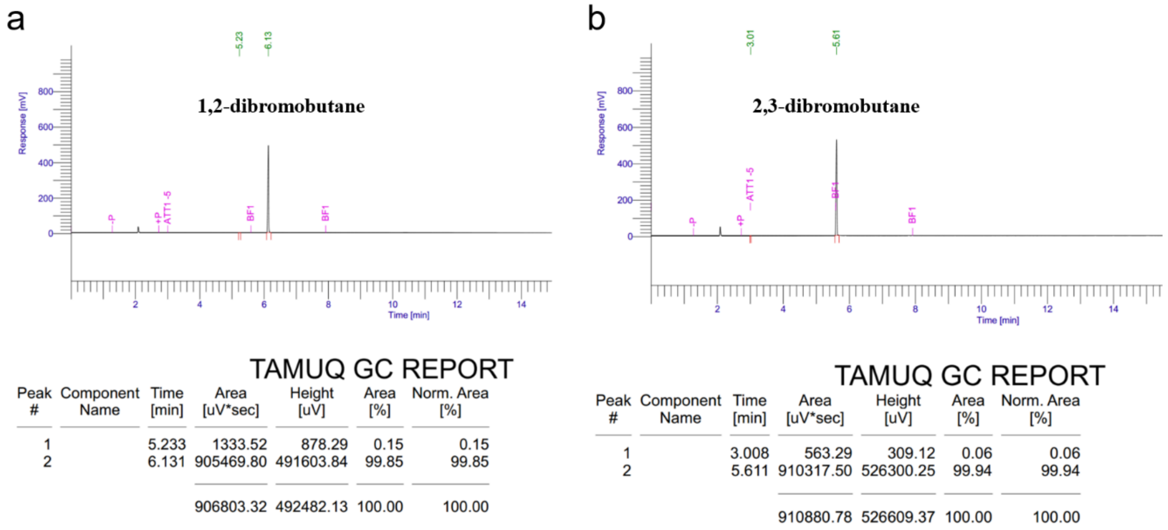

TAMUQ GC REPORT

Peak #	Component Name	Time [min]	Area [uV*sec]	Height [uV]	Area [%]	Norm. Area [%]
1		5.233	1333.52	878.29	0.15	0.15
2		6.131	905469.80	491603.84	99.85	99.85
			906803.32	492482.13	100.00	100.00

TAMUQ GC REPORT

Peak #	Component Name	Time [min]	Area [uV*sec]	Height [uV]	Area [%]	Norm. Area [%]
1		3.008	563.29	309.12	0.06	0.06
2		5.611	910317.50	526300.25	99.94	99.94
			910880.78	526609.37	100.00	100.00

Figure SI. GC spectra of standard 1,2-dibromobutane (a.) and 2,3-dibromobutane (b.)

Page 1 of 1

Software Version	:	6.3.4.0700	Date	:	2/8/2024 3:07:41 PM
Operator	:	user	Sample Name	:	VYP460BRO
Sample Number	:	001	Study	:	VYVYP460_bro
AutoSampler	:	BUILT-IN	Rack/Vial	:	0/1
Instrument Name	:	Clarus 500	Channel	:	A
Instrument Serial #	:	None	A/D mV Range	:	1000
Delay Time	:	0.00 min	End Time	:	21.00 min
Sampling Rate	:	12.5000 pts/s			
Sample Volume	:	1.000000 ul	Area Reject	:	0.000000
Sample Amount	:	1.0000	Dilution Factor	:	1.00
Data Acquisition Time	:	2/8/2024 2:21:35 PM	Cycle	:	1

Raw Data File : C:\GC DATA\Veeranna\VYP460BRO_001_001.raw
Inst Method : C:\GC METHOD\Veeranna_Organic Compound_300 C_AS_06022023 from C:\GC DATA\Veeranna\VYP460BRO_001_001.raw
Proc Method : C:\GC METHOD\Veeranna_Organic Compound_300 C_AS_06022023.mth from
Calib Method : C:\GC METHOD\Veeranna_Organic Compound_300 C_AS_06022023.mth from
Report Format File: C:\GC METHOD\GC FID Report.rpt
Sequence File : C:\GC METHOD\VYSEQUENCE _822024.seq

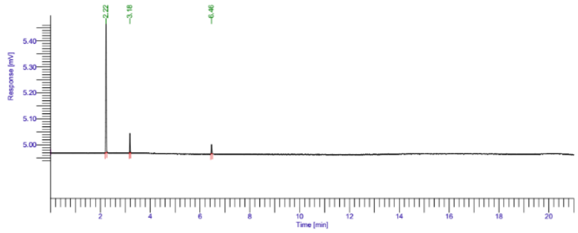

GC FID Report

Peak #	Component Name	Time [min]	Area [uV*sec]	Height [uV]	Area [%]
1		2.225	597.57	495.96	80.28
2		3.184	89.16	74.70	11.98
3		6.461	57.62	35.11	7.74
			744.34	605.78	100.00

Figure SII. GC spectra of ethylene dimerization reaction catalyzed by **UiO-66-NHPiPr$_2$(L5)-Ni** at 25°C, 15 bar (no byproduct was found with 1,2-dibromobutane only at 6.46 min).

S2.7. Catalyst reactivity test of UiO-66-NHP-Ni for ethylene dimerization reaction under gas phase and recyclability test

Inside a N_2-filled glovebox, UiO-66-NHPiPr$_2$-Ni (6 mg, 2 µmol, 1.6 µmol of Ni) was crushed into a fine powder and combined with anhydrous heptane (15.0 mL) and Et_2AlCl (0.2 mL of 1M solution in heptane, CAUTION: pyrophoric liquid) in a 30 mL pressure vessel (Parr Instruments Series 4714) fitted with a pressure gauge and containing a magnetic stir bar. The pressure vessel was closed and taken out of the glovebox. To ensure complete activation of the catalyst before the gas-phase reaction, the reaction mixture was allowed to stir for 1 h after which it was sonicated for another 1 h. The reaction vessel was then brought back inside the glovebox, the activated MOF was allowed to settle to the bottom of the vessel, and the supernatant was carefully decanted. The activated MOF was further rinsed with an aliquot of heptane (5.0 mL), the reactor was closed and taken out of the glovebox. Next, the pressure reactor was connected to a T-joint assembly together with an ethylene gas tank and a Schlenk line. The assembly was evacuated and the MOF in the reactor was allowed to dry under vacuum. at ambient temperature for 2 h. Afterward, the reaction was placed under a constant pressure (15 bar) of ethylene for 1 h. After completion, the reactor was closed from the ethylene source and quickly cooled in a dry ice/isopropanol bath to liquefy the oligomerization products and the pressurized ethylene was released. After opening the reactor, cold heptane (10.0 mL; pre-cooled in the dry ice/isopropanol bath) was quickly added to the reactor in a circular motion around the walls of the reactor to ensure that all the condensed oligomerization products were rinsed down to the bottom. An aliquot (1.0 mL) of the resulting solution was analyzed by GC-FID after bromination following the procedure described above for the liquid-phase reaction. In reuse experiments, the catalyst was isolated from the reaction mixture; a portion of it was removed for ICPOES analysis; and the remainder was taken back into the dry box, retreated with Et_2AlCl and reused for dimerization. The MOF crystals appeared to be covered by a white aluminum-oxide-like residue that we attributed to the decomposition of the large excess (20 equiv.) of Et_2AlClused for catalyst activation.

S2.8. Catalytic runs of UiO-66-NHPPh$_2$-Ni under flow conditions in a capillary microreactor

Inside a N_2-filled glovebox, UiO-66-NHPPh$_2$-Ni (260 mg) was crushed into a fine powder and activated overnight under vacuum at 70 C. The catalyst particles for reaction under microflow conditions were prepared

for loading into the stainless-steel micro reactor as a packed bed according to a previously described tableting/sieving procedure[3]. The synthesized **UiO-66-NHPPh$_2$-Ni** powder was tableted using press machine with diameter of 13 mm at 10-ton pressure for 1 minute. The obtained tablet was crushed using hand mortar and then sieved. Particle sizes in the range of 212 µm to 300 µm were collected for loading. Process was repeated several times until sufficient amount of powder in that target range was obtained (145 mg). Then powder was loaded in the stainless-steel tube (2.75 mm ID/ 3.175 mm OD) sealed with glass wool to obtain a packed column of length 4.2 cm. One end of the microreactor was connected to ethylene feed gas cylinder and the other end was connected to the inlet of GC-fid INSTRUMENT THROUGH PTFE TUBING. Then the column packed with the catalyst was filled with 6.0 mL of Heptane solution containing 0.5 ml (0.1 mol/L) of Et_2AlCl solution and left in contact with the catalyst for activation for 10 minutes. After the catalyst was activated, the fresh heptane solution was flushed through the column reactor to flush out activator and kept under the stream of nitrogen flow for another 10 minutes to remove the traces of the solvent. The column was flashed with Ethylene gas (flow rate 5mL/min) and immersed in a water bath maintained at 25 °C. The desired ethylene pressure was controlled through MFC (micro flow controller) and the dimerization products were passed through the pre-calibrated GC-FID instrument (SHIMADZU) to analyze the product distribution in-situ under flow conditions. During the reaction, the gaseous products coming out of the flow reactor was directly sent to the GC-FID instrument at regular intervals (20 min) to analyze the product distribution and the conversion to dimerization product was calculated based on the ethylene gas feed. The maximum conversion of 5 % was observed at 10 bar ethylene pressure at 25 °C (**Figure SII**).

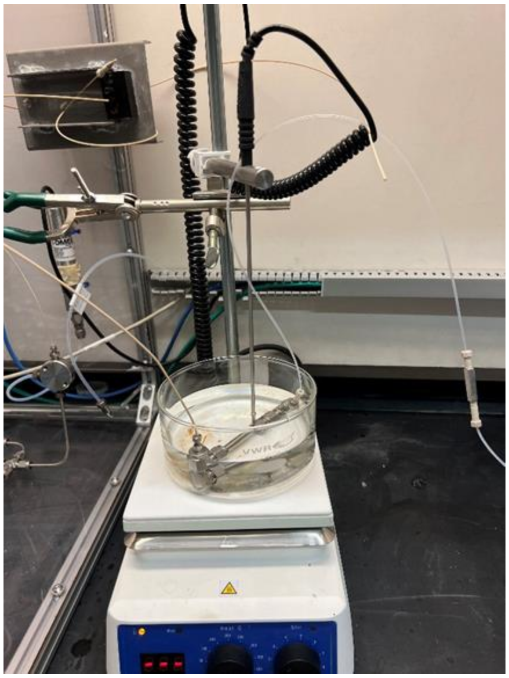

Figure SIII. Experimental setup and stainless-steel microreactor for flow reaction.

S2.9 Computation details.

All geometry optimizations of intermediates and transition states were achieved using spin-unrestricted UB3LYP[4]-D3[5]/def2-SVP[6]method, in heptane solvent using the CPCM solvent model[7] with "opt=noeigen" and "guess=mix" keywords as implemented in Gaussian16[8]. Frequency calculations were also conducted at the same level of theory to obtain vibrational frequencies to determine the identity of stationary points as intermediates (no imaginary frequencies) or transition states (only one imaginary frequency), as well as obtaining the thermochemistry: enthalpy (DH) and free energy (DG) at the temperature of 298 K. Also, extensive conformational search was performed for all the nickel intermediates and transition states, and only the lowest-energy species were shown and discussed. All structural figures were generated with CYLview[9]. Distances in structural figures are shown in Å and energies are in kcal/mol.

All the structures involved in each reaction mechanism are calculated at the uB3LYP-d3/def2-SVP-CPCM

(heptane). Cartesian coordinates (xyz format) are provided at the end of this document.

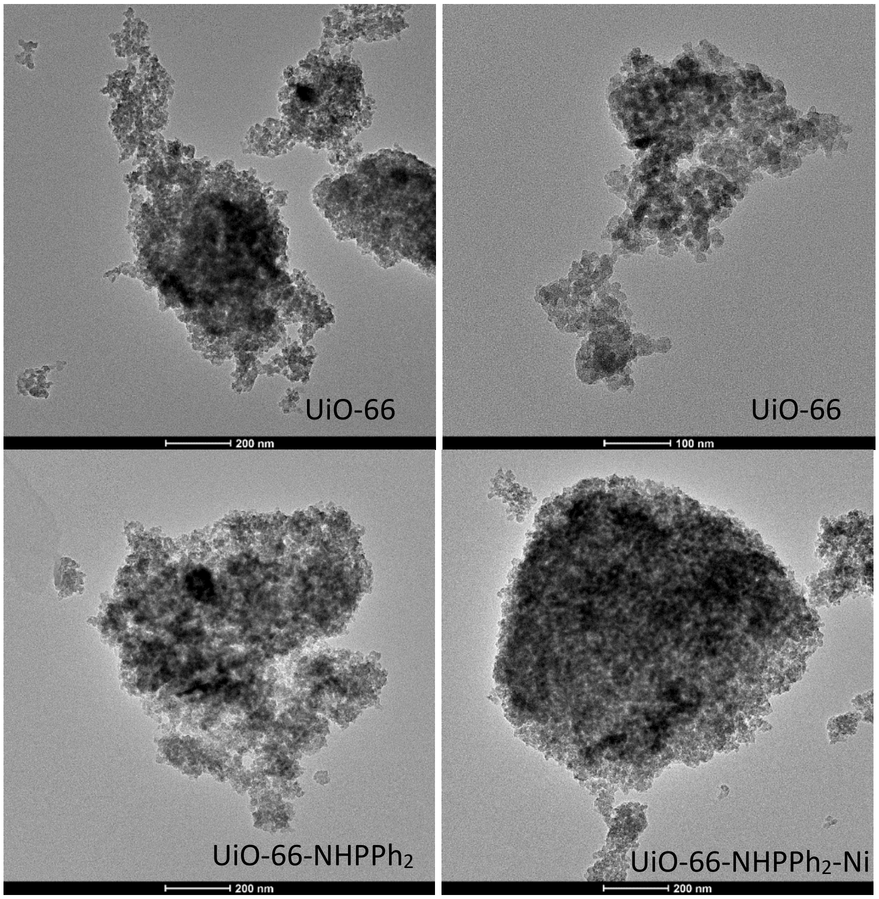

Figure S1. SEM **UiO-66, UiO-66-NHPPh$_2$** and **UiO-66-NHPPh$_2$-Ni**.

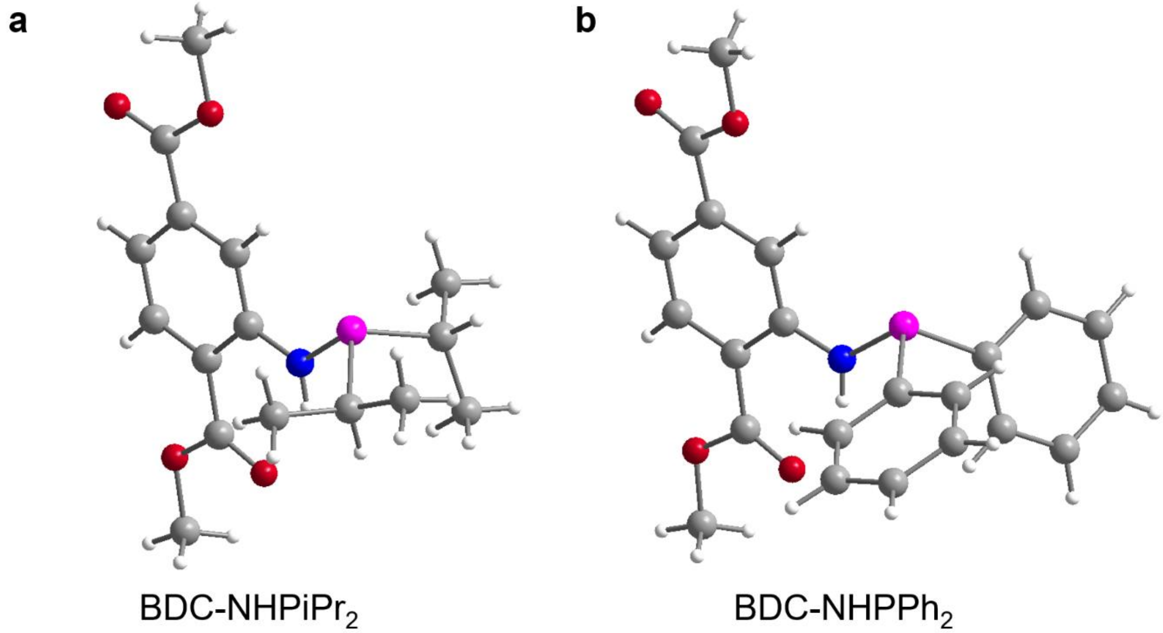

Figure S2. Schematic representation of crystal structure of mono-phosphine ester **BDC-NHiPr$_2$ (**a) and **BDC-NHPPh$_2$** (b) C:gray, O: red, N: blue, P: magenta, H: white.

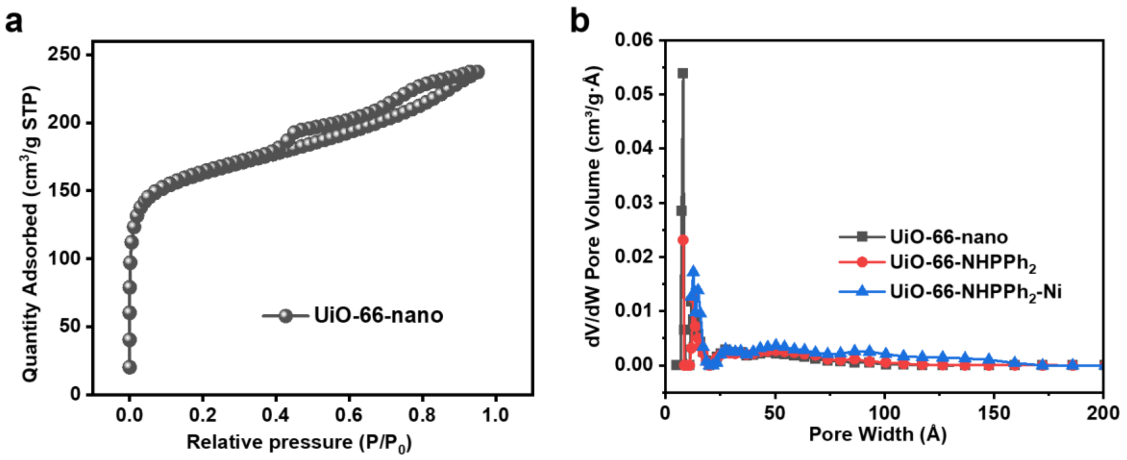

Figure S3. a. N_2 adsorption isotherm of **UiO-66-nano b.** Pore size distribution of **UiO-66-nano, UiO-66-NHPPh$_2$** and **UiO-66-NHPPh$_2$-Ni**

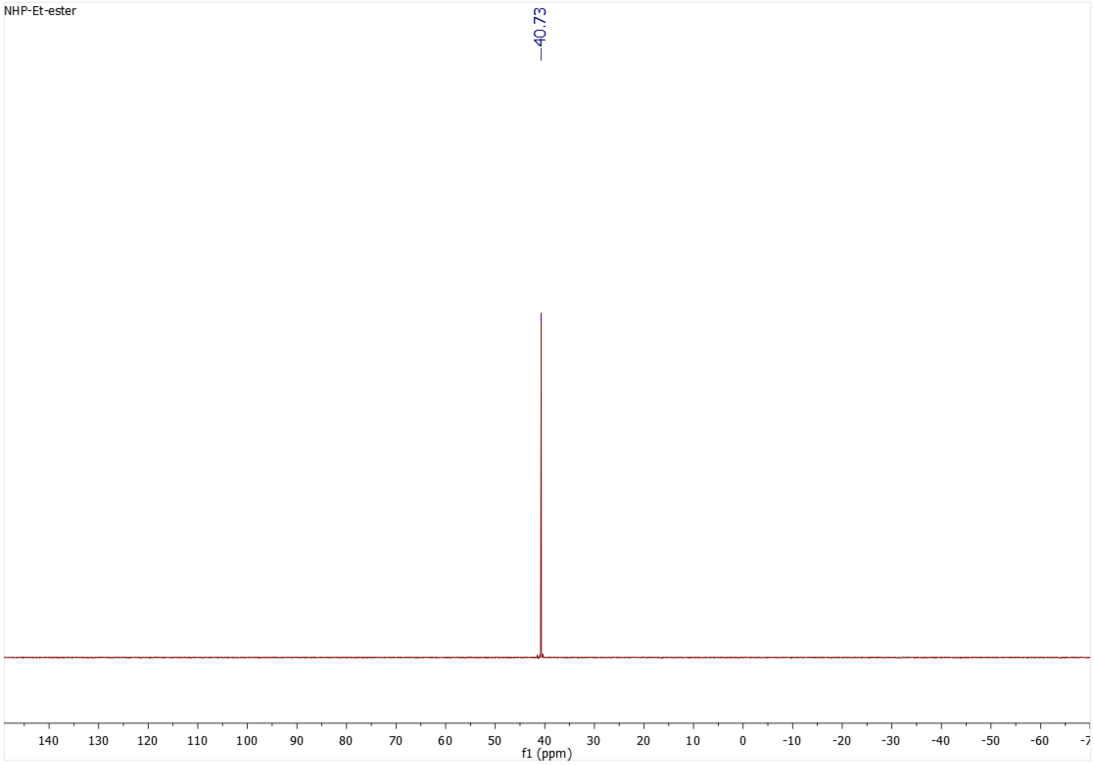

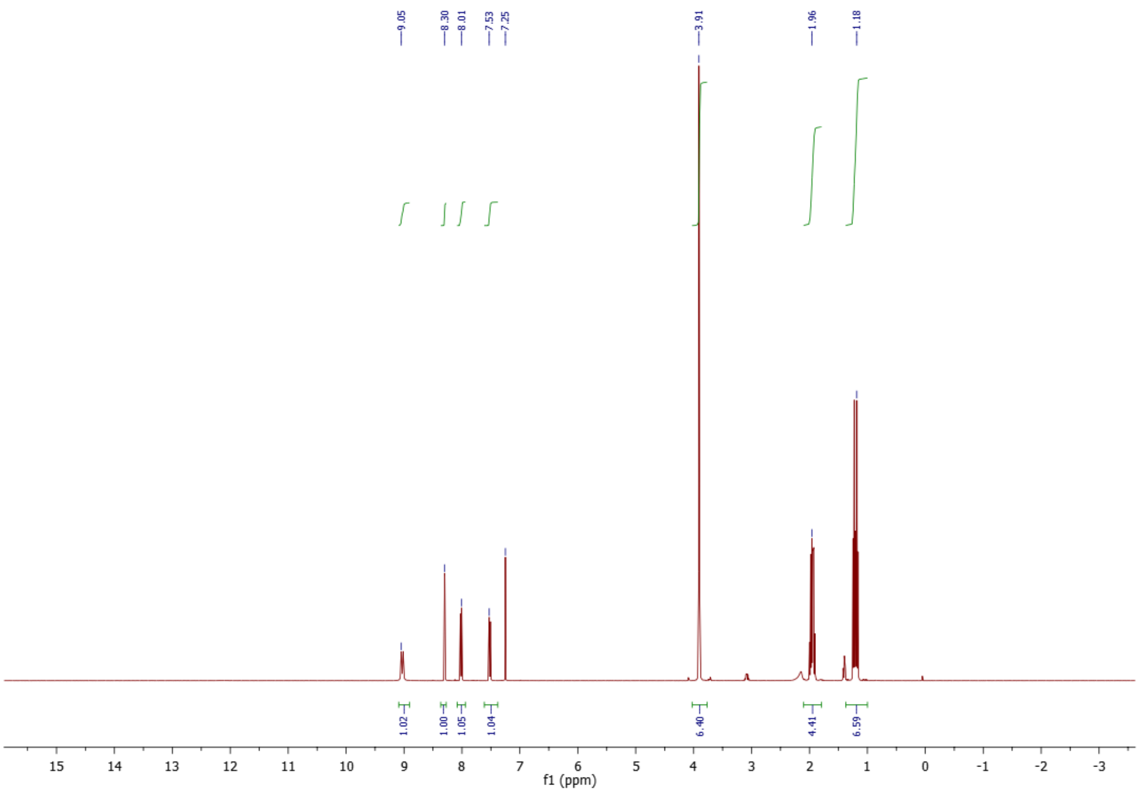

Figure S4. ^{31}P and ^{1}H NMR of **L4** Bis(diethylphosphino)amine ester ligand.

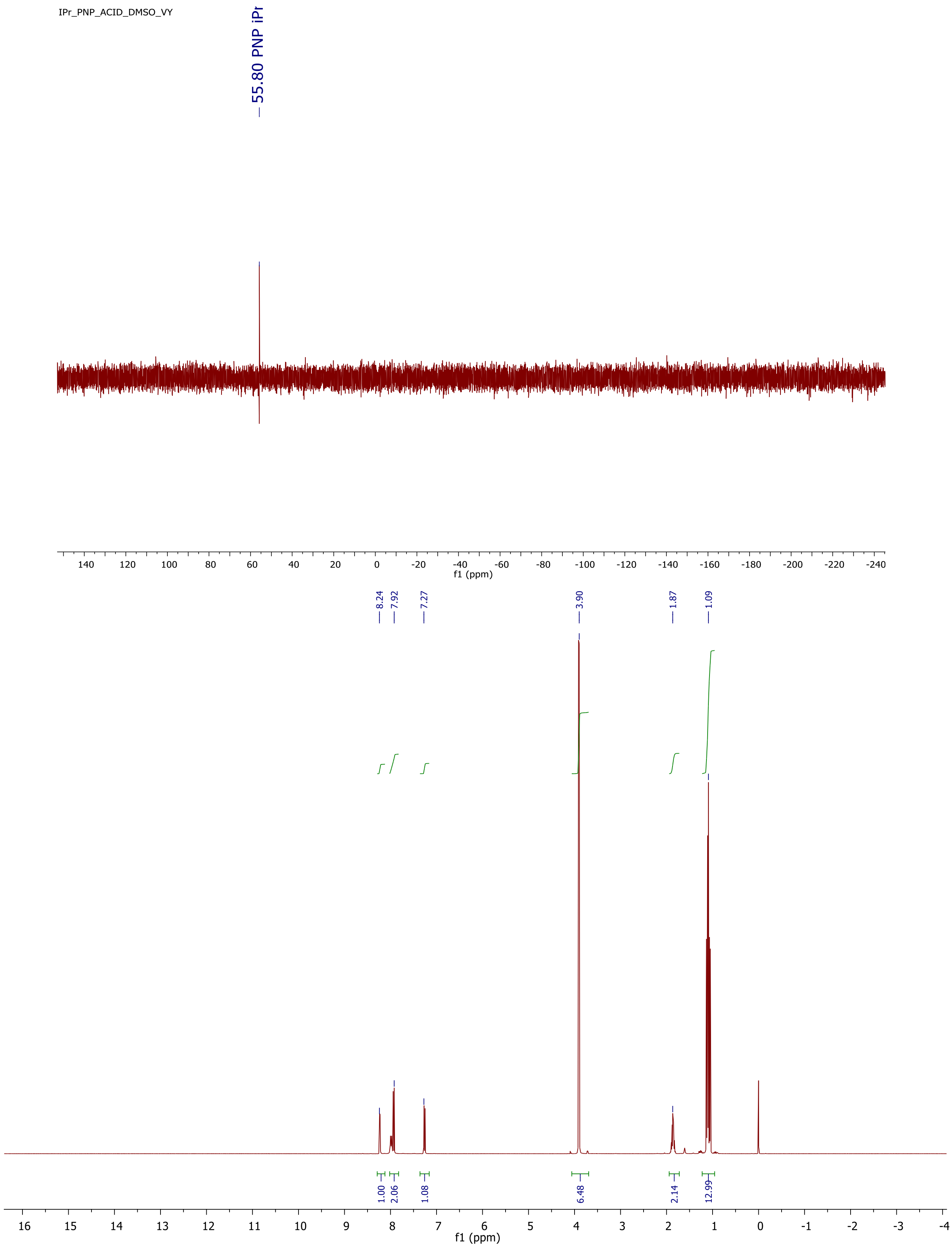

Figure S5. ^{31}P and ^{1}H NMR of **L5** Bis(diisopropylphosphino)amine ester ligand.

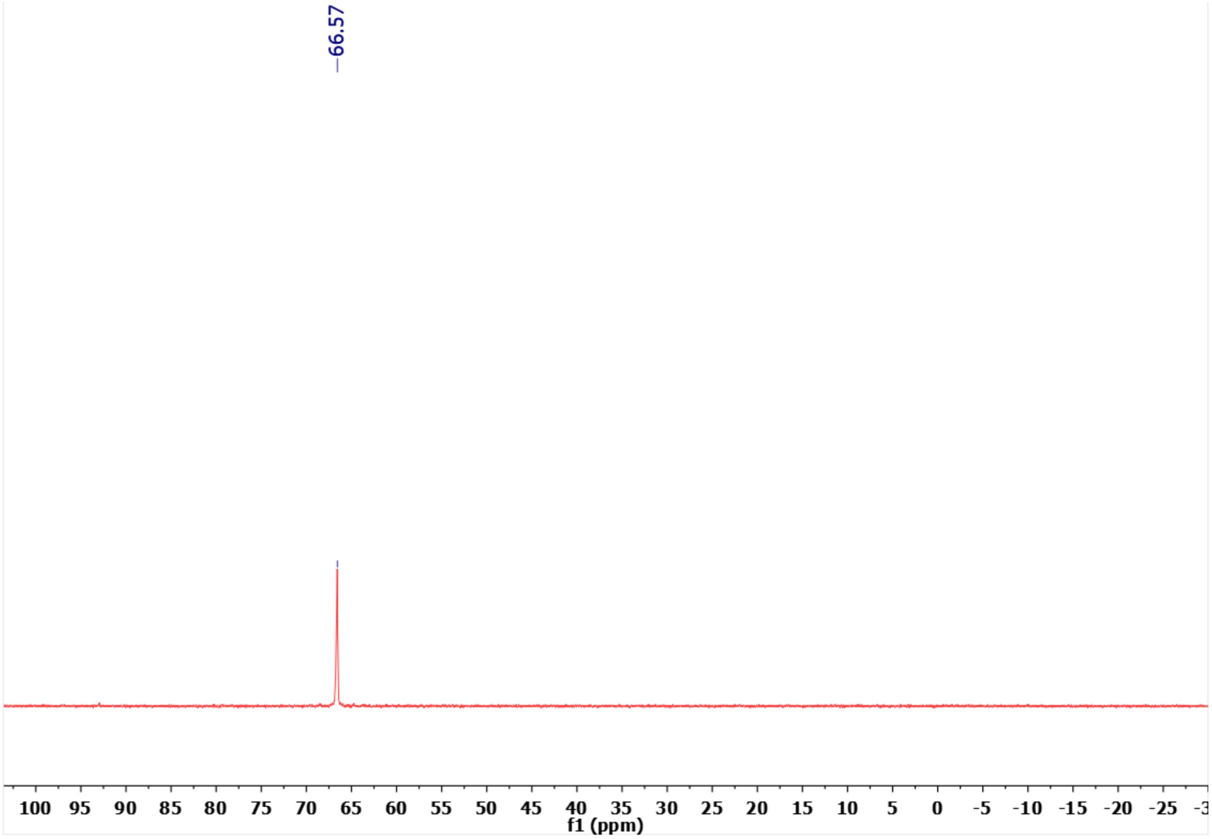

66.57
100 95 90 85 80 75 70 65 60 55 50 45 40 35 30 25 20 15 10 5 0 -5 -10 -15 -20 -25
f1 (ppm)

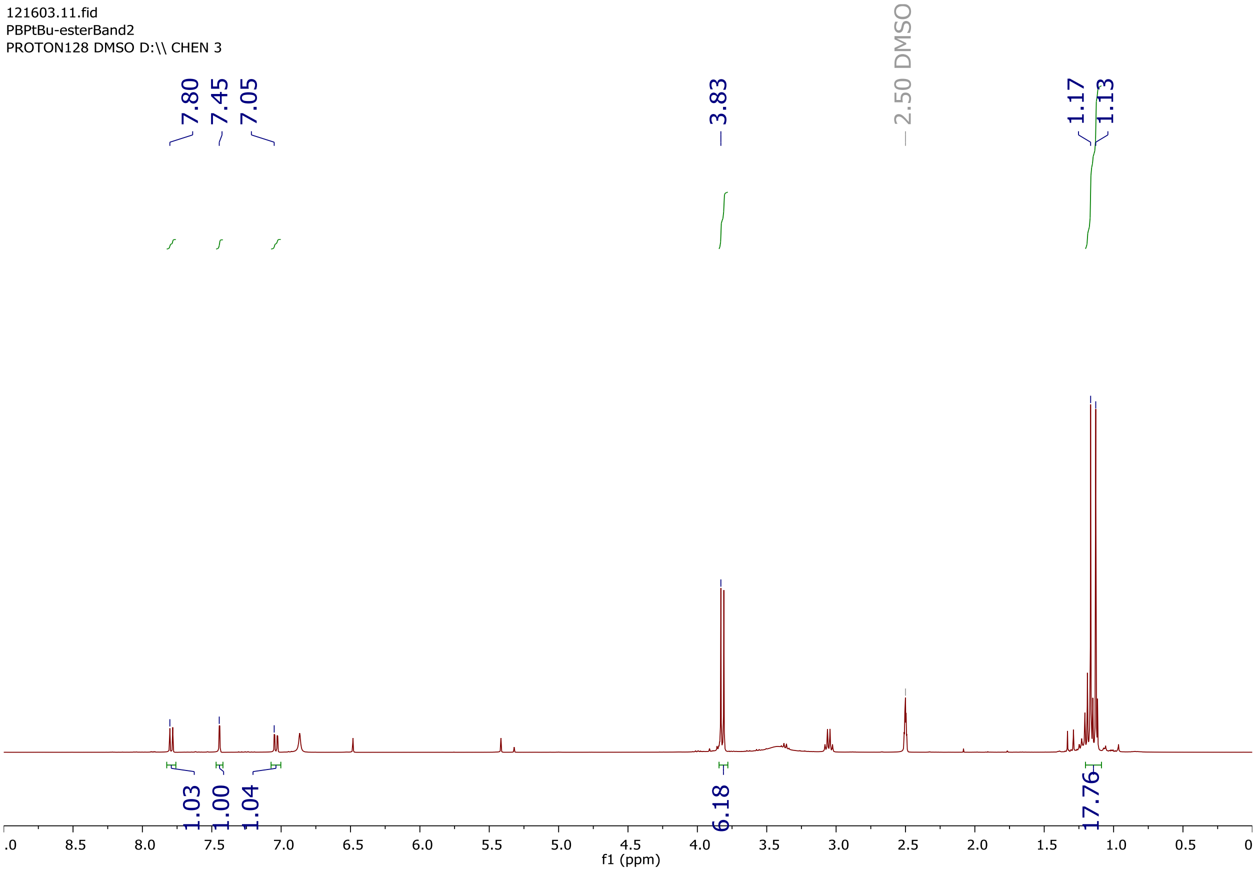

Figure S6. ^{31}P and ^{1}H NMR of **L6** Bis(ditertbutylphosphino)amine ester ligand.

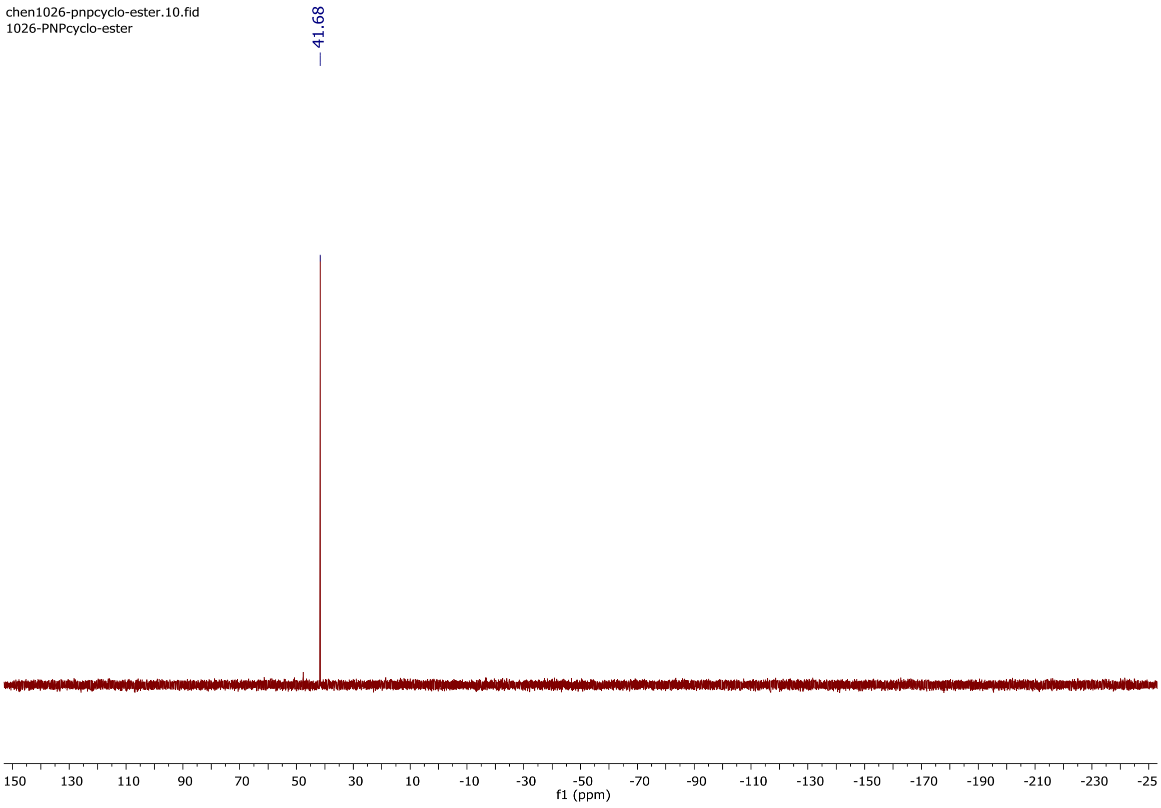

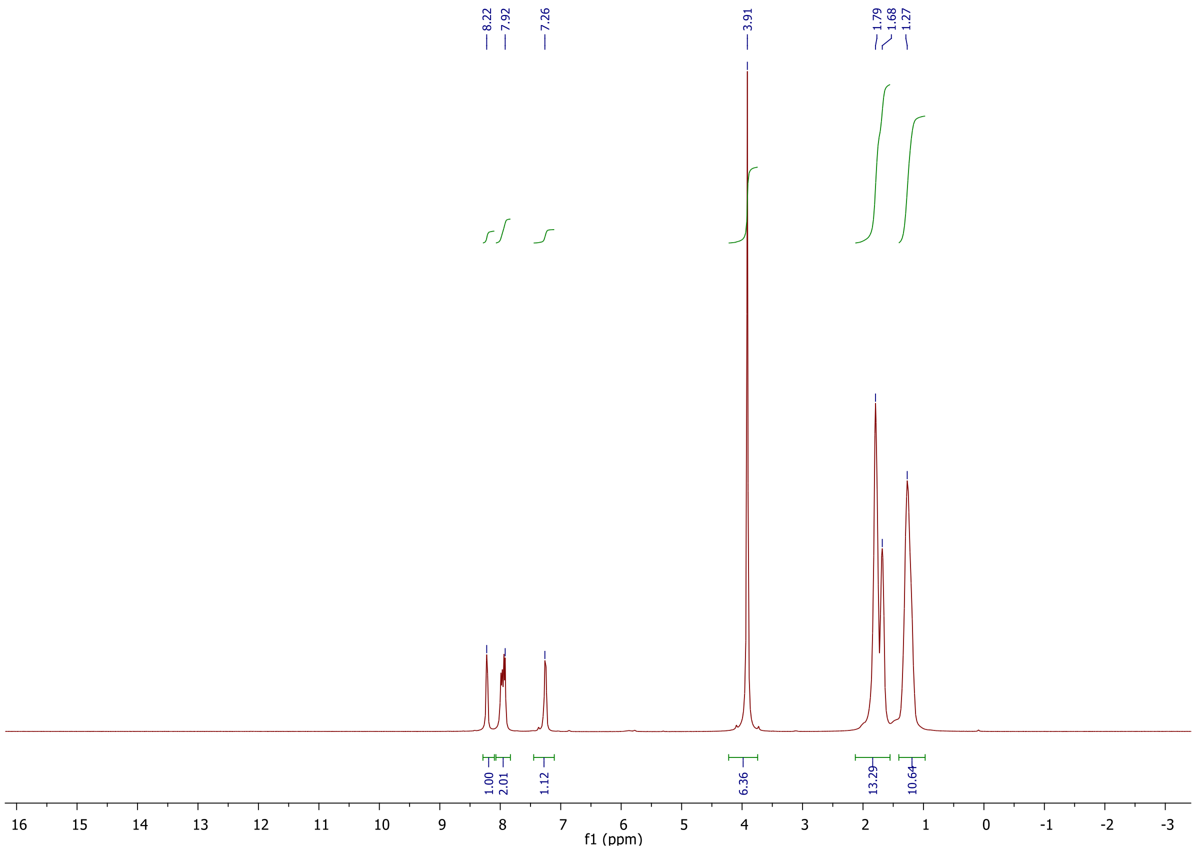

Figure S7. ^{31}P and ^{1}H NMR of **L7** Bis(dicyclohexylphosphino)amine ester ligand.

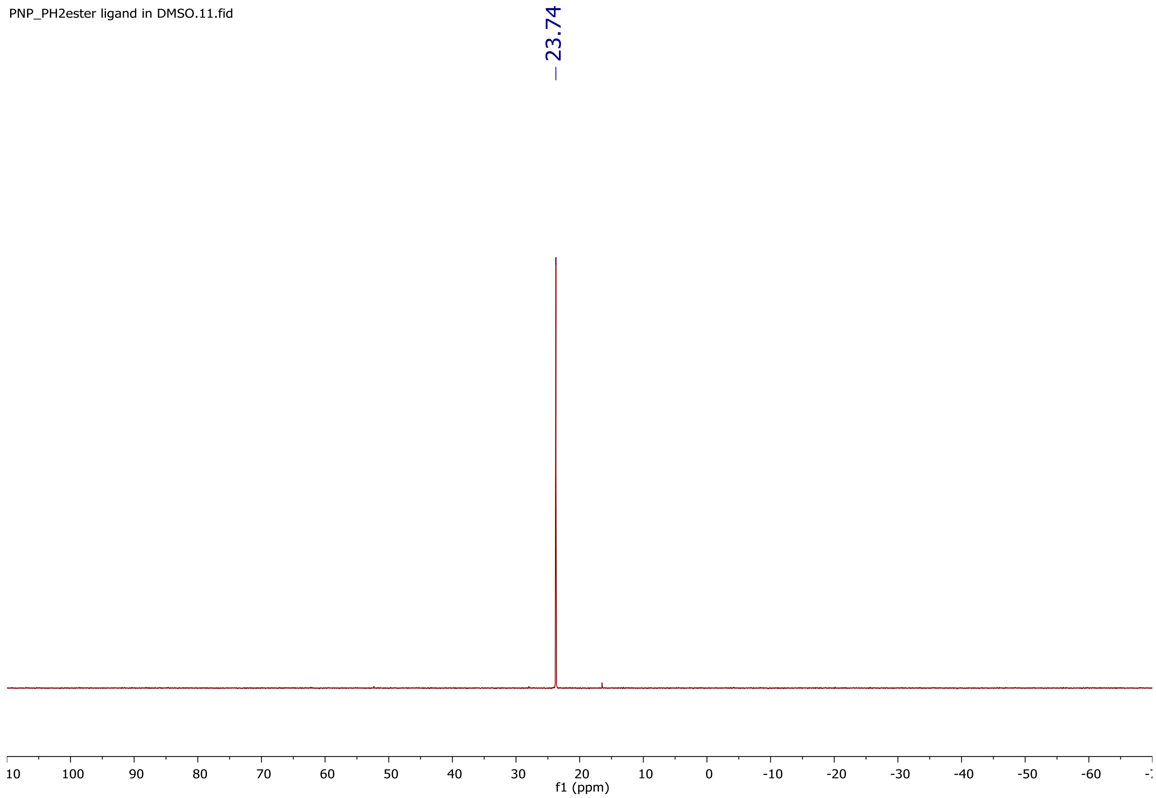

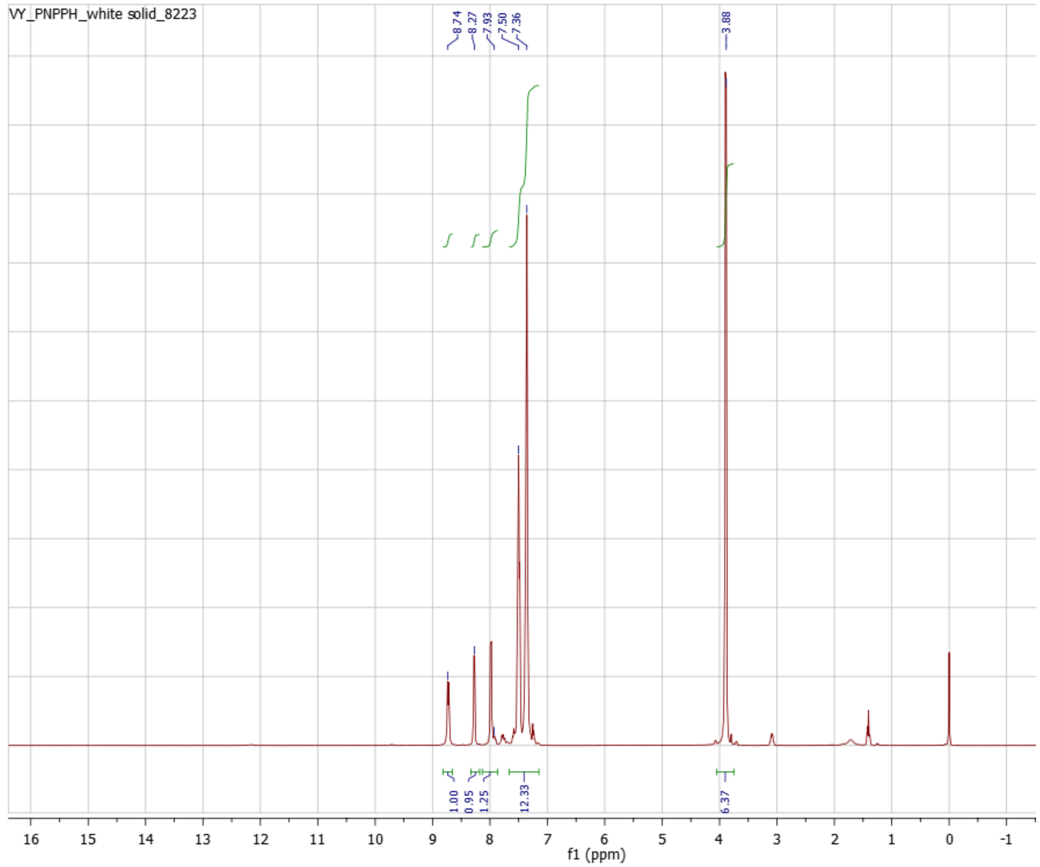

Figure S8. ^{31}P and ^{1}H NMR of **L8** Bis(diphenylphosphino)amine ester ligand.

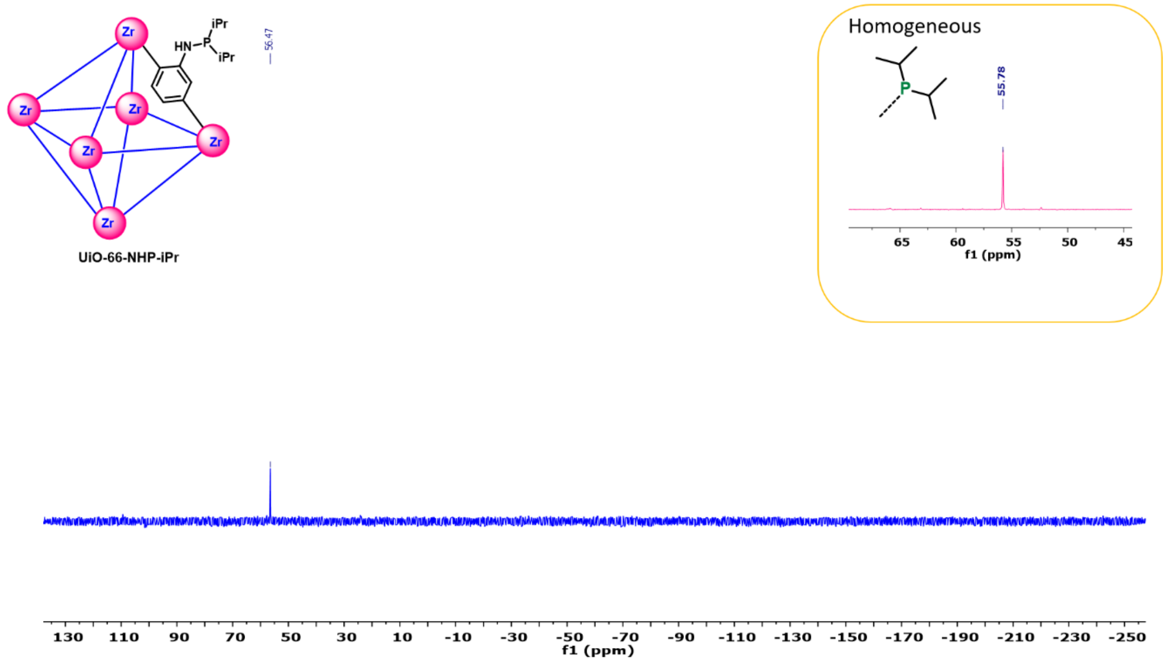

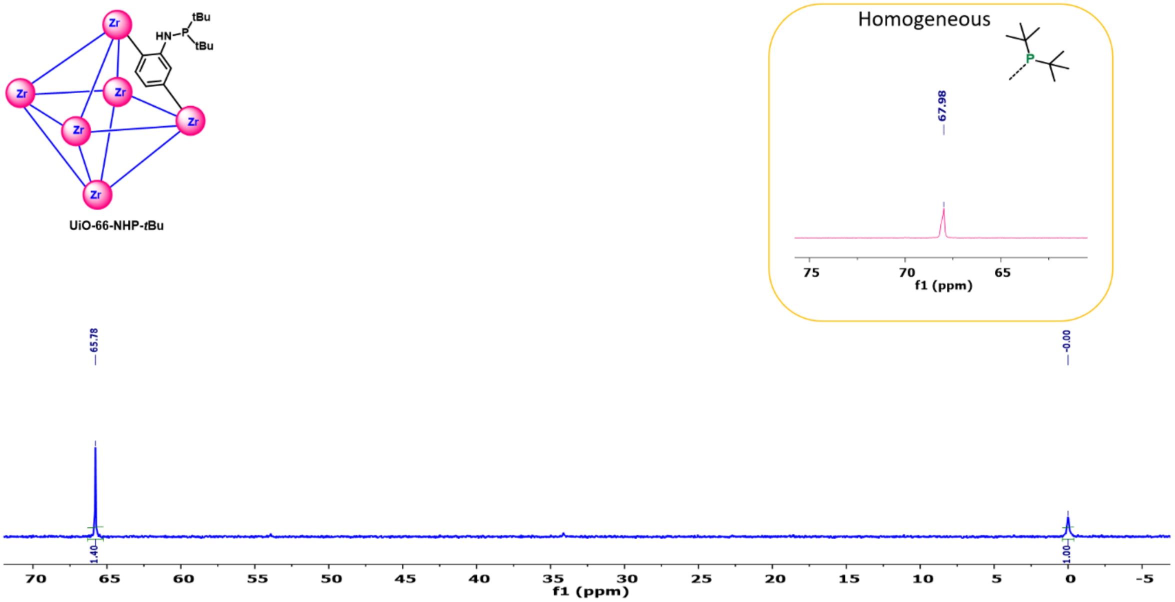

tBu
HN
P
tBu
Zr
UiO-66-NHP-tBu
Homogeneous
P
67.98
75
70
65
f1 (ppm)
65.78
-0.00
1.40
1.00
70
65
60
55
50
45
40
35
30
25
20
15
10
5
0
-5
f1 (ppm)

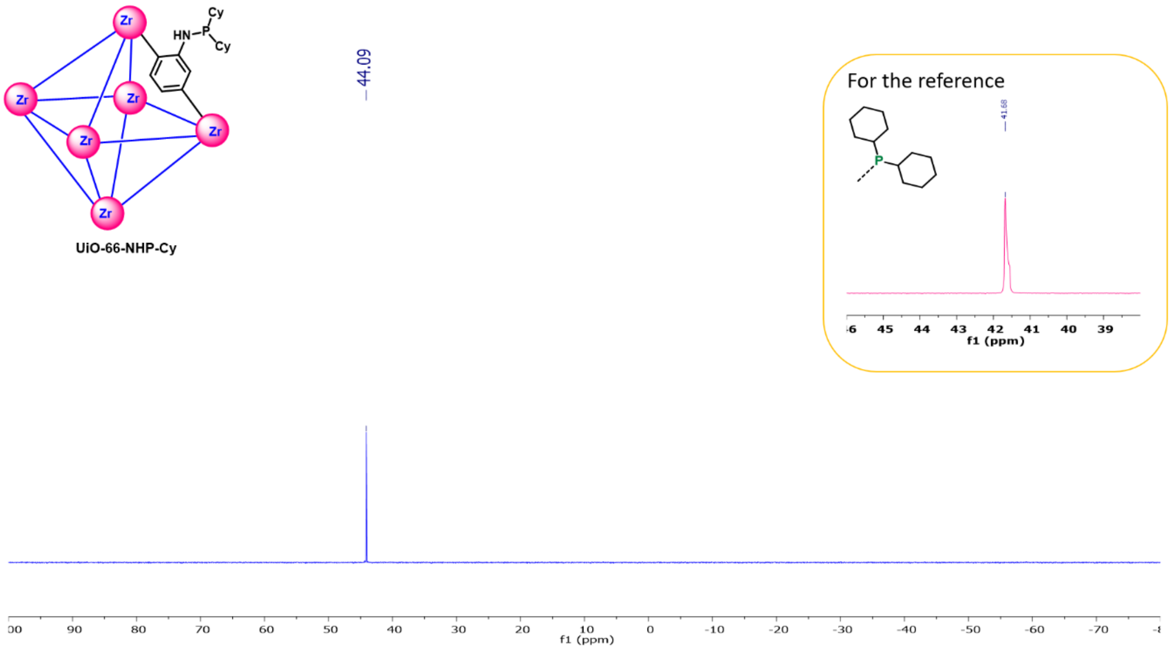

Cy
HN
P
Cy
Zr
UiO-66-NHP-Cy
44.09
For the reference
P
41.68
45
44
43
42
41
40
39
f1 (ppm)
90
80
70
60
50
40
30
20
10
0
-10
-20
-30
-40
-50
-60
-70
f1 (ppm)

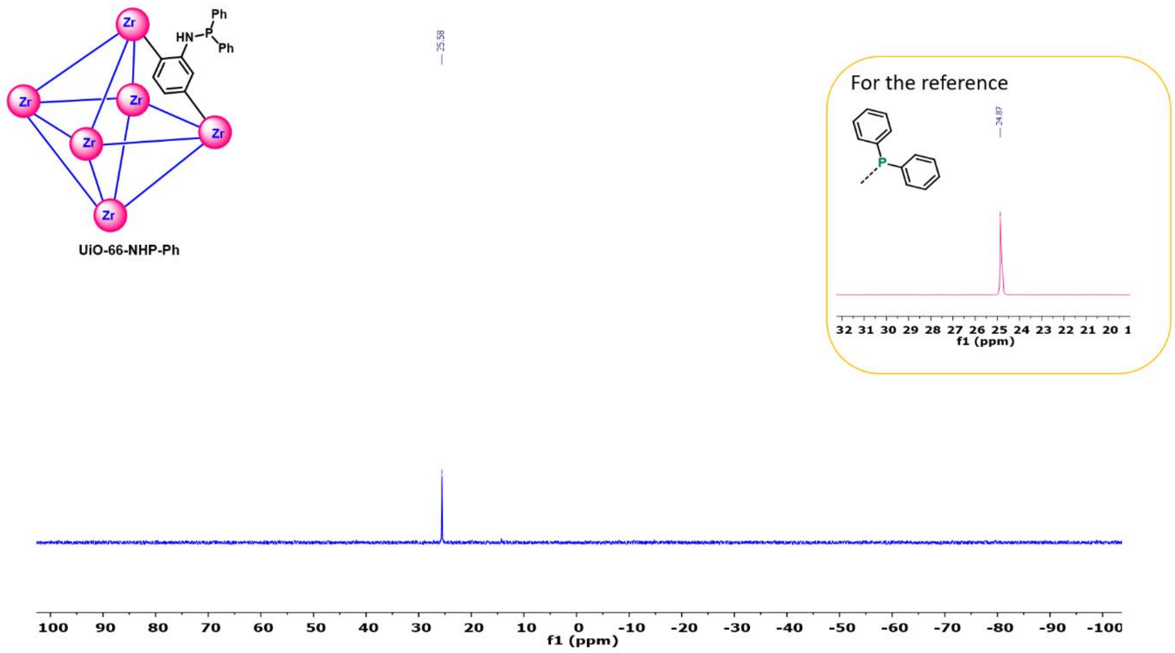

Figure S9. ^{31}P NMR of digested MOF sample for **L5-L8**.

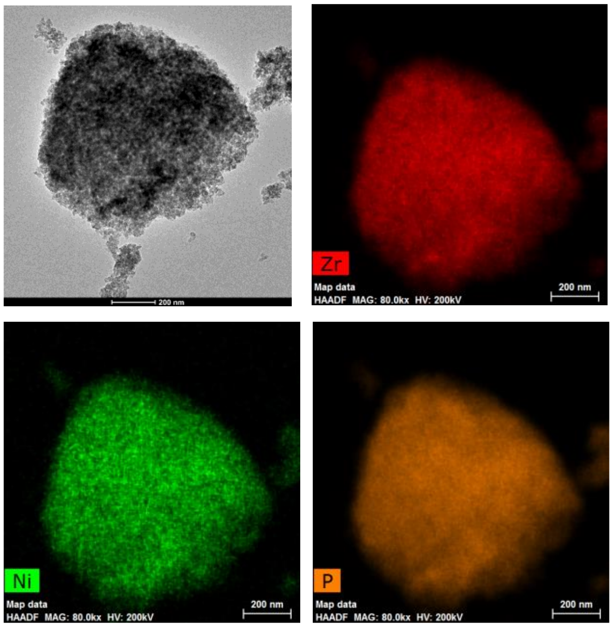

Figure S10. SEM and Zr, Ni and P elemental mapping pictures of **UiO-66-NHPPh$_2$-Ni**

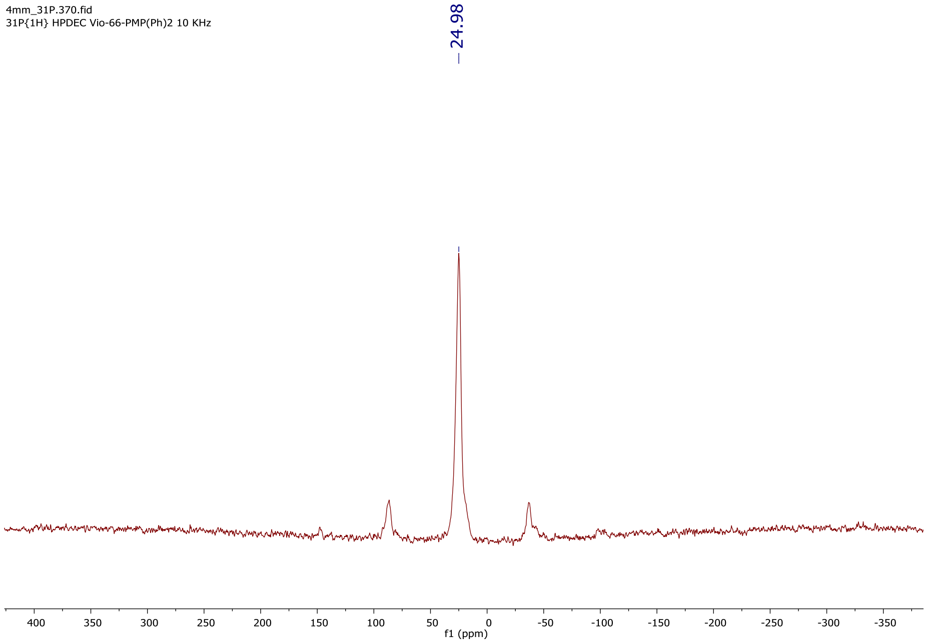

Figure S11. ^{31}P SSNMR of **UiO-66-NHPPh$_2$**

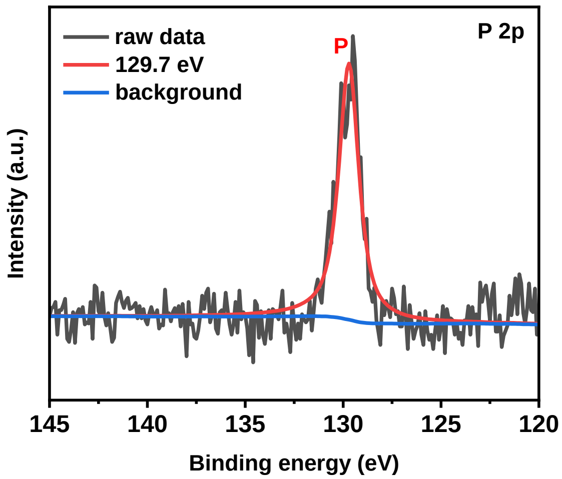

a.

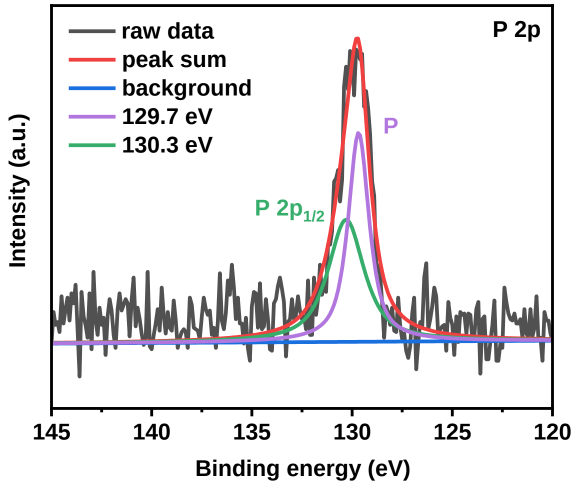

b.

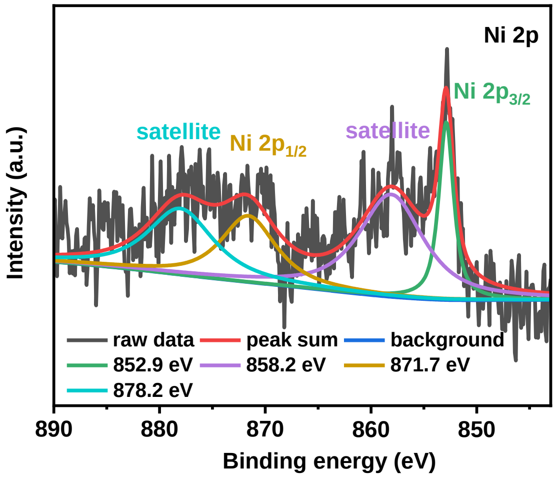

c.

Figure S12. XPS spectra for the analysis (a) P 2p of **UiO-66-NHPPh$_2$**, (b) P 2p and (c) Ni 2p of **UiO-66-NHPPh$_2$-Ni** components.

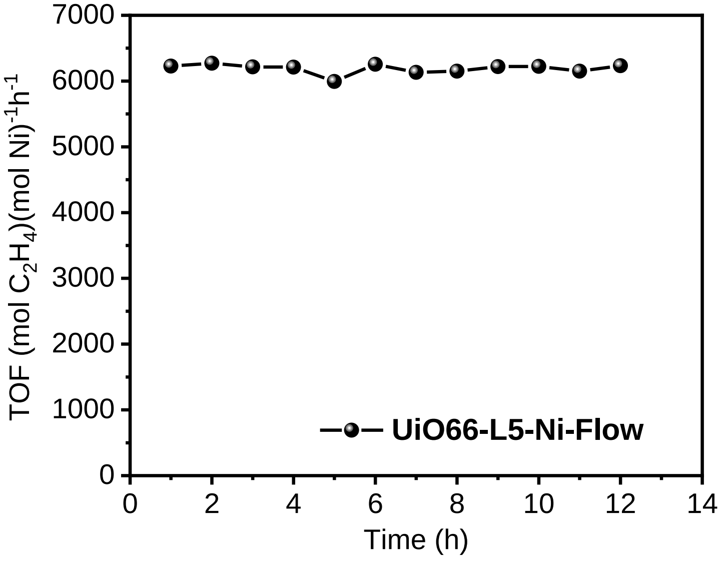

Figure S13. TOF over time of ethylene dimerization reaction in the micro-reactor under flow condition (10ml/min ethylene gas at room temperature).

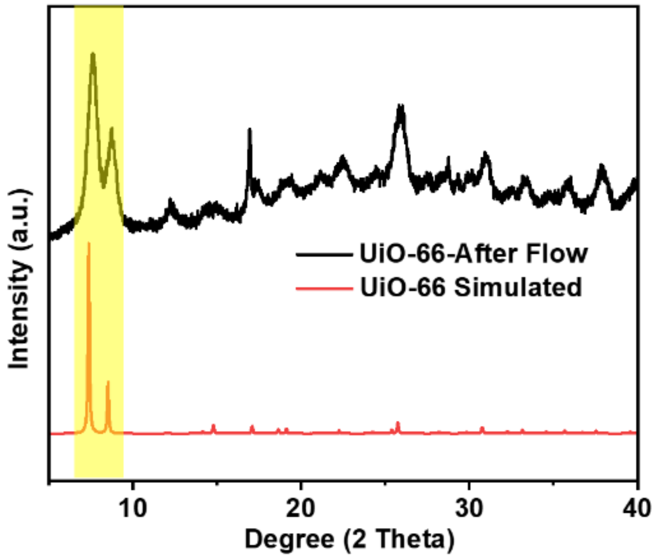

Figure S14: PXRD pattern of **UiO-66-L5-Ni** after flow ethylene dimerization.

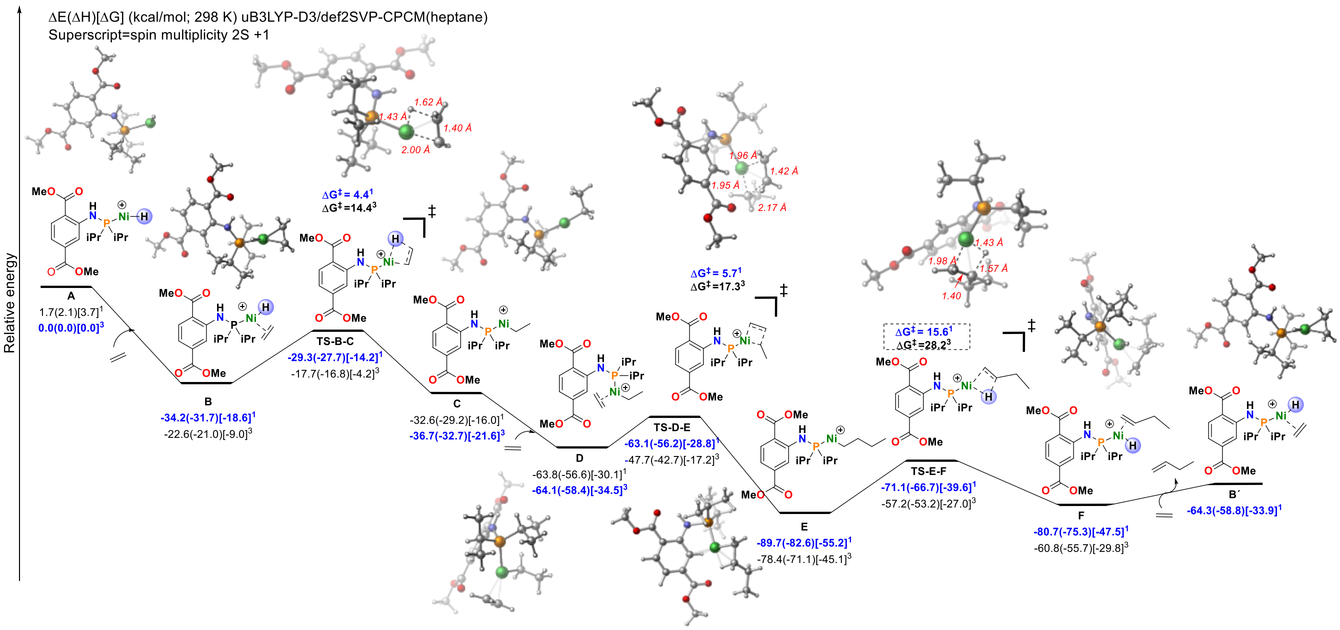

Figure S15. Calculated energetics for ethylene dimerization reactions using the complex **NHPiPr$_2$(L5)-NiCl$_2$**. The energies shown were computed at the uB3LYP-D3/def2SVP-CPCM(heptane) level of theory.

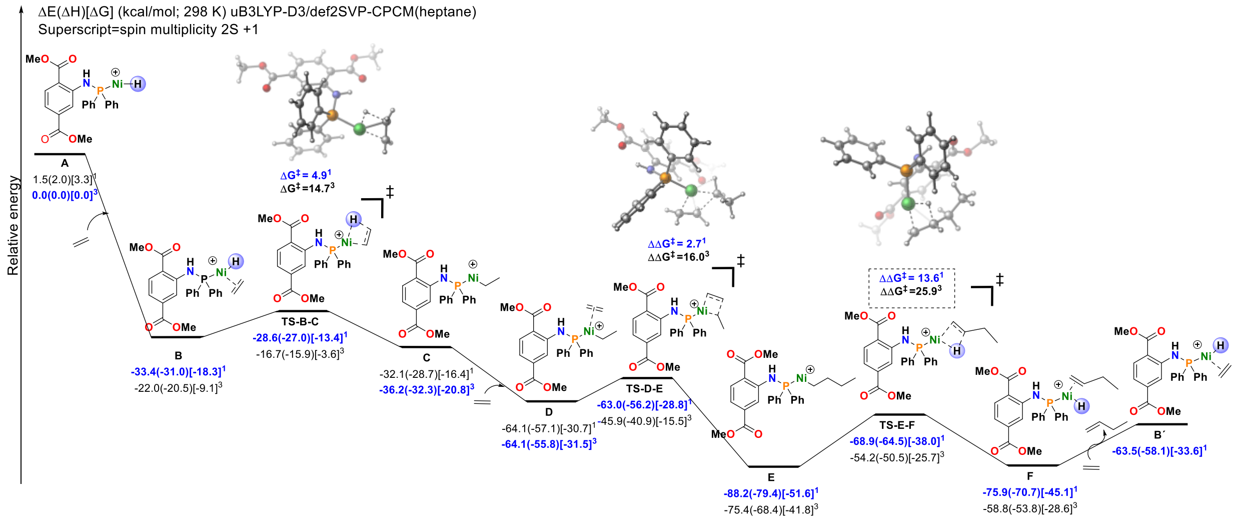

Figure S16. Calculated energetics for ethylene dimerization reactions using the complex **NHPPh$_2$(L8)-NiCl$_2$**. The energies shown were computed at the uB3LYP-D3/def2SVP-CPCM(heptane) level of theory.

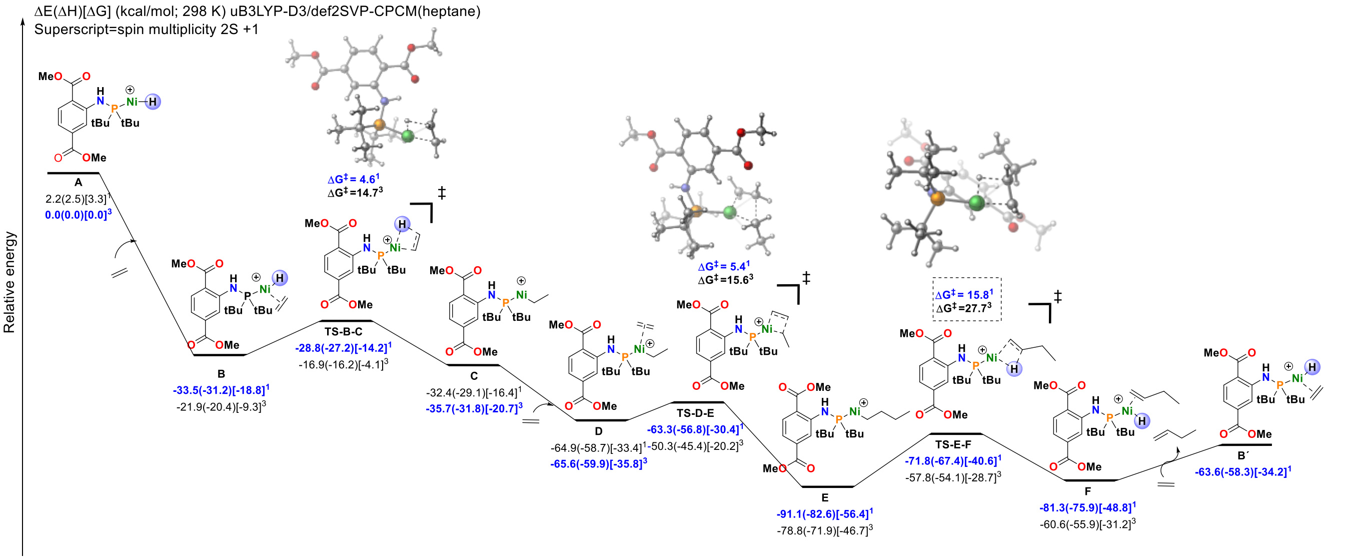

Figure S17. Calculated energetics for ethylene dimerization reactions using the complex **NHPtBu$_2$(L6)-NiCl$_2$.** The energies shown were computed at the uB3LYP-D3/def2SVP-CPCM(heptane) level of theory.

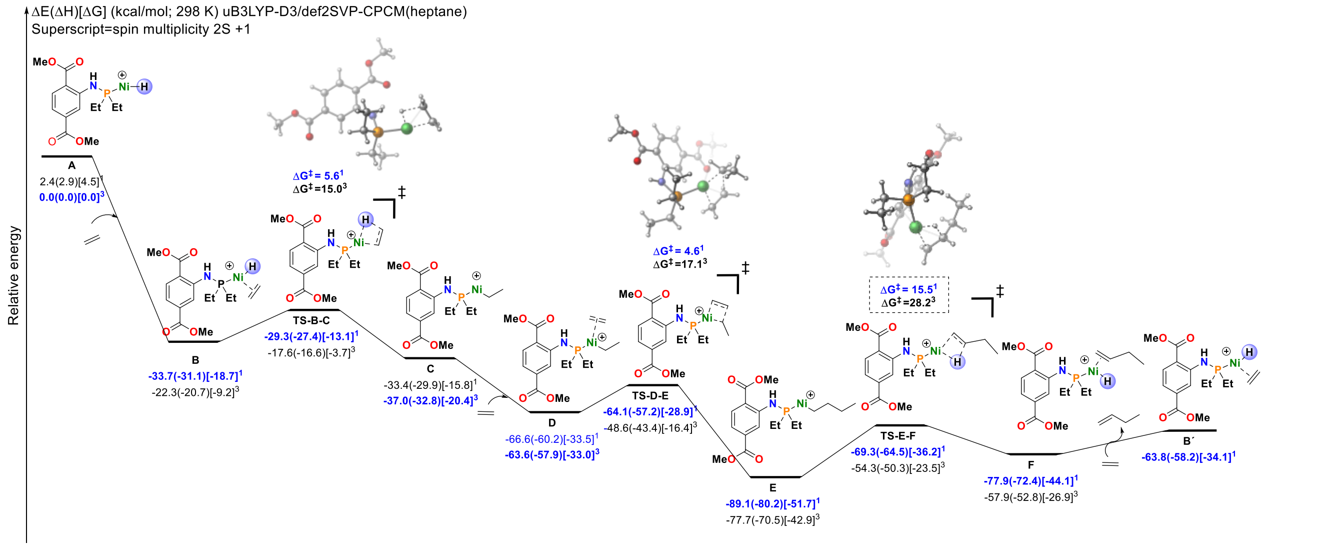

Figure S18. Calculated energetics for ethylene dimerization reactions using the complex **NHPEt$_2$(L4)-NiCl$_2$.** The energies shown were computed at the uB3LYP-D3/def2SVP-CPCM(heptane) level of theory.

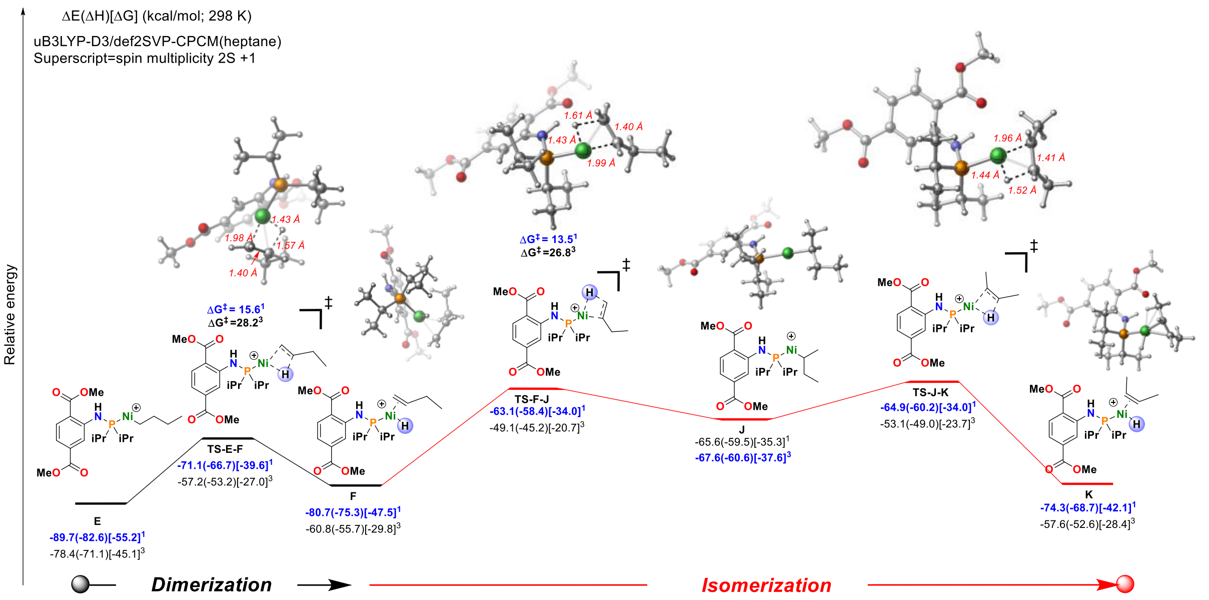

Figure S19. Calculated energetics for ethylene dimerization vs isomerization reactions using the complex **NHPiPr$_2$(L5)-NiCl$_2$**. The energies shown were computed at the uB3LYP-D3/def2SVP-CPCM(heptane) level of theory.

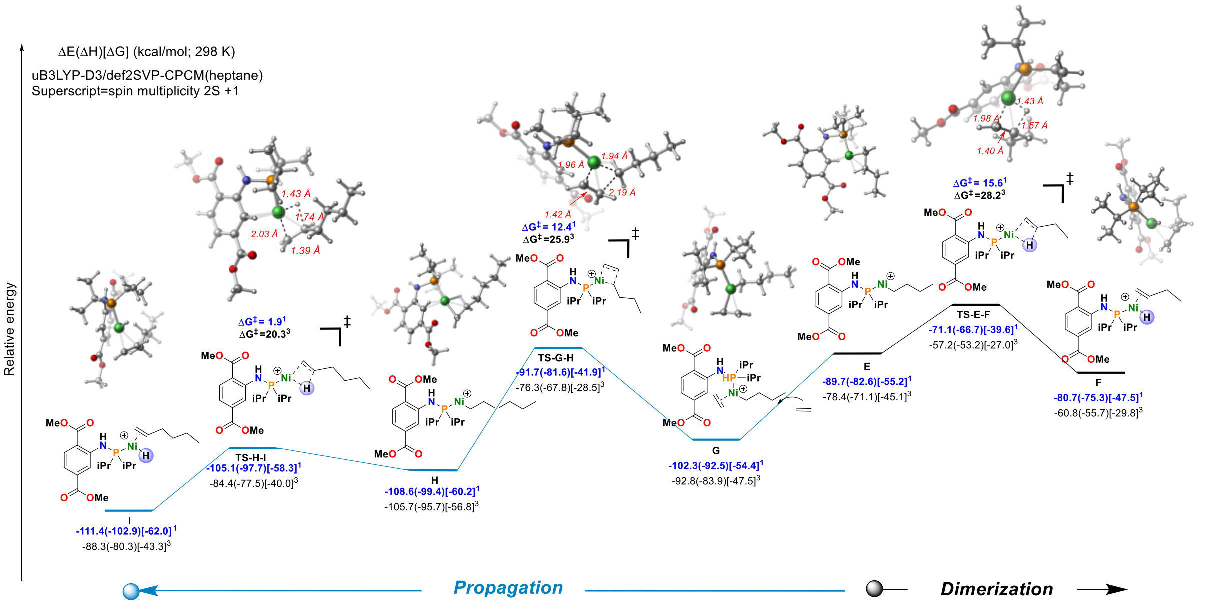

Figure S20. Calculated energetics for ethylene dimerization vs propagations reactions using the complex **NHPiPr$_2$(L5)-NiCl$_2$**. The energies shown were computed at the uB3LYP-D3/def2SVP-CPCM(heptane) level of theory.

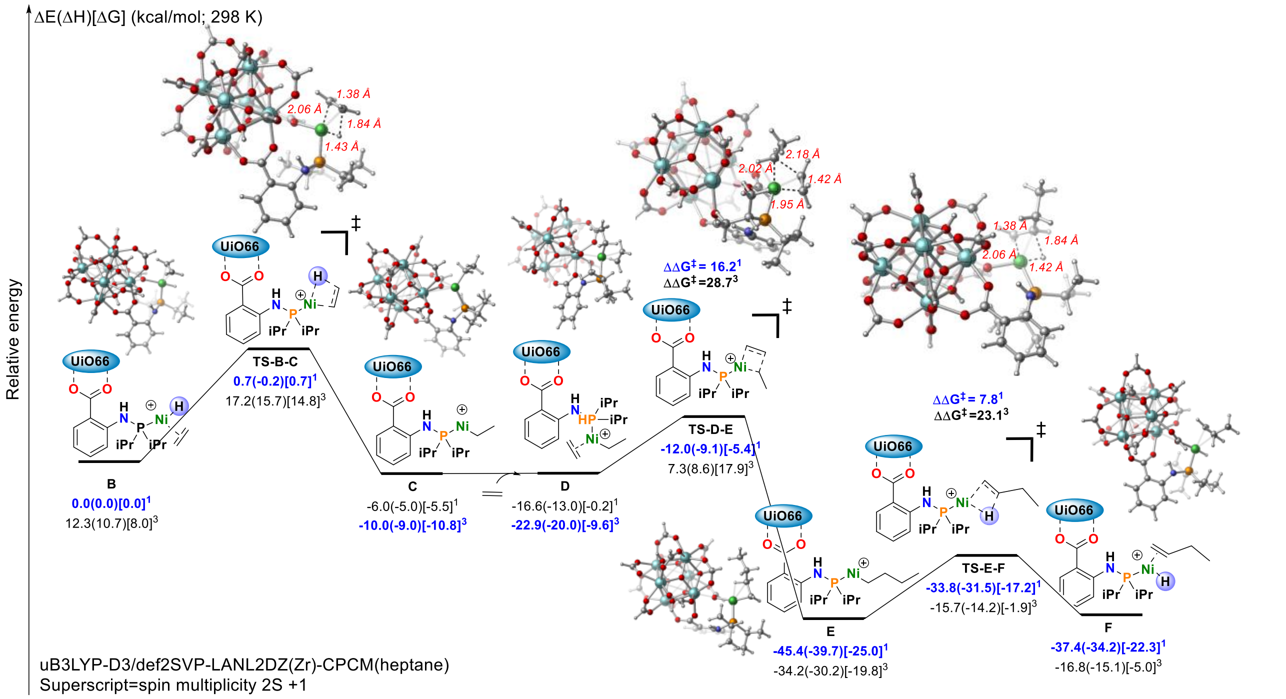

Figure S21. Calculated energetics for ethylene dimerization reactions using the complex **NHPiPr$_2$(L5)-NiCl$_2$** and truncated UiO-66. The energies shown were computed at the uB3LYP-D3/def2SVP-LANL2DZ-CPCM(heptane) level of theory.

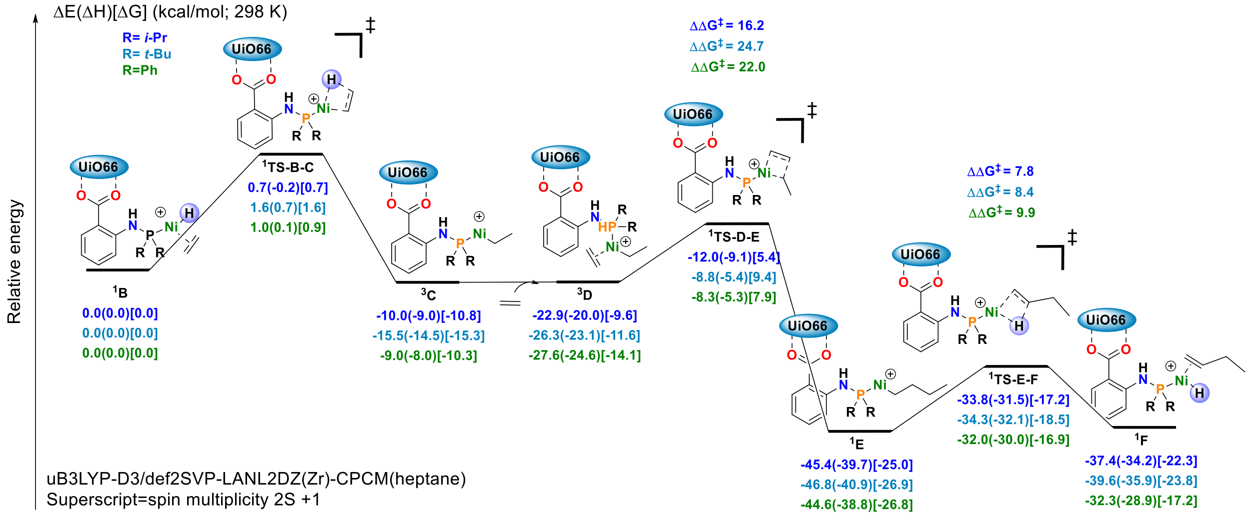

Figure S22. Calculated energetics for ethylene dimerization reactions using the L5, L7 and L9 (R=*i*-Pr, *t*-Bu, Ph) and truncated UiO-66. The energies shown were computed at the uB3LYP-D3/def2SVP-LANL2DZ-CPCM(heptane) level of theory.

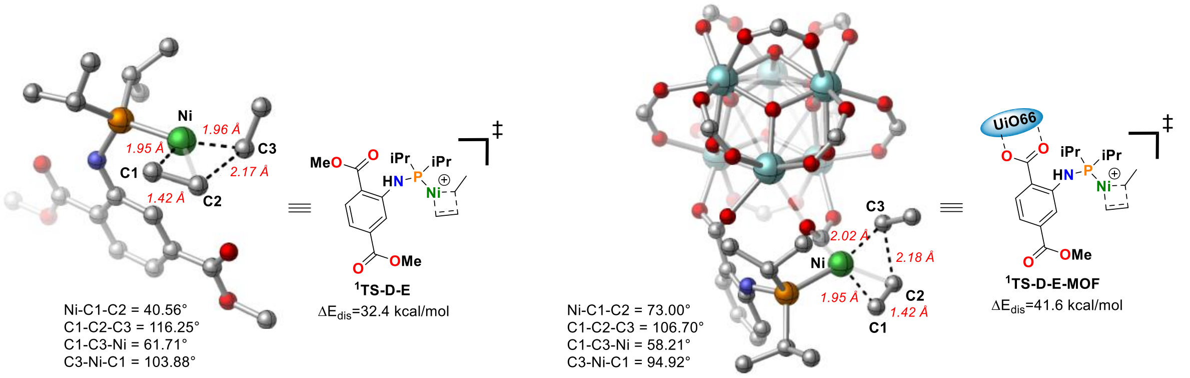

Figure S23. Comparison of the angle and distance for TS-D-E (migratory insertion) in a simple and UiO-66 system (distortion energy shown).

Table S1. SCXRD data and structure refinements of BDC-NHPiPr$_2$ (**L5**)and BDC-NHPPh$_2$ (**L8**).

Name	BDC-NHPiPr$_2$	BDC-NHPPh$_2$
**CCDC**	2267910	2267911
**Empirical formula**	$C_{16}H_{24}N_1O_4P_1$	$C_{22}H_{21}N_1O_4P_1$
**Formula weight**	325.33	394.39
**Temperature/K**	110	110
**Crystal system**	triclinic	triclinic
**Space group**	*P*-1	*P*-1
**a/Å**	6.2695(17)	8.577(2)
**b/Å**	7.478(2)	11.256(3)
**c/Å**	18.763(5)	11.906(3)
**α/°**	93.124(10)	66.454(5)
**β/°**	95.316(9)	73.037(6)
**γ/°**	97.770(9)	73.364(5)
**Volume/Å^3**	865.8(4)	988.8(4)
**Z**	2	2
**ρ_{calc}g/cm^3**	1.248	1.325
**μ/mm^{-1}**	1.553	1.469
**F(000)**	348.0	415.949
**Radiation**	Cu Kα (λ = 1.54184)	Cu Kα (λ = 1.54184)
**2Θ range for data collection/°**	9.49 to 134.234	9.74 to 144.34
**Index ranges**	$-7 \leq h \leq 7$, $-8 \leq k \leq 8$, $-22 \leq l \leq 22$	$-9 \leq h \leq 10$ $-12 \leq k \leq 13$, $0 \leq l \leq 14$

**Reflections collected**	2879	3790
**Independent reflections**	2879 [R_{sigma} = 0.0599]	3790 [R_{sigma} = 0.0762]
**Data/restraints/parameters**	2983/0/205	3860/0/337
**Goodness-of-fit on F^2**	1.146	1.053
**Final R indexes [I>=2σ (I)]**	R_1 = 0.0719, wR_2 = 0.2193	R_1 = 0.545, wR_2 = 0.1403
**Final R indexes [all data]**	R_1 = 0.0734, wR_2 = 0.2209	R_1 = 0.565, wR_2 = 0.1436
**Largest diff. peak/hole / e Å^{-3}**	0.709/-0.728	0.4197/-0.6091

$R_1 = \Sigma||F_o| - |F_c||/\Sigma|F_o|$. $wR_2 = |\Sigma w(|F_o|^2 - |F_c|^2)|/\Sigma|w(F_o^2)^2|^{1/2}$

Table S2. Calculation of molecular weight of UiO-66-**L4** to **L8** from the ^{31}P NMR

	mass dissolved in acid (mg)	integration MOF	integration standard	C standard (M)	volume (mL)	concentration MOF (M)	millimoles MOF	MW MOF
**UiO-66-NHPEt$_2$ (L4)**	2.7	1.00	3.94	0.0111	0.8	0.0028	0.0022	2396
**UiO-66-NHPiPr$_2$ (L5)**	3.1	3.11	9.01	0.0111	0.5	0.0038	0.0019	3236
**UiO-66-NHPtBu$_2$ (L6)**	3.1	1.12	2.88	0.0137	0.5	0.0053	0.0027	2327
**UiO-66-NHPcyclo$_2$ (L7)**	2.9	1.13	2.87	0.0111	0.5	0.0044	0.0022	2654
**UiO-66-NHPPh$_2$ (L8)**	2.6	0.99	3.01	0.0111	0.5	0.0037	0.0018	2849

Table S3. Ni to Zr ratio from the ICP-MS.

Sample Name	Ni Ratio	Zr Ratio	Result formula
**UiO-66-L4-Ni**	0.1409	1	UiO-66-L4-Ni0.8
**UiO-66-L5-Ni**	0.1387	1	UiO-66-L5-Ni0.8
**UiO-66-L6-Ni**	0.1437	1	UiO-66-L6-Ni0.9
**UiO-66-L7-Ni**	0.1372	1	UiO-66-L7-Ni0.8
**UiO-66-L8-Ni**	0.1401	1	UiO-66-L8-Ni0.8

Table S4. Reaction optimization for ethylene dimerization reaction of **UiO-66-L8-Ni**.

	MOF catalyst	Solvent	Activator	Temperature	TOF[a, b]	C4 selectivity
1	UiO-66-L8-Ni	Heptane	Et_2AlCl	25	22500	95
2	UiO-66-L8-Ni	Toluene	Et_2AlCl	25	7800	76
3	UiO-66-L8-Ni	Methylcyclohexane	Et_2AlCl	25	8115	83
4	UiO-66-L8-Ni	THF	Et_2AlCl	25	1000	70
5	UiO-66-L8-Ni	Heptane	MMAO	25	1800	90
6	UiO-66-L8-Ni	Toluene	MMAO	25	700	32
7	UiO-66-L8-Ni	Heptane	No Activator	25	N/A	-
8	UiO-66-L8-Ni	Heptane	Et_2AlCl	50	18500	92
9	UiO-66-L8-Ni	Heptane	Et_2AlCl	0	12700	97
10	UiO-66-L8-Ni	Heptane	Et_2AlCl (50 equiv.)	25	29700	95
11	UiO-66-L8-Ni	Heptane	Et_2AlCl (100 equiv.)	25	31100	94

a TOF shown in units of (mol ethylene)/(mol Ni·h). b Calculated from GC-FID peak integrations of the brominated products against heptane solvent. Condition: The MOF catalysts (6mg, 2 µmol) were preactivated with 40µl Et_2AlCl (20 equiv.) activator solution in 10ml Heptane for 1h and used to catalyze the reactions in stainless steel reactor at 15 bar ethylene. Reaction details are provided in S2.6, SI.

Table S5. Comparison of the turnover frequency，turnover number and selectivity for ethylene dimerization reaction of organometallic Ni complexes and heterogeneous Ni catalysts.

**Catalyst Name**	**TOF (mol ethylene/mol Ni/ h)**	**Reaction time (Min)**	**Temperature (°C)**	**Pressure (bar)**	**C4 Selectivity (%)**	**Reference**
N^N bidentate Ni	1.51e+6	30	30	5.5	83	10
P^N Ni	1.66e+5	60	r.t.	30	97.1	11
$NiBr_2$DMP-Xantphos	4.3e+4	30	20	30	97	12
Diphosphinated Calix[4]arene Ni	1.21e+6	15	25	20	95	13
Ni_2Cl_4(cyclo-tetraphos)	4.27e+4	30	20	14.3	~40	14
PSiP-Ni-C2	1.14e+5	30	45	10	94.7	15
PSiP-Ni-C3	2.75e+3	60	45	10	98.8	15
$NHPPh_2Ni$	2.93e+4	60	45	30	92.4	16
$PiPr_2NPPh_2Ni$	7.59e+4	60	45	30	71.7	16
Phillips	2.48e+4	60	55	48	97.7	17
Heterogeneous						
CFA-1	1.66e+4	60	22	25	95.1	18
MFU-4I	4.15e+4	60	25	50	97.4	19
NU-1000	6.6e+3	60	21	15	~94	20
UMOFN	4.55e+3	60	25	10	>71.4	21
IL-PON-RT	151	120	50	15	70	22
Ni-ZIF-8	1.12e+6	10	35	50	97	23
UiO-66-L5-Ni	2.9e+4	60	22	15	99	This work

Table S6. Data conclusion for gas phase reaction and recycle in solution phase.

MOF catalyst	fid heptane	fid dibromobutane	heptane/ mol	dibromobutane/ mol	Ni /mol	TOF (h-1)	selectivity
at 15 bar, Gas-phase							
UiO-66-L5-Ni	750811	63348	0.068	0.009925612	2.02881E-06	9800	99
UiO-66-L5-Ni	728244	66615	0.068	0.010760938	2.02881E-06	11000	99
UiO-66-L5-Ni	773277	84261	0.068	0.012818775	2.02881E-06	12000	99
Recovered catalyst in solution phase							
UiO-66-L5-Ni	566126	122976	0.068	0.025554199	2.02881E-06	25000	99

Distortion Energy Calculation

Single-point energy calculations were performed for fragments generated from **TS-D-E** at the same level of theory used for optimization: **ethane$_{\mathbf{fromTS\text{-}D\text{-}E}}$** (frag1) and **C$_{\mathbf{fromTS\text{-}D\text{-}E}}$** (frag2) (i.e. the remaining fragment after removing **Ethane** in **TS-D-E**; frag2). The contributions of distortions were then calculated as follows:

$$\Delta E\ (distortion) = [E\ (fragment\ 1) + E\ (fragment\ 2)] - [E\ (C_{opt}) + E(ethane_{opt})]$$

Table S7. Single-point energies are used for the distortion analysis.

Structure	*E (**Ethane**)*	*E (**C**)*	*E(frag1)*	*E(frag2)*	ΔE_{dist}
**TS-D-E**	-78.5329324635 a.u.	-2907.6368693 a.u.	-78.5107653932 a.u.	-2907.60732534 a.u.	32.4 kcal/mol
**TS-D-E-MOF**	-78.5329322054 a.u.	-5604.69519673 a.u.	-78.5028881751 a.u.	-5604.65900027 a.u.	41.6 Kcal/mol

References:

(1) Morris, W.; Briley, W. E.; Auyeung, E.; Cabezas, M. D.; Mirkin, C. A. Nucleic Acid–Metal Organic Framework (MOF) Nanoparticle Conjugates. *J. Am. Chem. Soc.* **2014,** *136* , 7261-7264.
(2) Fliedel, C.; Ghisolfi, A.; Braunstein, P. Functional Short-Bite Ligands: Synthesis, Coordination Chemistry, and Applications of N-Functionalized Bis(diaryl/dialkylphosphino)amine-type Ligands. *Chem. Rev.* **2016,** *116* (16), 9237-9304
(3) McFord, A. W., Butts, C. P., Fey, N., & Alder, R. W. 3× Axial vs 3× Equatorial: The Δ G GA Value Is a Robust Computational Measure of Substituent Steric Effects. *J. Am. Chem. Soc.* **2021**, *143*, 13573-13578.
(4) (a) Lee, C.; Yang, W.; Parr, R. G., Development of the Colle-Salvetti Correlation-Energy Formula into a Functional of the Electron Density. *Phys. Rev. B* **1988**, *37*, 785–789. (b) Becke, A. D., Density-Functional Thermochemistry. III. The Role of Exact Exchange. *J. Chem. Phys.* **1993**, *98*, 5648–5652.
(5) (a) Grimme, S. Accurate description of van der Waals complexes by density functional theory including empirical corrections. *J. Comput. Chem.* **2004**, *25*, 1463-1473. (b) Grimme, S.; Antony, J.; Ehrlich, S.; Krieg, H. A consistent and accurate ab initio parametrization of density functional dispersion correction (DFT-D) for the 94 elements H-Pu. *J. Chem. Phys.* **2010**, *132*, 154104. (c) Grimme, S. Density functional theory with London dispersion corrections. *WIRES Comput. Mol. Sci.* **2011**, *1*, 211-228. (d) Ehrlich, S.; Moellmann, J.; Grimme, S. Dispersion-Corrected Density Functional Theory for Aromatic Interactions in Complex Systems. *Acc. Chem. Res.* **2012**, *46*, 916-926.
(6) (a) Weigend, F.; Ahlrichs, R. Balanced basis sets of split valence, triple zeta valence and quadruple zeta valence quality for H to Rn: Design and assessment of accuracy. *Phys. Chem. Chem. Phys.* **2005**, *7*, 3297-3305. (b) Weigend, F. Accurate Coulomb-fitting basis sets for H to Rn. *Phys. Chem. Chem. Phys.* **2006**, *8*, 1057-1065.
(7) (a) Klamt, A.; Schüürmann, G. COSMO: a new approach to dielectric screening in solvents with explicit expressions for the screening energy and its gradient. *J. Chem. Soc. Perkin Trans. 2* **1993**, *0*, 799-805. (b) Tomasi, J.; Persico, M. Molecular Interactions in Solution: An Overview of Methods Based on Continuous Distributions of the Solvent. *Chem. Rev.* **1994**, *94*, 2027-2094. (c) Andzelm, J.; Kölmel, C.; Klamt, A. Incorporation of solvent effects into density functional calculations of molecular energies and geometries. *J. Chem. Phys.* **1995**, *103*, 9312-9320. (d) Barone, V.; Cossi, M. Quantum Calculation of Molecular Energies and Energy Gradients in Solution by a Conductor Solvent Model. *J. Phys. Chem. A* **1998**, *102*, 1995-2001. (e) Cossi, M.; Rega, N.; Scalmani, G.; Barone, V. Energies, structures, and electronic properties of molecules in solution with the C-PCM solvation model. *J. Comput. Chem.* **2003**, *24*, 669-681.
(8) Gaussian 16, Revision C.01, Frisch, M. J.; Trucks, G. W.; Schlegel, H. B.; Scuseria, G. E.; Robb, M. A.; Cheeseman, J. R.; Scalmani, G.; Barone, V.; Petersson, G. A.; Nakatsuji, H.; Li, X.; Caricato, M.; Marenich, A. V.; Bloino, J.; Janesko, B. G.; Gomperts, R.; Mennucci, B.; Hratchian, H. P.; Ortiz, J. V.; Izmaylov, A. F.; Sonnenberg, J. L.; Williams-Young, D.; Ding, F.; Lipparini, F.; Egidi, F.; Goings, J.; Peng, B.; Petrone, A.; Henderson, T.; Ranasinghe, D.; Zakrzewski, V. G.; Gao, J.; Rega, N.; Zheng, G.; Liang, W.; Hada, M.; Ehara, M.; Toyota, K.; Fukuda, R.; Hasegawa, J.; Ishida, M.; Nakajima, T.; Honda, Y.; Kitao, O.; Nakai, H.; Vreven, T.; Throssell, K.; Montgomery, J. A., Jr.; Peralta, J. E.; Ogliaro, F.; Bearpark, M. J.; Heyd, J. J.; Brothers, E. N.; Kudin, K. N.; Staroverov, V. N.; Keith, T. A.; Kobayashi, R.; Normand, J.; Raghavachari, K.; Rendell, A. P.; Burant, J. C.; Iyengar, S. S.; Tomasi, J.; Cossi, M.; Millam, J. M.; Klene, M.; Adamo, C.; Cammi, R.; Ochterski, J. W.; Martin, R. L.; Morokuma, K.; Farkas, O.; Foresman, J. B.; Fox, D. J. Gaussian, Inc., Wallingford CT, 2016.
(9) Legault, C. Y. (2009) CYLview, 1.0b, Université de Sherbrooke: Sherbrooke, Canada, http://www.cylview.org.
(10) Chandran, D.; Byeon, S. J.; Suh, H.; Kim, I. Effect of Ion-Pair Strength on Ethylene Oligomerization by Divalent Nickel Complexes. *Catal. Lett.* **2013,** *143* (7), 717-722, DOI: 10.1007/s10562-013-1021-7.
(11) Buchard, A.; Auffrant, A.; Klemps, C.; Vu-Do, L.; Boubekeur, L.; Goff, X. F. L.; Floch, P. L. Highly efficient P–N nickel(ii) complexes for the dimerisation of ethylene. *Chem. Commun.* **2007,** (15), 1502-1504, DOI: 10.1039/B618401D.
(12) Mora, G.; van Zutphen, S.; Klemps, C.; Ricard, L.; Jean, Y.; Le Floch, P. Synthesis, X-Ray, and Electronic Structures of a New Nickel Dibromide Complex. Activity in the Regioselective Catalyzed Dimerization of Ethylene into 1-Butene. *Inorg. Chem.* **2007,** *46* (24), 10365-10371, DOI: 10.1021/ic701529a.
(13) Lejeune, M.; Sémeril, D.; Jeunesse, C.; Matt, D.; Peruch, F.; Lutz, P. J.; Ricard, L. Diphosphines with Expandable Bite Angles: Highly Active Ethylene Dimerisation Catalysts Based on Upper Rim, Distally Diphosphinated Calix[4]arenes. *Chemistry – A European Journal* **2004,** *10* (21), 5354-5360, DOI: https://doi.org/10.1002/chem.200400492.
(14) Bianchini, C.; Gonsalvi, L.; Oberhauser, W.; Sémeril, D.; Brüggeller, P.; Gutmann, R. Schulz–Flory oligomerisation of ethylene by the binuclear nickel(ii) complex Ni2Cl4[cis,trans,cis-1,2,3,4-tetrakis-(diphenylphosphino)cyclobutane]. *Dalton Transactions* **2003,** (20), 3869-3875, DOI: 10.1039/B308388H.

(15) Meng, X.; Zhang, L.; Chen, Y.; Jiang, T. Silane-bridged diphosphine ligands for nickel-catalyzed ethylene oligomerization. *Reaction Kinetics, Mechanisms and Catalysis* **2016,** *119* (2), 481-490, DOI: 10.1007/s11144-016-1056-z.
(16) Boulens, P.; Lutz, M.; Jeanneau, E.; Olivier-Bourbigou, H.; Reek, J. N. H.; Breuil, P.-A. R. Iminobisphosphines to (Non-)Symmetrical Diphosphinoamine Ligands: Metal-Induced Synthesis of Diphosphorus Nickel Complexes and Application in Ethylene Oligomerisation Reactions. *Eur. J. Inorg. Chem.* **2014,** *2014* (23), 3754-3762, DOI: https://doi.org/10.1002/ejic.201402430.
(17) Carter, C. O., Surface conditioning in olefin dimerization reactors. 1985.
(18) Metzger, E. D.; Comito, R. J.; Wu, Z.; Zhang, G.; Dubey, R. C.; Xu, W.; Miller, J. T.; Dincă, M. Highly Selective Heterogeneous Ethylene Dimerization with a Scalable and Chemically Robust MOF Catalyst. *ACS Sustainable Chemistry & Engineering* **2019,** *7* (7), 6654-6661, DOI: 10.1021/acssuschemeng.8b05703.
(19) Metzger, E. D.; Brozek, C. K.; Comito, R. J.; Dincă, M. Selective Dimerization of Ethylene to 1-Butene with a Porous Catalyst. *ACS Central Science* **2016,** *2* (3), 148-153, DOI: 10.1021/acscentsci.6b00012.
(20) Madrahimov, S. T.; Gallagher, J. R.; Zhang, G.; Meinhart, Z.; Garibay, S. J.; Delferro, M.; Miller, J. T.; Farha, O. K.; Hupp, J. T.; Nguyen, S. T. Gas-Phase Dimerization of Ethylene under Mild Conditions Catalyzed by MOF Materials Containing (bpy)NiII Complexes. *ACS Catalysis* **2015,** *5* (11), 6713-6718, DOI: 10.1021/acscatal.5b01604.
(21) Hu, Y.; Zhang, Y.; Han, Y.; Sheng, D.; Shan, D.; Liu, X.; Cheng, A. Ultrathin Nickel-Based Metal–Organic Framework Nanosheets as Reusable Heterogeneous Catalyst for Ethylene Dimerization. *ACS Applied Nano Materials* **2019,** *2* (1), 136-142, DOI: 10.1021/acsanm.8b01762.
(22) Rozhko, E.; Bavykina, A.; Osadchii, D.; Makkee, M.; Gascon, J. Covalent organic frameworks as supports for a molecular Ni based ethylene oligomerization catalyst for the synthesis of long chain olefins. *J. Catal.* **2017,** *345*, 270-280, DOI: https://doi.org/10.1016/j.jcat.2016.11.030.
(23) Chen, C.; Alalouni, M. R.; Dong, X.; Cao, Z.; Cheng, Q.; Zheng, L.; Meng, L.; Guan, C.; Liu, L.; Abou-Hamad, E.; Wang, J.; Shi, Z.; Huang, K. W.; Cavallo, L.; Han, Y. Highly Active Heterogeneous Catalyst for Ethylene Dimerization Prepared by Selectively Doping Ni on the Surface of a Zeolitic Imidazolate Framework. *J. Am. Chem. Soc.* **2021,** *143* (18), 7144-7153, DOI: 10.1021/jacs.1c02272.

Cartesian coordinates (xyz format) of all the structures involved in the reaction mechanism for the truncated UiO66 ligand (**Figure S21**) calculated at the uB3LYP-d3/def2-SVP-LANL2DZ(Zr)
The rest cartesian coordinates are accessible for request to the authors.

[1]B-UiO66 (*i*-Pr)

$E(scf)_{gas}$ = -5604.63727836 a.u.
$E(scf)_{CPCM(heptane)}$ = -5604.68417088 a.u.
ν_{min} = 17.79 cm^{-1}

Ni	5.683694	0.563052	0.811511	H	7.101542	-1.266997	2.029568
P	4.344770	2.274299	0.390214	H	-0.396664	4.498733	-1.074458
N	2.896813	1.463701	-0.045601	C	1.013352	0.165094	1.794931
H	2.925152	0.502678	0.298815	O	0.239629	-0.116787	2.748421
C	1.588041	1.964732	0.076207	O	2.095902	-0.485569	1.547937
C	0.649606	1.322533	0.930725	Zr	0.354714	-1.435464	4.541330
C	1.188700	3.112960	-0.628789	Zr	3.194812	-2.159266	2.540082
C	-0.656313	1.840005	1.031506	Zr	3.643532	-1.231863	5.950523
C	-0.107886	3.610137	-0.507776	O	0.632936	0.656542	5.315248
H	1.900152	3.602258	-1.294966	Zr	1.003871	-4.792544	3.493294
C	-1.040975	2.969805	0.317625	Zr	1.436070	-3.861921	6.927934
H	-1.358992	1.336227	1.696856	O	0.629607	-3.434706	5.043262
H	-2.058955	3.354362	0.403863	O	-1.508532	-2.203133	3.574177
C	3.814802	3.481965	1.722502	O	0.991652	-2.640402	2.702137
H	2.983169	4.010255	1.225472	O	2.440499	-1.247894	4.229427
C	4.810311	3.339085	-1.070492	O	-1.169808	-1.473206	6.155179
C	6.268729	3.818147	-1.001431	O	1.473539	-1.590629	6.526581
H	6.524461	4.321933	-0.059646	Zr	4.296397	-4.581116	4.898053
H	6.963922	2.972922	-1.128019	O	4.668028	-2.377298	4.247195
H	6.459852	4.528285	-1.821028	O	2.963812	-4.018392	3.374321
C	3.253375	2.769183	2.965288	O	4.777517	-0.191664	2.500865
H	2.495519	3.406999	3.445242	O	5.091713	-3.015684	1.705742
H	2.780122	1.805972	2.740526	O	2.612193	-3.181819	0.647852
H	4.035469	2.590336	3.715509	O	5.708510	-1.744915	6.638484
C	4.879603	4.516922	2.104591	O	2.716590	0.783823	6.174142
H	4.497155	5.142761	2.926604	O	4.933524	0.209341	4.721965
H	5.807019	4.039307	2.462256	O	3.540785	-1.188891	8.164720
H	5.133984	5.191081	1.275393	O	3.312729	-3.264008	6.184721
C	4.553806	2.580832	-2.379407	C	1.599391	1.266755	5.840445
H	4.851892	3.207387	-3.234625	O	2.201676	-5.355753	5.360426
H	5.155121	1.657231	-2.416897	O	-0.271902	-6.144695	4.711988
H	3.500374	2.296964	-2.505286	O	2.041609	-6.715567	3.060175
H	4.142920	4.217261	-1.014674	O	-1.084798	-4.312479	2.900576
H	6.291885	1.195034	-0.299701	O	1.238498	-4.865886	1.254064
C	6.704165	-1.094474	-0.063658	O	2.654860	-5.391322	7.999264
C	7.335857	-0.743482	1.099280	O	-0.001929	-5.558798	6.871643
H	5.938112	-1.876984	-0.058118	O	-0.497082	-3.016885	7.653571
H	7.085884	-0.775454	-1.036693	O	2.157892	-2.855625	8.787903
H	8.233406	-0.115484	1.095822	C	-1.833263	-3.313259	3.074287

H	0.460517	-2.485409	1.909686	H	2.151896	-6.286452	5.617848
C	-1.312085	-2.174392	7.194867	C	-0.528724	-6.228447	5.943568
H	1.130826	-1.037371	7.241590	C	3.201606	-7.152903	3.283451
O	5.768449	-4.583241	3.179271	C	3.756078	-5.954008	7.763467
O	6.123263	-3.850643	5.949455	H	-2.886100	-3.418973	2.739288
O	4.455492	-5.838853	6.719639	H	-2.248473	-2.022083	7.770511
O	4.112862	-6.579392	3.939996	H	5.978414	1.214997	3.346544
H	5.603461	-2.193454	4.097340	H	6.646933	-4.266908	1.394386
C	5.228487	0.416923	3.539141	H	1.857479	-4.544574	-0.637382
C	5.836354	-3.966534	2.092219	H	7.370752	-2.782709	7.121960
C	1.902851	-4.205246	0.418685	H	3.173866	-1.691048	10.085148
C	6.403175	-2.794811	6.578021	H	-1.289849	-6.978752	6.241920
C	2.960651	-1.915334	9.019494	H	3.444451	-8.152950	2.868123
H	1.453012	2.349394	6.040311	H	4.148544	-6.631062	8.550179

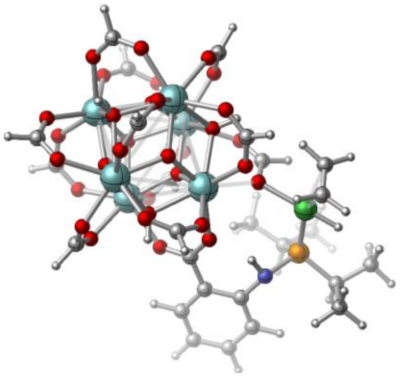

Zero-point correction= 0.720335 (Hartree/Particle)
Thermal correction to Energy= 0.795726
Thermal correction to Enthalpy= 0.796670
Thermal correction to Gibbs Free Energy= 0.620254
Sum of electronic and zero-point Energies= -5603.916944
Sum of electronic and thermal Energies= -5603.841553
Sum of electronic and thermal Enthalpies= -5603.840608
Sum of electronic and thermal Free Energies= -5604.017024

^{3}B-UiO66 (*i*-Pr)

E(scf)$_{gas}$ = -5604.61570674 a.u.
E(scf)$_{CPCM(heptane)}$ = -5604.66463684 a.u.
ν_{min} = 15.10 cm^{-1}

Ni	5.477904	0.431645	0.815026	C	-0.208201	3.913005	-0.111678
P	4.232088	2.278462	0.250937	H	1.734209	3.829205	-1.052794
N	2.688373	1.594235	0.044398	C	-1.112306	3.284297	0.754650
H	2.709625	0.615062	0.335751	H	-1.424082	1.604721	2.078431
C	1.432101	2.160068	0.281563	H	-2.098969	3.716297	0.931924
C	0.524835	1.524325	1.174735	C	4.005199	3.771187	1.343771
C	1.046291	3.355096	-0.349565	H	3.254675	4.387371	0.820521
C	-0.741573	2.103019	1.388620	C	4.820840	2.928028	-1.401669

C	6.353592	2.827830	-1.463834
H	6.855412	3.343120	-0.630853
H	6.669002	1.769057	-1.452207
H	6.729381	3.266721	-2.401172
C	3.438924	3.381547	2.716961
H	3.094784	4.286181	3.242009
H	2.584728	2.693676	2.649893
H	4.206095	2.916751	3.352336
C	5.301818	4.581075	1.473273
H	5.138233	5.432268	2.152772
H	6.118156	3.974006	1.900737
H	5.644881	4.992519	0.512756
C	4.166319	2.159494	-2.555603
H	4.555025	2.533717	-3.515973
H	4.393765	1.083274	-2.490452
H	3.072885	2.266483	-2.559006
H	4.521644	3.990069	-1.450998
H	4.680413	-0.360892	-0.238744
C	7.142674	-1.015135	0.249535
C	7.589926	-0.318461	1.327711
H	6.602756	-1.958033	0.356048
H	7.389758	-0.699739	-0.769810
H	8.228352	0.564903	1.217714
H	7.449660	-0.703613	2.342543
H	-0.489795	4.838720	-0.619595
C	0.884181	0.306406	1.957704
O	0.107820	-0.025379	2.895614
O	1.950174	-0.346033	1.662813
Zr	0.224619	-1.416996	4.612595
Zr	3.078739	-2.061733	2.581518
Zr	3.501683	-1.244263	6.031889
O	0.486948	0.647935	5.477899
Zr	0.894548	-4.732256	3.453991
Zr	1.305007	-3.916590	6.920659
O	0.505668	-3.431685	5.047035
O	-1.630881	-2.161818	3.612445
O	0.875056	-2.555229	2.731589
O	2.310154	-1.215277	4.307985
O	-1.309096	-1.517010	6.218480
O	1.335145	-1.636148	6.594654
Zr	4.175444	-4.553739	4.884327
O	4.541240	-2.328362	4.299469
O	2.852627	-3.947793	3.367652
O	4.623963	-0.153126	2.607810
O	5.014441	-2.910264	1.746583

O	2.507774	-3.032343	0.671421
O	5.574115	-1.742832	6.696084
O	2.568592	0.749935	6.343189
O	4.763626	0.278517	4.823326
O	3.400815	-1.283413	8.249775
O	3.183587	-3.283349	6.207680
C	1.447524	1.240611	6.031275
O	2.084877	-5.352275	5.309447
O	-0.381567	-6.131488	4.622015
O	1.937746	-6.638509	2.965283
O	-1.194264	-4.244679	2.868180
O	1.134883	-4.737900	1.214634
O	2.526554	-5.475599	7.947419
O	-0.124492	-5.617336	6.801655
O	-0.633520	-3.105788	7.667615
O	2.016303	-2.969610	8.815481
C	-1.947905	-3.255249	3.072846
H	0.346101	-2.371586	1.943705
C	-1.451348	-2.252618	7.233526
H	0.988086	-1.108195	7.326436
O	5.661257	-4.512902	3.199478
O	5.993554	-3.838382	5.977336
O	4.336676	-5.870166	6.663845
O	4.002129	-6.527589	3.864662
H	5.476113	-2.139490	4.153435
C	5.052918	0.482256	3.641501
C	5.740032	-3.876296	2.123295
C	1.801555	-4.048698	0.404897
C	6.269344	-2.793010	6.622536
C	2.819670	-2.037880	9.078600
H	1.293838	2.311587	6.282304
H	2.038805	-6.291364	5.534931
C	-0.643975	-6.257206	5.848533
C	3.096882	-7.080990	3.183834
C	3.632264	-6.023769	7.699285
H	-2.998727	-3.353994	2.729318
H	-2.390540	-2.124633	7.810649
H	5.782216	1.295555	3.429778
H	6.554019	-4.180808	1.429541
H	1.762560	-4.351913	-0.662266
H	7.234246	-2.787577	7.171677
H	3.032176	-1.850140	10.151575
H	-1.403700	-7.019902	6.117937
H	3.343844	-8.067416	2.739065
H	4.024465	-6.724306	8.465378

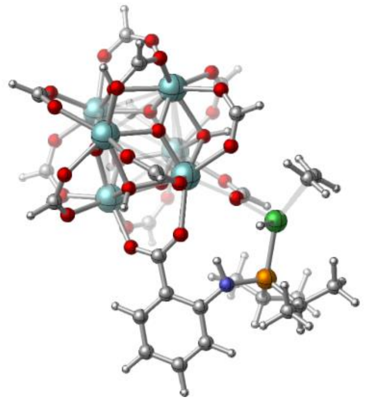

Zero-point correction= 0.716956 (Hartree/Particle)
Thermal correction to Energy= 0.793232
Thermal correction to Enthalpy= 0.794176
Thermal correction to Gibbs Free Energy= 0.613545
Sum of electronic and zero-point Energies= -5603.898750
Sum of electronic and thermal Energies= -5603.822475
Sum of electronic and thermal Enthalpies= -5603.821531
Sum of electronic and thermal Free Energies= -5604.002162

[1]TS-B-C-UiO66 (*i*-Pr)

$E(scf)_{gas}$ = -5604.63586668 a.u.

$E(scf)_{CPCM(heptane)}$ = -5604.68304446 a.u.

ν_{min} = -106.16 cm^{-1}

Ni	5.613612	0.500441	0.930862
P	4.271497	2.127597	0.294977
N	2.778229	1.399702	-0.137438
H	2.787012	0.436401	0.195837
C	1.500372	1.955285	0.063908
C	0.595510	1.379578	0.999128
C	1.104635	3.098515	-0.649905
C	-0.680720	1.952589	1.162396
C	-0.159283	3.655583	-0.461899
H	1.795794	3.533945	-1.373781
C	-1.062916	3.077570	0.439327
H	-1.360039	1.497637	1.884770
H	-2.056975	3.507229	0.576983
C	3.812973	3.434881	1.558127
H	3.007835	3.981344	1.038723
C	4.841296	3.101346	-1.193488
C	6.346526	3.402609	-1.118100
H	6.647526	3.909541	-0.191146
H	6.932029	2.472792	-1.192426
H	6.639735	4.047861	-1.960748
C	3.229158	2.829142	2.848592
H	2.454065	3.495307	3.256480
H	2.778809	1.841151	2.703019
H	4.000321	2.727681	3.624850
C	4.937049	4.431069	1.868524
H	4.593715	5.132100	2.645925
H	5.836376	3.925984	2.259215
H	5.228264	5.032242	0.996202
C	4.500405	2.360000	-2.491819
H	4.855353	2.944469	-3.355297
H	4.999737	1.377038	-2.530333
H	3.421928	2.187240	-2.607089
H	4.279990	4.052166	-1.160504
H	5.990746	0.701884	-0.435763
C	7.242975	-0.560492	0.062850
C	7.304164	-0.571190	1.443488
H	6.796314	-1.404672	-0.475087
H	7.918467	0.067143	-0.524826
H	7.976137	0.114531	1.973732
H	6.926602	-1.424967	2.004098
H	-0.447664	4.540038	-1.035230
C	0.970536	0.238492	1.880281
O	0.210725	-0.041964	2.843912
O	2.059771	-0.403759	1.632889

Zr	0.332347	-1.421996	4.608850	O	-0.488778	-3.111856	7.676886
Zr	3.163279	-2.068908	2.575319	O	2.171490	-2.964268	8.802016
Zr	3.624579	-1.234551	6.013215	C	-1.854001	-3.266464	3.101715
O	0.584662	0.639939	5.444180	H	0.428620	-2.397829	1.949286
Zr	0.994425	-4.744878	3.459635	C	-1.314376	-2.264380	7.246893
Zr	1.447957	-3.915401	6.914102	H	1.117985	-1.108514	7.320002
O	0.623698	-3.435786	5.050197	O	5.736362	-4.479136	3.084949
O	-1.530136	-2.166872	3.625563	O	6.132897	-3.837704	5.886144
O	0.963716	-2.569799	2.735467	O	4.485123	-5.855144	6.623361
O	2.414205	-1.209654	4.296619	O	4.126442	-6.516143	3.830558
O	-1.184028	-1.530273	6.228747	H	5.584148	-2.114116	4.100811
O	1.461648	-1.635065	6.585537	C	5.218009	0.548790	3.728388
Zr	4.300657	-4.544619	4.841617	C	5.789713	-3.833206	2.014591
O	4.651827	-2.308822	4.256973	C	1.862195	-4.064644	0.400934
O	2.950388	-3.950279	3.347242	C	6.405474	-2.806331	6.559237
O	4.776807	-0.011702	2.663121	C	2.967057	-2.024891	9.060128
O	5.058133	-2.853113	1.673568	H	1.381751	2.315249	6.233562
O	2.567183	-3.045451	0.653874	H	2.175532	-6.290941	5.526764
O	5.698874	-1.770442	6.673030	C	-0.504426	-6.269133	5.874198
O	2.662130	0.759717	6.317978	C	3.215145	-7.078022	3.163635
O	4.899024	0.280540	4.894038	C	3.794250	-6.009891	7.667848
O	3.536252	-1.266514	8.226016	H	-2.908468	-3.367777	2.770540
O	3.315560	-3.278941	6.175336	H	-2.249957	-2.139753	7.830532
C	1.542592	1.242280	5.996258	H	5.972343	1.349988	3.583356
O	2.214316	-5.352484	5.297130	H	6.576514	-4.129967	1.288758
O	-0.259137	-6.144605	4.643475	H	1.809453	-4.374950	-0.663742
O	2.049966	-6.644804	2.961618	H	7.378412	-2.807182	7.093707
O	-1.103285	-4.258571	2.900317	H	3.183918	-1.832880	10.131284
O	1.206801	-4.752891	1.222477	H	-1.256021	-7.035516	6.155216
O	2.689105	-5.466475	7.928629	H	3.463956	-8.063172	2.717346
O	0.024511	-5.623736	6.817758	H	4.199422	-6.706866	8.430388

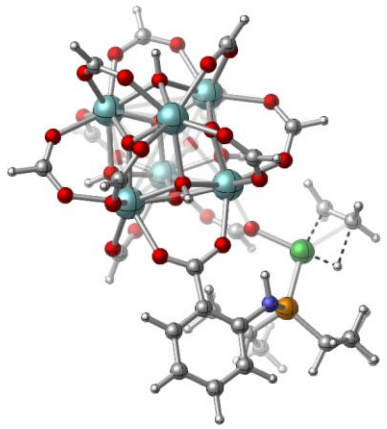

Zero-point correction= 0.719579 (Hartree/Particle)

Thermal correction to Energy= 0.794308
Thermal correction to Enthalpy= 0.795252
Thermal correction to Gibbs Free Energy= 0.620234
Sum of electronic and zero-point Energies= -5603.916288
Sum of electronic and thermal Energies= -5603.841559
Sum of electronic and thermal Enthalpies= -5603.840615
Sum of electronic and thermal Free Energies= -5604.015633

^{3}TS-B-C-UiO66 (*i*-Pr)
$E(scf)_{gas}$ = -5604.60870184 a.u.
$E(scf)_{CPCM(heptane)}$ = -5604.65683632 a.u.
ν_{min} = -734.39 cm^{-1}

Ni	5.623645	0.571721	0.853310	C	6.712577	-1.123541	-0.049874
P	4.275253	2.340081	0.298070	C	7.440041	-0.576316	1.016465
N	2.764959	1.586482	0.073708	H	6.153446	-2.050819	0.087945
H	2.796845	0.622466	0.411358	H	7.001799	-0.884029	-1.078255
C	1.485676	2.125745	0.254036	H	8.279222	0.102042	0.829973
C	0.561845	1.484579	1.125567	H	7.400903	-1.052285	2.000820
C	1.095894	3.302063	-0.408945	H	-0.462415	4.745520	-0.756963
C	-0.722909	2.038531	1.288047	C	0.919166	0.284494	1.935842
C	-0.178748	3.834346	-0.224543	O	0.137810	-0.034232	2.872791
H	1.797748	3.784719	-1.092058	O	1.991789	-0.367348	1.659324
C	-1.098249	3.199853	0.620836	Zr	0.237953	-1.411013	4.610702
H	-1.416810	1.536545	1.963774	Zr	3.098964	-2.080946	2.606127
H	-2.099655	3.612359	0.756997	Zr	3.511662	-1.238596	6.045470
C	3.971834	3.788141	1.429731	O	0.505155	0.659408	5.455556
H	3.180546	4.368887	0.926549	Zr	0.902690	-4.737130	3.479897
C	4.832435	3.053291	-1.337533	Zr	1.300662	-3.897061	6.941983
C	6.369303	3.061183	-1.381673	O	0.510255	-3.423219	5.061093
H	6.824579	3.614439	-0.546890	O	-1.614976	-2.155938	3.605492
H	6.759688	2.027475	-1.358277	O	0.893423	-2.564702	2.741786
H	6.723653	3.519862	-2.317920	O	2.324643	-1.219118	4.317256
C	3.438486	3.321533	2.792226	O	-1.302963	-1.494265	6.209185
H	3.027259	4.182844	3.341196	O	1.338837	-1.618776	6.599634
H	2.638985	2.572325	2.705579	Zr	4.179341	-4.559644	4.924040
H	4.240300	2.899401	3.414726	O	4.554425	-2.342658	4.326188
C	5.214432	4.675154	1.577139	O	2.862368	-3.961191	3.398903
H	5.000524	5.493112	2.283003	O	4.652261	-0.176120	2.603941
H	6.071537	4.110119	1.981283	O	5.016188	-2.943679	1.776690
H	5.521760	5.136843	0.627471	O	2.533438	-3.053242	0.688558
C	4.249416	2.250019	-2.506195	O	5.575840	-1.744458	6.725991
H	4.624496	2.657313	-3.458376	O	2.581895	0.761799	6.333189
H	4.552827	1.191963	-2.446510	O	4.781012	0.254164	4.823143
H	3.151372	2.277413	-2.525894	O	3.396748	-1.259683	8.262412
H	4.458785	4.091590	-1.380992	O	3.183603	-3.276163	6.232763
H	5.156789	-0.362299	-0.333973	C	1.465267	1.253700	6.008896

O	2.082469	-5.348348	5.344842
O	-0.383682	-6.122857	4.651279
O	1.942941	-6.648854	3.007625
O	-1.181574	-4.245839	2.878992
O	1.157024	-4.754263	1.239331
O	2.511530	-5.452794	7.985803
O	-0.134848	-5.593872	6.828326
O	-0.639651	-3.074417	7.673380
O	2.005508	-2.938635	8.833084
C	-1.932983	-3.252537	3.072707
H	0.368585	-2.386964	1.949891
C	-1.452452	-2.221896	7.229197
H	0.990261	-1.084691	7.326238
O	5.676473	-4.527718	3.241727
O	5.992742	-3.844862	6.020811
O	4.325248	-5.864694	6.712783
O	4.004727	-6.536729	3.913225
H	5.490123	-2.153344	4.183840
C	5.086564	0.446935	3.642150

C	5.758238	-3.893633	2.164935
C	1.826528	-4.071103	0.427268
C	6.269636	-2.795921	6.660155
C	2.809582	-2.006749	9.093701
H	1.314366	2.327973	6.247222
H	2.031484	-6.285443	5.577490
C	-0.652165	-6.238804	5.877619
C	3.100537	-7.092279	3.232658
C	3.615902	-6.007552	7.746426
H	-2.982510	-3.350498	2.725000
H	-2.393981	-2.086294	7.800726
H	5.838843	1.242665	3.441402
H	6.584936	-4.181896	1.479958
H	1.789822	-4.381577	-0.638010
H	7.233377	-2.789059	7.211054
H	3.016777	-1.811568	10.166356
H	-1.415809	-6.996910	6.148847
H	3.347065	-8.081733	2.794465
H	4.001890	-6.704045	8.519300

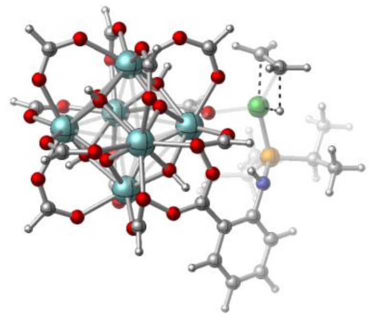

Zero-point correction= 0.718142 (Hartree/Particle)
Thermal correction to Energy= 0.793418
Thermal correction to Enthalpy= 0.794362
Thermal correction to Gibbs Free Energy= 0.616549
Sum of electronic and zero-point Energies= -5603.890560
Sum of electronic and thermal Energies= -5603.815284
Sum of electronic and thermal Enthalpies= -5603.814340
Sum of electronic and thermal Free Energies= -5603.992152

^{1}C-UiO66 (*i*-Pr)

$E(scf)_{gas}$ = -5604.64584985 a.u.
$E(scf)_{CPCM(heptane)}$ = -5604.69368998 a.u.
ν_{min} = 12.60 cm^{-1}

Ni	5.721228	0.532003	0.956638
P	4.325511	2.229062	0.361178
N	2.868565	1.417526	-0.047450
H	2.902995	0.465739	0.318113

C	1.563490	1.922720	0.075689
C	0.632172	1.307808	0.958224
C	1.159758	3.054033	-0.654351
C	-0.670290	1.834143	1.061483

C	-0.132713	3.560518	-0.530614
H	1.867182	3.522652	-1.339927
C	-1.058527	2.946664	0.323120
H	-1.367187	1.350779	1.747777
H	-2.073551	3.338438	0.411715
C	3.801066	3.512089	1.619046
H	2.948685	4.009603	1.126629
C	4.812965	3.209191	-1.156010
C	6.288077	3.640131	-1.102341
H	6.568051	4.141913	-0.166107
H	6.959032	2.776328	-1.232010
H	6.501547	4.336645	-1.928117
C	3.292183	2.852869	2.912828
H	2.589209	3.529591	3.422483
H	2.773279	1.901040	2.742542
H	4.115116	2.663672	3.616327
C	4.867637	4.572425	1.917574
H	4.500495	5.244982	2.709197
H	5.804719	4.119893	2.283604
H	5.101331	5.197407	1.044316
C	4.533391	2.397420	-2.427462
H	4.854173	2.968432	-3.313090
H	5.097906	1.449165	-2.423158
H	3.470586	2.145383	-2.541113
H	4.179228	4.113666	-1.153276
H	6.570789	0.903640	-0.335063
C	7.441191	0.063836	-0.171653
C	7.173849	-0.724471	1.027580
H	7.355910	-0.460930	-1.133717
H	8.320740	0.722402	-0.148533
H	7.827396	-0.578175	1.898608
H	6.817588	-1.750005	0.899340
H	-0.424694	4.435459	-1.116631
C	0.998930	0.167699	1.842498
O	0.229824	-0.102127	2.802399
O	2.081541	-0.487793	1.600253
Zr	0.336278	-1.435036	4.593059
Zr	3.145367	-2.181118	2.557928
Zr	3.637843	-1.267581	5.972586
O	0.628496	0.644552	5.377328
Zr	0.936179	-4.794791	3.520917
Zr	1.421725	-3.888199	6.952235
O	0.595450	-3.441537	5.080837
O	-1.544505	-2.172678	3.637331
O	0.939254	-2.635490	2.741756

O	2.419433	-1.266868	4.263569
O	-1.174074	-1.476389	6.222655
O	1.471762	-1.616754	6.567642
Zr	4.252687	-4.616413	4.884270
O	4.637834	-2.397608	4.236853
O	2.903251	-4.036701	3.381060
O	4.823047	-0.115939	2.572383
O	5.013426	-3.039129	1.681108
O	2.516765	-3.185452	0.666633
O	5.705107	-1.814919	6.636651
O	2.714966	0.751496	6.231086
O	4.930084	0.191550	4.803501
O	3.559840	-1.241965	8.187701
O	3.295519	-3.300554	6.188886
C	1.600704	1.244328	5.905780
O	2.154693	-5.377475	5.368517
O	-0.335525	-6.142688	4.745735
O	1.954649	-6.724000	3.066144
O	-1.154926	-4.286980	2.958335
O	1.137895	-4.861147	1.282740
O	2.641425	-5.434489	7.999661
O	-0.031107	-5.574072	6.905340
O	-0.497089	-3.034017	7.704999
O	2.171314	-2.903124	8.812196
C	-1.888572	-3.278443	3.140465
H	0.401668	-2.470584	1.955657
C	-1.310784	-2.183253	7.258963
H	1.140781	-1.066517	7.290446
O	5.689280	-4.620587	3.142001
O	6.097547	-3.911885	5.909518
O	4.423369	-5.887415	6.695798
O	4.037708	-6.609987	3.921455
H	5.570946	-2.222813	4.059943
C	5.268058	0.442610	3.640838
C	5.747101	-4.003260	2.053830
C	1.796430	-4.200612	0.441392
C	6.392839	-2.866282	6.549024
C	2.983146	-1.970391	9.042839
H	1.460176	2.325742	6.116431
H	2.101208	-6.309169	5.621454
C	-0.576053	-6.232894	5.980328
C	3.113592	-7.172024	3.273148
C	3.735283	-6.003992	7.746963
H	-2.946195	-3.370447	2.816611
H	-2.240686	-2.028757	7.844583

H	6.041751	1.226881	3.503916	H	3.208596	-1.755729	10.107962
H	6.539314	-4.319637	1.342176	H	-1.339176	-6.979118	6.283772
H	1.732243	-4.533651	-0.615717	H	3.342838	-8.171955	2.849768
H	7.369215	-2.868047	7.077256	H	4.131805	-6.689321	8.524481

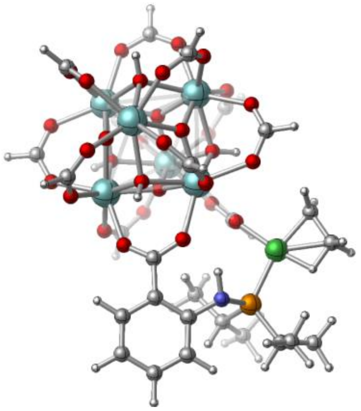

Zero-point correction= 0.722161 (Hartree/Particle)
Thermal correction to Energy= 0.797250
Thermal correction to Enthalpy= 0.798194
Thermal correction to Gibbs Free Energy= 0.621765
Sum of electronic and zero-point Energies= -5603.923689
Sum of electronic and thermal Energies= -5603.848600
Sum of electronic and thermal Enthalpies= -5603.847656
Sum of electronic and thermal Free Energies= -5604.024085

^{3}C-UiO66 (*i*-Pr)
$E(scf)_{gas}$ = -5604.65070374 a.u.
$E(scf)_{CPCM(heptane)}$ = -5604.70007063 a.u.
ν_{min} = 16.38 cm^{-1}

Ni	5.341602	0.045783	0.950503	C	5.865523	1.491743	-2.338839
P	4.136374	1.774335	-0.169691	H	6.624186	2.061030	-1.783417
N	2.441830	1.527837	0.022881	H	6.011695	0.424106	-2.103827
H	2.300340	0.584548	0.379029	H	6.066909	1.625675	-3.413112
C	1.433665	2.368201	0.523744	C	4.277526	3.432156	2.072407
C	0.626355	1.980350	1.643704	H	4.344332	4.455832	2.472229
C	1.159987	3.598797	-0.098770	H	3.296335	3.024137	2.356101
C	-0.413188	2.826475	2.073425	H	5.056521	2.829985	2.560760
C	0.141545	4.429859	0.364641	C	5.897829	3.946189	0.188870
H	1.735410	3.880410	-0.982814	H	6.082499	4.914495	0.680090
C	-0.655425	4.046498	1.451601	H	6.674860	3.245871	0.533989
H	-1.017738	2.500053	2.921774	H	6.024856	4.100430	-0.892248
H	-1.462003	4.693423	1.801489	C	3.409728	1.085943	-2.795176
C	4.491347	3.452556	0.553649	H	3.625093	1.159189	-3.872934
H	3.752159	4.132633	0.100778	H	3.468242	0.019643	-2.516711
C	4.429329	1.921830	-2.011317	H	2.377844	1.420680	-2.622946

H	4.293959	2.988063	-2.266444
H	8.031868	0.368925	-0.834332
C	8.130857	-0.272017	0.054174
C	7.314577	0.250300	1.228098
H	7.827953	-1.290617	-0.248234
H	9.210514	-0.324804	0.290472
H	7.562343	1.301163	1.459201
H	7.500292	-0.347232	2.141248
H	-0.048105	5.378330	-0.144072
C	0.886258	0.735883	2.413941
O	1.878978	0.025971	2.014700
O	0.198430	0.424305	3.422571
Zr	3.066368	-1.631731	2.664717
Zr	0.392530	-1.318719	4.923417
Zr	0.952021	-4.401864	3.113229
O	2.337033	-2.132256	0.583836
Zr	3.755164	-1.445692	6.142910
Zr	4.328785	-4.474629	4.316540
O	4.076961	-2.391076	4.291827
O	4.609512	0.116675	2.893409
O	2.490468	-0.461322	4.510512
O	1.277721	-2.340937	3.316228
O	5.057883	-2.034555	1.545293
O	3.127211	-3.852233	2.472471
Zr	1.595957	-4.244796	6.625161
O	0.051623	-3.596835	5.064730
O	1.822915	-2.205268	6.172067
O	-1.446793	-1.763670	3.773141
O	-0.955693	-1.678232	6.636883
O	0.691595	0.437600	6.274128
O	-0.152077	-6.073638	4.045663
O	0.950294	-3.880164	0.921480
O	-1.121114	-3.704013	2.666386
O	1.931136	-6.241545	2.307942
O	2.290730	-4.688007	4.689716
C	1.490380	-2.996787	0.210849
O	3.822157	-3.706704	6.411396
O	5.895855	-2.009462	6.432752
O	3.688624	-1.838804	8.322486
O	5.023520	0.189400	5.105456
O	2.820637	0.373105	7.015622
O	4.501947	-6.192300	5.705170
O	6.252134	-3.913694	5.276438
O	5.791928	-3.900395	2.579707
O	4.066629	-6.266636	3.033056
C	5.265649	0.499684	3.931377
H	2.539430	0.502360	4.463022
C	5.922278	-2.958602	1.784086
H	3.418379	-4.226915	1.630587
O	-0.209067	-3.509295	7.720320
O	0.243644	-5.980996	6.261790
O	2.786765	-6.056079	7.161084
O	2.328928	-3.608189	8.632893
H	-0.856453	-3.873994	5.247886
C	-1.787023	-2.681019	2.975095
C	-0.975148	-2.522021	7.576926
C	1.652418	0.849211	6.976496
C	-0.247993	-6.504687	5.228561
C	3.071438	-6.760835	2.432910
H	1.206090	-2.977819	-0.862255
H	4.391998	-4.027115	7.123930
C	6.619383	-2.942714	5.993017
C	3.061251	-2.668426	9.038046
C	3.797766	-6.616367	6.663611
H	6.118746	1.177678	3.730464
H	6.865246	-2.885754	1.204738
H	-2.786307	-2.577892	2.503493
H	-1.759234	-2.381102	8.349236
H	1.456611	1.732440	7.619348
H	-0.836007	-7.434692	5.370170
H	3.223889	-7.750440	1.954709
H	7.693032	-2.912804	6.272685
H	3.178391	-2.563585	10.136330
H	4.112181	-7.577277	7.120696

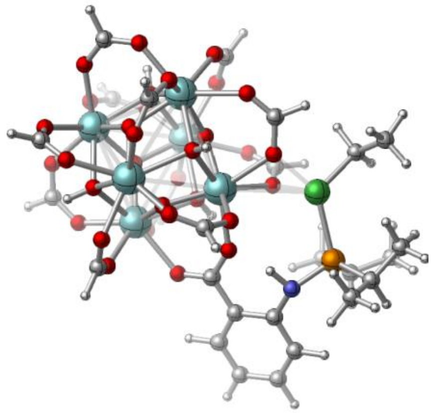

Zero-point correction= 0.721331 (Hartree/Particle)
Thermal correction to Energy= 0.797277
Thermal correction to Enthalpy= 0.798221
Thermal correction to Gibbs Free Energy= 0.618929
Sum of electronic and zero-point Energies= -5603.929372
Sum of electronic and thermal Energies= -5603.853427
Sum of electronic and thermal Enthalpies= -5603.852483
Sum of electronic and thermal Free Energies= -5604.031775

^{1}D-UiO66 (*i*-Pr)
$E(scf)_{gas}$ = -5683.19834524 a.u.
$E(scf)_{CPCM(heptane)}$ = -5683.24351308 a.u.
ν_{min} = 20.03 cm^{-1}

Ni	2.148375	1.520175	-1.404510	H	7.207472	0.718976	-1.018834
P	3.932316	2.364441	-0.082955	H	5.749920	1.016701	-1.989339
N	3.301874	2.531118	1.528862	H	6.748383	2.374972	-1.443361
H	2.642551	3.316893	1.551991	C	6.414708	2.120553	1.277619
C	2.691149	1.364000	2.085857	H	7.295872	1.489709	1.476419
C	1.355614	0.949359	1.808056	H	6.786098	3.112824	0.975044
C	3.459544	0.586129	2.964646	H	5.857640	2.249201	2.214522
C	0.834727	-0.186617	2.461906	C	4.058681	5.213405	0.364724
C	2.936327	-0.552881	3.580023	H	4.492650	6.159848	0.004682
H	4.478373	0.907678	3.181218	H	2.975350	5.372761	0.442688
C	1.612413	-0.935400	3.339725	H	4.454364	5.019952	1.372114
H	-0.200101	-0.463198	2.256795	H	5.517970	4.056700	-0.694364
H	1.191196	-1.813704	3.833327	H	1.310562	1.055554	-4.493265
C	5.559529	1.487801	0.171970	C	0.504816	0.702603	-3.830044
H	5.262839	0.469848	0.484481	C	0.542458	1.370755	-2.473819
C	4.419639	4.096481	-0.620158	H	-0.449880	0.927347	-4.340233
C	3.846639	4.344080	-2.024330	H	0.569839	-0.395302	-3.760267
H	4.167722	3.569437	-2.740953	C	2.200320	-0.588592	-1.250028
H	2.745299	4.373189	-2.003447	C	3.126647	-0.202117	-2.204149
H	4.192337	5.315658	-2.412410	H	4.183729	-0.129588	-1.946757
C	6.346657	1.393593	-1.145780	H	2.898842	-0.264469	-3.270907

H	2.502963	-0.769217	-0.212601	O	-1.382795	4.776749	-3.061765
H	1.220773	-0.984089	-1.530287	O	-3.366103	2.693560	-2.670302
H	0.494331	2.473049	-2.564609	O	-4.587918	8.283669	1.244431
H	-0.265515	1.007588	-1.816442	O	-2.953816	8.261359	-1.142666
H	3.561033	-1.131112	4.265186	O	-0.710755	8.252670	0.379240
C	0.459386	1.616815	0.822767	O	-2.346734	8.272169	2.765956
O	0.892631	2.649363	0.160255	C	-0.146170	4.859102	-2.886893
O	-0.688945	1.148938	0.641084	H	-1.073204	2.700161	-1.419783
Zr	-0.235009	4.705191	0.342512	C	0.446090	7.845187	0.113920
Zr	-2.711258	2.183509	0.829364	H	-0.355705	6.656540	2.391084
Zr	-2.218298	4.710560	3.307487	O	-6.211111	2.745536	1.421142
O	1.204885	4.525775	2.091905	O	-5.819420	4.797473	3.422530
Zr	-3.154298	4.688864	-1.666111	O	-6.165042	6.700695	1.538241
Zr	-2.673748	7.240761	0.818582	O	-6.549339	4.643874	-0.467913
O	-1.844756	5.737934	-0.398277	H	-4.263801	2.746086	3.126260
O	0.479144	4.764677	-1.789205	C	-1.987076	1.540372	3.929286
O	-1.546366	3.285257	-0.811973	C	-5.837709	1.553345	1.274980
O	-1.449766	3.643139	1.660305	C	-3.320776	1.503996	-2.263825
O	0.847883	6.644938	0.106633	C	-5.204274	4.858473	4.519766
O	-1.007616	6.100083	1.944586	C	-2.186100	7.873146	3.950312
Zr	-5.174052	4.721913	1.290461	H	1.943743	4.336374	3.962198
O	-3.813970	3.299482	2.473549	H	-4.972185	6.672603	-0.760950
O	-3.892160	3.664959	-0.003631	C	-3.123266	7.855438	-2.323419
O	-2.284315	1.127012	2.775552	C	-6.372419	4.562316	-1.711287
O	-4.654522	1.146072	1.105280	C	-5.753736	7.891124	1.521683
O	-3.047173	1.106929	-1.096766	H	0.478620	5.008071	-3.793611
O	-3.956870	4.788578	4.691870	H	1.204897	8.618283	-0.129513
O	-0.049658	4.629088	3.964324	H	-1.735717	0.764656	4.683190
O	-1.951327	2.735556	4.322210	H	-6.628072	0.774980	1.309960
O	-2.054807	6.679707	4.334861	H	-3.530241	0.714278	-3.015872
O	-3.491286	5.741005	2.021114	H	-5.824986	4.973516	5.432493
C	1.030246	4.496109	3.350433	H	-2.142385	8.655378	4.736365
O	-4.322219	6.115273	-0.311824	H	-3.162812	8.633537	-3.114057
O	-3.265396	6.661371	-2.701856	H	-7.281477	4.451555	-2.338098
O	-5.269570	4.606272	-2.323509	H	-6.497291	8.678028	1.765086

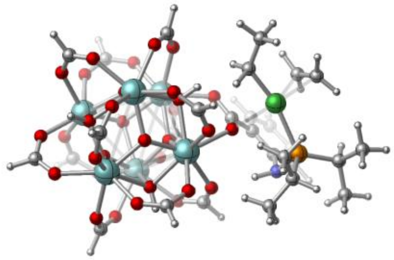

Zero-point correction= 0.777807 (Hartree/Particle)

Thermal correction to Energy= 0.856160
Thermal correction to Enthalpy= 0.857104
Thermal correction to Gibbs Free Energy= 0.674804
Sum of electronic and zero-point Energies= -5682.420538
Sum of electronic and thermal Energies= -5682.342185
Sum of electronic and thermal Enthalpies= -5682.341241
Sum of electronic and thermal Free Energies= -5682.523542

^{3}D-UiO66 (*i*-Pr)
$E(scf)_{gas}$ = -5683.20766521 a.u.
$E(scf)_{CPCM(heptane)}$ = -5683.25359036 a.u.
ν_{min} = 16.40 cm^{-1}

Ni	2.295904	3.754368	-1.290677
P	3.541418	2.132959	-0.055677
N	2.291096	1.324364	0.801757
H	1.415875	1.684516	0.432558
C	2.136412	-0.015676	1.182877
C	0.902856	-0.686643	0.933336
C	3.150640	-0.709538	1.862106
C	0.716038	-1.994075	1.418352
C	2.959076	-2.022490	2.289697
H	4.088742	-0.200334	2.077549
C	1.736235	-2.671301	2.079092
H	-0.249104	-2.472384	1.242791
H	1.582384	-3.692901	2.431260
C	4.494064	0.821686	-0.974137
H	4.950725	0.179482	-0.203803
C	4.730776	2.877765	1.188396
C	3.939071	3.526664	2.331707
H	3.202441	4.253552	1.964205
H	3.403987	2.767023	2.921666
H	4.624655	4.065369	3.003749
C	3.552573	-0.054336	-1.816085
H	4.146614	-0.719230	-2.462898
H	2.916549	-0.688837	-1.184291
H	2.884599	0.535368	-2.460572
C	5.625675	1.462684	-1.794315
H	6.214273	0.677528	-2.294343
H	5.243945	2.134650	-2.577415
H	6.320616	2.046430	-1.172152
C	5.835021	1.969320	1.744673
H	6.526278	2.573711	2.353316
H	5.425446	1.193324	2.408602
H	6.434235	1.476689	0.966690
H	5.203814	3.679550	0.592143
H	1.604545	6.697559	-2.113503
C	1.709017	6.588561	-1.022228
C	2.797783	5.596872	-0.638538
H	1.908250	7.600961	-0.624010
H	0.719471	6.291813	-0.631885
C	3.167268	4.330522	-3.396073
C	2.747315	3.055978	-3.559856
H	1.713530	2.809102	-3.816057
H	3.455661	2.229245	-3.512850
H	2.498547	5.178901	-3.557532
H	4.219365	4.569192	-3.216143
H	3.776857	5.885875	-1.055767
H	2.909567	5.539222	0.456337
H	3.768724	-2.537081	2.813008
C	-0.208793	-0.062359	0.161347
O	0.079936	0.888801	-0.651532
O	-1.380477	-0.498916	0.326519
Zr	-1.074252	2.508413	-1.629568
Zr	-3.398522	0.458708	0.090763
Zr	-3.064434	3.928737	0.940608
O	0.502550	3.524416	-0.173047
Zr	-3.949841	1.350232	-3.333163
Zr	-3.612257	4.825084	-2.471643
O	-2.699232	2.957603	-2.785004
O	-0.299524	1.539466	-3.495733
O	-2.289212	0.636053	-1.913414
O	-2.239875	2.223266	0.046097
O	0.000437	4.161008	-2.786443
O	-1.904344	4.494995	-0.952320
Zr	-5.991138	2.773277	-0.757408
O	-4.574092	2.219173	0.963918
O	-4.642490	1.284680	-1.364079
O	-2.910072	0.587153	2.278379
O	-5.291495	-0.360409	0.906779
O	-3.746825	-1.430966	-1.041466

O	-4.811651	4.623937	2.115043	O	-7.347563	1.770361	-2.212917
O	-0.827785	4.280230	1.485136	H	-5.003652	2.050851	1.813641
O	-2.678070	2.771599	2.793211	C	-2.643217	1.537404	3.058577
O	-2.967710	6.152483	0.834276	C	-6.495119	0.015846	0.857018
O	-4.369433	4.112540	-0.665344	C	-4.027622	-1.686922	-2.243297
C	0.278301	4.069099	0.975635	C	-6.060824	4.533104	1.956204
O	-5.190378	3.218563	-2.860069	C	-3.155553	6.981065	-0.098738
O	-4.119835	2.532768	-5.216606	H	1.180792	4.376844	1.536218
O	-6.059198	0.878549	-3.832817	H	-5.862092	3.447002	-3.517001
O	-2.139170	0.795256	-4.572371	C	-4.033410	3.759384	-5.490209
O	-4.090525	-0.865702	-3.197445	C	-7.161652	1.093772	-3.258467
O	-5.566920	5.858773	-2.595806	C	-6.717222	5.607393	-2.140281
O	-3.900896	4.711495	-4.673558	H	-0.270541	0.646998	-5.308456
O	-1.663197	5.562150	-3.392416	H	0.236254	5.747525	-4.019943
O	-3.332200	6.719100	-1.318266	H	-2.354162	1.264869	4.095120
C	-0.907231	0.993137	-4.466726	H	-7.246699	-0.669274	1.300878
H	-1.794746	-0.158091	-2.156862	H	-4.228837	-2.749999	-2.490531
C	-0.481016	5.161126	-3.407135	H	-6.693456	5.065515	2.696228
H	-1.290239	5.236603	-0.877442	H	-3.149729	8.054387	0.183531
O	-6.929025	1.092028	0.369129	H	-4.090675	4.033602	-6.564180
O	-6.660944	3.891888	1.055144	H	-8.061804	0.649821	-3.731558
O	-7.073388	4.569625	-1.524171	H	-7.496407	6.378385	-2.312483

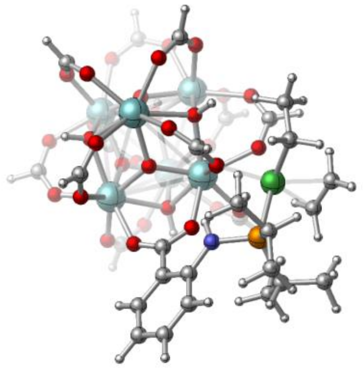

Zero-point correction= 0.775550 (Hartree/Particle)
Thermal correction to Energy= 0.855086
Thermal correction to Enthalpy= 0.856030
Thermal correction to Gibbs Free Energy= 0.669995
Sum of electronic and zero-point Energies= -5682.432115
Sum of electronic and thermal Energies= -5682.352579
Sum of electronic and thermal Enthalpies= -5682.351635
Sum of electronic and thermal Free Energies= -5682.537670

^{1}TS-D-E-UiO66 (*i*-Pr)
$E(scf)_{gas}$ = -5683.18915309 a.u.
$E(scf)_{CPCM(heptane)}$ = -5683.23628926 a.u.
ν_{min} = -278.50 cm^{-1}

Ni	2.342980	2.799021	-1.935348
P	3.681077	1.944912	-0.343887
N	2.760474	1.212964	0.917702
H	2.393032	1.932777	1.546859
C	1.825080	0.188204	0.795964
C	0.636507	0.188300	1.594399
C	2.029454	-0.898855	-0.078633
C	-0.230000	-0.921970	1.552401
C	1.134762	-1.966628	-0.126467
H	2.916581	-0.926088	-0.709468
C	0.006769	-1.996870	0.703004
H	-1.119205	-0.889825	2.183469
H	-0.679857	-2.844943	0.676736
C	5.037498	0.722366	-0.759995
H	4.556195	0.001709	-1.442613
C	4.594476	3.331985	0.551729
C	4.486103	4.621897	-0.275936
H	4.809907	4.470689	-1.317950
H	3.451884	5.001381	-0.281382
H	5.126553	5.403905	0.162042
C	6.187293	1.393524	-1.529953
H	6.842313	0.617130	-1.954395
H	5.843993	2.027512	-2.359012
H	6.811705	2.012617	-0.868236
C	5.544906	-0.024607	0.478767
H	6.322637	-0.746074	0.181420
H	5.999357	0.667284	1.206207
H	4.744602	-0.574011	0.993019
C	4.191058	3.550734	2.014166
H	4.840812	4.330244	2.442976
H	3.158264	3.910783	2.121683
H	4.317305	2.642212	2.620317
H	5.643920	3.001254	0.544536
H	3.183848	5.359576	-3.382568
C	2.151973	5.276013	-3.758937
C	1.370773	4.228244	-2.994728
H	1.677001	6.263234	-3.617908
H	2.194284	5.092226	-4.843221
C	2.088795	2.391288	-3.931038
C	3.411046	2.171181	-3.441809
H	3.750820	1.145096	-3.271909
H	4.200280	2.846189	-3.795337
H	1.358253	1.572231	-3.896249
H	1.933818	3.039026	-4.793123

H	1.286722	4.538401	-1.939227
H	0.362253	4.045191	-3.391954
H	1.331672	-2.794283	-0.812596
C	0.172699	1.381858	2.343999
O	-0.864732	1.318263	3.055026
O	0.808538	2.491669	2.155629
Zr	-2.755068	2.581353	2.879855
Zr	-0.107406	4.270799	1.192579
Zr	-3.038820	3.278941	-0.609489
O	-3.174218	0.630864	1.845924
Zr	-2.100687	6.039308	3.532223
Zr	-5.035573	5.094769	1.724051
O	-3.450327	4.545005	2.982086
O	-2.491131	2.783708	5.068834
O	-1.023434	4.035854	3.239303
O	-1.781575	3.032302	1.057137
O	-4.750695	2.113817	3.744484
O	-4.308379	2.975187	1.246079
Zr	-2.352319	6.728337	0.002423
O	-1.285264	4.771073	-0.663331
O	-1.259451	5.908683	1.593441
O	0.650082	2.794577	-0.609234
O	1.157358	5.678611	-0.019689
O	1.306494	5.243532	2.632199
O	-3.049094	4.298550	-2.596448
O	-3.362524	1.076130	-0.356443
O	-1.290769	2.189365	-1.590347
O	-5.141488	3.129418	-1.305949
O	-3.662580	5.114393	0.119296
C	-3.327702	0.304883	0.638426
O	-3.561600	6.826573	1.947395
O	-3.836244	6.886337	4.618796
O	-1.669990	8.213224	3.222901
O	-2.130012	4.969851	5.491059
O	0.032429	6.331801	4.138680
O	-5.891984	6.853661	0.641866
O	-5.689785	6.296566	3.481572
O	-6.188622	3.686646	3.009641
O	-6.409117	4.248680	0.182728
C	-2.311960	3.772467	5.833422
H	-0.389878	3.715386	3.896513
C	-5.877179	2.678850	3.697914
H	-5.005564	2.309852	1.168847
O	-0.271284	7.270007	-0.738199

O	-2.650922	6.484508	-2.203050	C	-1.581776	8.954013	2.209152
O	-4.197546	7.866333	-0.446419	C	-5.412443	7.718418	-0.135884
O	-1.821874	8.635272	1.011730	H	-2.304603	3.558992	6.922314
H	-0.805158	4.810678	-1.499840	H	-6.681320	2.233248	4.319472
C	-0.108020	1.995445	-1.274430	H	0.359157	1.041956	-1.602387
C	0.868429	6.781935	-0.577577	H	1.725795	7.359680	-0.984415
C	1.131122	5.979403	3.644331	H	2.047517	6.344806	4.153690
C	-2.947747	5.508460	-2.940811	H	-3.124171	5.733027	-4.013805
C	-6.233679	3.594417	-0.877772	H	-7.133425	3.395263	-1.496060
H	-3.453185	-0.778110	0.429215	H	-5.713697	7.306521	5.228666
H	-3.961103	7.684789	2.144161	H	-1.269709	10.004402	2.386488
C	-5.084890	6.828493	4.449504	H	-6.128562	8.434698	-0.589260

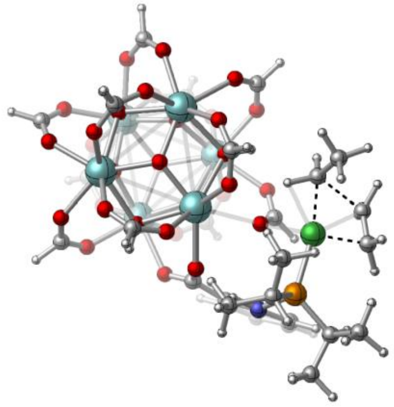

Zero-point correction= 0.777697 (Hartree/Particle)
Thermal correction to Energy= 0.855135
Thermal correction to Enthalpy= 0.856079
Thermal correction to Gibbs Free Energy= 0.676580
Sum of electronic and zero-point Energies= -5682.411457
Sum of electronic and thermal Energies= -5682.334018
Sum of electronic and thermal Enthalpies= -5682.333074
Sum of electronic and thermal Free Energies= -5682.512573

[3]TS-D-E-UiO66 (*i*-Pr)
$E(scf)_{gas}$ = -5683.15681861 a.u.
$E(scf)_{CPCM(heptane)}$ = -5683.20547441 a.u.
ν_{min} = -421.02 cm^{-1}

Ni	4.625884	0.657022	-1.231547	C	3.166243	-1.855611	0.470853
P	3.756092	2.495014	-0.155952	H	4.185051	-0.047253	1.045557
N	2.318781	1.765750	0.474923	C	1.941150	-2.443384	0.164242
H	1.460825	2.303709	0.340546	H	-0.163541	-2.056072	-0.192736
C	2.157080	0.383929	0.383429	H	1.847930	-3.527357	0.078928
C	0.907883	-0.227165	0.086773	C	4.661337	3.105423	1.346612
C	3.278318	-0.460973	0.593996	H	4.663802	2.195025	1.973077
C	0.818432	-1.622941	0.010636	C	3.081046	3.927725	-1.122784

C	4.164965	4.938223	-1.516816
H	4.606780	5.451384	-0.651640
H	4.979577	4.464148	-2.090040
H	3.717638	5.709477	-2.163215
C	6.120540	3.466386	1.037845
H	6.666267	3.643863	1.977712
H	6.631445	2.651722	0.500403
H	6.205003	4.380957	0.433059
C	3.896125	4.198442	2.099881
H	4.391332	4.410508	3.060566
H	3.861412	5.140551	1.531238
H	2.863081	3.887006	2.317513
C	2.332532	3.390173	-2.350205
H	1.741247	4.199037	-2.804237
H	3.035378	3.008674	-3.108930
H	1.624821	2.589311	-2.099689
H	2.349281	4.410172	-0.451837
H	4.804093	1.546521	-3.715635
C	4.626052	0.519882	-4.086069
C	4.222605	-0.450543	-2.991541
H	3.831268	0.601904	-4.847426
H	5.545360	0.194807	-4.596218
C	5.887759	-0.978217	-1.876943
C	6.478980	-0.115312	-0.883739
H	6.533038	-0.455853	0.155682
H	7.284052	0.563157	-1.187718
H	5.502993	-1.942344	-1.529775
H	6.409917	-1.047177	-2.835929
H	3.239309	-0.208829	-2.526075
H	4.146261	-1.487033	-3.332017
H	4.046207	-2.477289	0.654860
C	-0.326888	0.587940	-0.151743
O	-1.439946	0.106429	0.168833
O	-0.139472	1.743190	-0.655109
Zr	-3.542053	1.017011	0.341498
Zr	-1.428030	3.518112	-1.052073
Zr	-4.367121	2.524241	-2.797865
O	-3.697301	-0.636284	-1.145674
Zr	-3.358738	4.251778	1.848717
Zr	-6.315718	3.262540	0.104297
O	-4.522377	2.716584	1.048303
O	-3.056557	0.704895	2.506005
O	-2.021544	2.616161	0.959789
O	-2.922198	2.110458	-1.329525
O	-5.306007	-0.038566	1.192517
O	-5.329381	1.501715	-0.992833
Zr	-4.168433	5.763323	-1.293960
O	-2.927176	4.301048	-2.558109
O	-2.755069	4.774651	-0.091139
O	-0.757755	2.793552	-3.080569
O	-0.573246	5.426536	-1.859274
O	0.080976	4.245600	0.524010
O	-4.871563	4.021549	-4.376359
O	-4.241377	0.306592	-3.123037
O	-2.632395	2.197741	-4.188941
O	-6.492216	2.125092	-3.322721
O	-5.187412	3.954172	-1.521492
C	-3.940969	-0.665318	-2.383635
O	-5.115739	5.128091	0.690999
O	-5.051496	4.442531	3.279170
O	-3.305007	6.460966	2.148129
O	-2.943542	2.747834	3.458141
O	-1.193592	4.767288	2.316748
O	-7.577546	5.044183	-0.358977
O	-6.920448	3.824223	2.179989
O	-7.058353	1.373636	1.045827
O	-7.723841	2.593441	-1.493396
C	-2.944089	1.488240	3.487455
H	-1.320184	2.329758	1.557436
C	-6.505105	0.298953	1.395156
H	-5.917564	0.762139	-1.196403
O	-2.322922	6.846110	-2.018789
O	-4.737815	6.064162	-3.429365
O	-6.222720	6.618242	-1.237075
O	-3.820299	7.414811	0.169538
H	-2.573699	4.663610	-3.381002
C	-1.388843	2.319512	-4.065981
C	-1.106847	6.549124	-2.099090
C	-0.117225	4.729197	1.679342
C	-4.995367	5.275454	-4.378181
C	-7.580052	2.261818	-2.699515
H	-3.894540	-1.662600	-2.870648
H	-5.617259	5.815024	1.150500
C	-6.285331	4.191356	3.202888
C	-3.472757	7.446312	1.379680
C	-7.346202	6.161575	-0.891760
H	-2.815502	1.016319	4.484515
H	-7.141620	-0.437802	1.928510
H	-0.772770	1.981930	-4.927566
H	-0.419012	7.357287	-2.428355

H	0.775735	5.151570	2.190346	H	-6.874544	4.319345	4.134886
H	-5.357493	5.736581	-5.320891	H	-3.306528	8.451365	1.821343
H	-8.508092	2.055891	-3.272594	H	-8.222656	6.820256	-1.065207

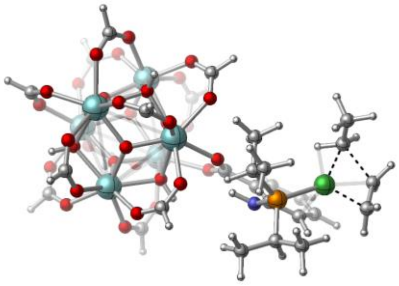

Zero-point correction= 0.773413 (Hartree/Particle)
Thermal correction to Energy= 0.852481
Thermal correction to Enthalpy= 0.853425
Thermal correction to Gibbs Free Energy= 0.665643
Sum of electronic and zero-point Energies= -5682.383406
Sum of electronic and thermal Energies= -5682.304338
Sum of electronic and thermal Enthalpies= -5682.303393
Sum of electronic and thermal Free Energies= -5682.491175

^{1}E-UiO66 (*i*-Pr)
$E(scf)_{gas}$ = -5683.24404970 a.u.
$E(scf)_{CPCM(heptane)}$ = -5683.28947208 a.u.
ν_{min} = 28.30 cm^{-1}

Ni	1.607787	2.632869	-1.542860	C	4.943542	0.611911	-2.711249
P	3.239619	1.543906	-0.675513	H	5.224451	-0.233694	-3.358010
N	2.743433	0.954409	0.871063	H	4.492921	1.382089	-3.351278
H	2.727232	1.743049	1.523460	H	5.873558	1.019092	-2.286884
C	1.680551	0.093105	1.158340	C	4.674911	-0.952224	-0.731658
C	0.881534	0.296004	2.327002	H	4.891401	-1.839089	-1.348299
C	1.389709	-1.026546	0.353109	H	5.630672	-0.591690	-0.329413
C	-0.040833	-0.693759	2.718909	H	4.058844	-1.271707	0.118404
C	0.429283	-1.962291	0.735573	C	5.753881	2.080789	0.597785
H	1.937221	-1.190450	-0.572361	H	6.472294	2.866085	0.881512
C	-0.268890	-1.823094	1.940535	H	5.381249	1.616931	1.523759
H	-0.609003	-0.517653	3.632579	H	6.307572	1.319371	0.033596
H	-0.995257	-2.575562	2.253011	H	4.991454	3.017393	-1.204484
C	3.994616	0.109624	-1.609850	H	-1.084790	5.236348	-4.493842
H	3.118538	-0.341770	-2.106762	C	-0.420169	5.724708	-3.763030
C	4.614372	2.708967	-0.213964	C	0.976632	5.106736	-3.788028
C	4.037790	3.946234	0.484411	H	-0.392115	6.798520	-4.000852
H	3.220386	4.413644	-0.083978	H	-0.893622	5.630264	-2.770671
H	3.649900	3.715663	1.486840	C	0.986061	3.612551	-3.451521
H	4.826408	4.703188	0.616916	C	2.330293	2.959143	-3.247521

H	2.513531	2.077110	-3.877224
H	3.190211	3.647110	-3.286616
H	0.361962	3.537535	-2.481796
H	0.354159	3.039687	-4.149504
H	1.429245	5.231905	-4.785468
H	1.645892	5.625393	-3.082339
H	0.239379	-2.821983	0.088080
C	0.809845	1.599265	3.035099
O	0.118042	1.709958	4.081176
O	1.376463	2.616180	2.470900
Zr	-1.670328	3.086987	4.432222
Zr	0.231894	4.357080	1.698391
Zr	-3.189362	3.410708	1.217875
O	-2.512831	1.077541	3.887468
Zr	-0.708338	6.531679	4.343455
Zr	-4.130155	5.629410	3.847920
O	-2.213356	5.096957	4.516173
O	-0.617434	3.506381	6.332947
O	0.124044	4.425859	3.949870
O	-1.416866	3.253581	2.339252
O	-3.225974	2.879765	6.009334
O	-3.700306	3.419355	3.427431
Zr	-2.205385	6.841460	1.079774
O	-1.526096	4.735823	0.339246
O	-0.635370	6.116941	2.266154
O	0.203380	2.644155	-0.088489
O	0.996459	5.470310	-0.102531
O	2.093040	5.395243	2.391977
O	-3.899233	4.209240	-0.743381
O	-3.470325	1.284663	1.856590
O	-1.979183	2.084262	-0.189364
O	-5.404440	3.351533	1.350876
O	-3.436263	5.360309	1.870953
C	-3.108598	0.631469	2.870251
O	-2.618731	7.250514	3.297116
O	-1.896818	7.630241	5.856753
O	-0.348605	8.617621	3.614871
O	-0.057964	5.691140	6.306610
O	1.501604	6.725424	4.111803
O	-5.261667	7.319067	2.920130
O	-4.056239	7.067153	5.547030
O	-4.779503	4.469920	5.636434
O	-6.000075	4.730235	3.029895
C	-0.140823	4.557248	6.845981
H	0.940713	4.130969	4.376492
C	-4.271774	3.523944	6.295053
H	-4.399697	2.808445	3.696375
O	-0.507814	7.127077	-0.397331
O	-3.280150	6.377369	-0.827805
O	-4.050846	8.061113	1.169685
O	-1.289311	8.804380	1.572939
H	-1.370923	4.666797	-0.610973
C	-0.781426	1.877976	-0.415073
C	0.586390	6.559619	-0.609831
C	2.326379	6.245173	3.295844
C	-3.872250	5.356017	-1.267964
C	-6.247678	3.948282	2.075382
H	-3.347115	-0.452478	2.870673
H	-2.889518	8.154458	3.507886
C	-3.122259	7.653662	6.154836
C	-0.616177	9.233695	2.550426
C	-5.071840	8.047895	1.912390
H	0.255503	4.466092	7.878405
H	-4.807220	3.216259	7.217091
H	-0.513480	0.952017	-0.966262
H	1.270072	7.048028	-1.334694
H	3.375475	6.598660	3.382985
H	-4.415246	5.473388	-2.229082
H	-7.317496	3.753609	1.854076
H	-3.406732	8.263977	7.036621
H	-0.228526	10.270280	2.464733
H	-5.880152	8.762684	1.653354

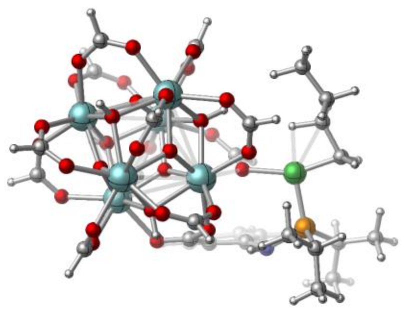

Zero-point correction= 0.782260 (Hartree/Particle)
Thermal correction to Energy= 0.859520
Thermal correction to Enthalpy= 0.860464
Thermal correction to Gibbs Free Energy= 0.681230
Sum of electronic and zero-point Energies= -5682.461790
Sum of electronic and thermal Energies= -5682.384530
Sum of electronic and thermal Enthalpies= -5682.383586
Sum of electronic and thermal Free Energies= -5682.562820

[3]E-UiO66 (*i*-Pr)
$E(scf)_{gas}$ = -5683.22158944 a.u.
$E(scf)_{CPCM(heptane)}$ = -5683.27163009 a.u.
ν_{min} = 15.11 cm^{-1}

Ni	2.402753	4.231822	-1.304727
P	3.512684	2.269606	-0.768940
N	3.161084	2.254971	0.905118
H	2.446102	2.960266	1.087971
C	2.942452	1.133243	1.733503
C	1.640205	0.839254	2.219674
C	4.012819	0.300623	2.091798
C	1.459874	-0.274630	3.061264
C	3.809725	-0.803383	2.919000
H	5.012729	0.544247	1.727661
C	2.531445	-1.089602	3.415116
H	0.452404	-0.491440	3.419901
H	2.374214	-1.946484	4.073091
C	2.917491	0.698131	-1.597799
H	2.989367	0.979458	-2.664636
C	5.358025	2.279703	-0.957750
C	5.723539	2.362507	-2.446849
H	5.362532	1.500394	-3.028048
H	5.314328	3.280600	-2.902997
H	6.817814	2.401318	-2.563013
C	3.765258	-0.561728	-1.374753
H	3.347027	-1.389241	-1.970459
H	4.811004	-0.432450	-1.688492
H	3.754431	-0.872197	-0.320345
C	1.436835	0.443657	-1.273410
H	0.988011	-0.220614	-2.027524
H	1.321481	-0.037738	-0.292401
H	0.869202	1.382404	-1.259002
C	5.975496	3.428579	-0.152860
H	7.073957	3.365677	-0.197639
H	5.679381	4.404745	-0.567392
H	5.669862	3.399371	0.903021
H	5.714851	1.320771	-0.544551
H	3.217689	8.509871	-3.017070
C	4.178281	8.202524	-2.571214
C	4.040177	6.877076	-1.824902
H	4.921148	8.140223	-3.381327
H	4.496218	9.005296	-1.886147
C	3.001789	6.909508	-0.695008
C	2.865770	5.603227	0.075082
H	3.807084	5.300248	0.562678
H	2.068898	5.639548	0.834056
H	3.273727	7.725492	0.005571
H	2.019919	7.195765	-1.108442
H	3.767338	6.078905	-2.552177
H	5.018659	6.572387	-1.412283
H	4.658085	-1.435455	3.192469
C	0.427261	1.599012	1.787374

O	0.563080	2.760370	1.265734
O	-0.686898	1.022201	1.923496
Zr	-0.900479	4.289910	0.479008
Zr	-2.816883	1.492382	1.495142
Zr	-3.517689	4.749919	2.834637
O	0.047527	5.223546	2.268002
Zr	-3.173802	2.776130	-1.815733
Zr	-3.856474	6.024232	-0.493285
O	-2.443482	4.505625	-0.890343
O	0.438688	3.788448	-1.524340
O	-1.532172	2.269374	-0.294969
O	-2.177860	3.459001	1.845809
O	-0.236089	6.208914	-0.464975
O	-2.326297	5.878825	1.214252
Zr	-5.807767	3.214156	0.552835
O	-4.515750	2.735598	2.382028
O	-4.049204	2.205398	-0.028288
O	-2.657097	1.343494	3.718490
O	-4.503276	0.101855	1.866846
O	-2.490418	-0.210202	0.064613
O	-5.521321	4.783877	3.790515
O	-1.613997	5.509899	3.766001
O	-3.099703	3.391806	4.553775
O	-4.036733	6.907173	3.000337
O	-4.616045	4.862663	1.061804
C	-0.414097	5.550117	3.400720
O	-4.901864	4.169220	-1.329786
O	-3.404679	4.144737	-3.554741
O	-4.996008	1.872142	-2.703555
O	-1.116477	2.894766	-2.894038
O	-2.684164	0.609873	-2.028007
O	-5.990782	6.556133	-0.809292
O	-3.849790	6.194752	-2.726547
O	-2.107849	7.347112	-1.007793
O	-4.247493	7.712584	0.905385
C	0.013353	3.319222	-2.649763
H	-0.855926	1.636902	-0.567962
C	-0.865943	7.188123	-0.962563
H	-1.935956	6.717344	1.495124
O	-6.392842	1.183658	1.281999
O	-6.965236	3.812929	2.358274
O	-7.221785	4.787970	-0.147039
O	-6.666395	2.159471	-1.218342
H	-4.985781	2.330039	3.123204
C	-2.765028	2.181935	4.654363
C	-5.749642	0.178230	1.679736
C	-2.541112	-0.321225	-1.187914
C	-6.671169	4.419955	3.422019
C	-4.310515	7.806559	2.160683
H	0.323504	5.922000	4.142250
H	-5.526199	4.332831	-2.049697
C	-3.627562	5.382927	-3.661335
C	-6.179873	1.720409	-2.292358
C	-7.079335	5.965742	-0.570040
H	0.766934	3.282609	-3.467826
H	-0.243145	7.998373	-1.398825
H	-2.552553	1.810471	5.678698
H	-6.339098	-0.735656	1.900948
H	-2.431648	-1.345963	-1.601329
H	-7.508863	4.648163	4.112984
H	-4.627801	8.787987	2.570063
H	-3.637145	5.795379	-4.692033
H	-6.864092	1.149652	-2.953873
H	-8.008354	6.540609	-0.764527

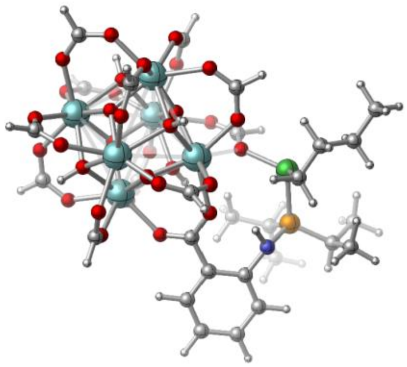

Zero-point correction= 0.778059 (Hartree/Particle)
Thermal correction to Energy= 0.856775

Thermal correction to Enthalpy= 0.857719
Thermal correction to Gibbs Free Energy= 0.671674
Sum of electronic and zero-point Energies= -5682.443530
Sum of electronic and thermal Energies= -5682.364815
Sum of electronic and thermal Enthalpies= -5682.363871
Sum of electronic and thermal Free Energies= -5682.549916

[1]TS-E-F-UiO66 (*i*-Pr)
$E(scf)_{gas}$ = -5683.22479114 a.u.
$E(scf)_{CPCM(heptane)}$ = -5683.270952 a.u.
ν_{min} = -9.25 cm^{-1}

Ni	3.078036	3.501415	-1.889796
P	4.103983	1.920429	-0.736805
N	3.499303	1.898439	0.865625
H	2.640586	2.448977	0.876324
C	3.332037	0.768240	1.695626
C	2.031405	0.271849	1.988503
C	4.447608	0.121951	2.248640
C	1.894450	-0.846842	2.830894
C	4.289768	-0.991285	3.073747
H	5.442688	0.518354	2.039884
C	3.010394	-1.476165	3.374833
H	0.888406	-1.216475	3.036252
H	2.888024	-2.340759	4.030078
C	3.871123	0.249220	-1.569951
H	4.020818	0.523059	-2.630714
C	5.945204	2.120835	-0.578953
C	6.592233	2.223938	-1.966745
H	6.420096	1.332496	-2.588641
H	6.204145	3.097188	-2.516789
H	7.681266	2.351982	-1.866849
C	4.879087	-0.850983	-1.207908
H	4.651728	-1.754418	-1.796869
H	5.918691	-0.573598	-1.430056
H	4.810889	-1.123411	-0.144791
C	2.428837	-0.258683	-1.407513
H	2.178781	-0.965515	-2.213565
H	2.300749	-0.783630	-0.451068
H	1.712702	0.569641	-1.436097
C	6.286673	3.324444	0.305702
H	7.377906	3.392869	0.438167
H	5.950103	4.268779	-0.154334
H	5.819747	3.248299	1.298191
H	6.303284	1.205905	-0.078858
H	4.323979	8.176800	-2.216663
C	3.352863	7.893703	-1.779639
C	3.342517	6.425678	-1.330329
H	3.162158	8.560975	-0.925234
H	2.578178	8.081750	-2.540368
C	3.637350	5.498755	-2.488192
C	2.655559	4.951429	-3.297192
H	1.603929	5.176161	-3.120848
H	2.919615	4.551352	-4.283100
H	4.657511	5.555954	-2.886352
H	4.365497	4.046314	-1.614805
H	4.096890	6.274239	-0.541154
H	2.365235	6.166375	-0.895547
H	5.172454	-1.475006	3.499181
C	0.806774	0.831764	1.348797
O	0.885424	1.976393	0.775493
O	-0.243335	0.135617	1.360511
Zr	-0.513528	3.299563	-0.312361
Zr	-2.328310	0.395601	0.586292
Zr	-3.483051	3.637102	1.610906
O	0.079936	4.410583	1.536470
Zr	-2.281292	1.482797	-2.815553
Zr	-3.415988	4.719983	-1.798103
O	-1.836515	3.319686	-1.901955
O	1.202259	2.773275	-2.105725
O	-0.861629	1.193461	-1.038822
O	-1.912343	2.427393	0.904026
O	0.139252	5.236083	-1.221176
O	-2.159108	4.788983	0.117638
Zr	-5.270488	1.800630	-0.889238
O	-4.236644	1.525543	1.137454
O	-3.370547	0.926795	-1.141534
O	-2.493843	0.368276	2.812038
O	-3.938989	-1.116164	0.789366
O	-1.657079	-1.338386	-0.663598
O	-5.605512	3.540409	2.257100
O	-1.806270	4.604728	2.759129

O	-3.222045	2.405268	3.451127	H	-4.780085	1.115746	1.824123
O	-4.193235	5.747332	1.569716	C	-2.810107	1.235658	3.671091
O	-4.308091	3.571323	-0.308853	C	-5.143936	-1.159194	0.415436
C	-0.571867	4.737600	2.570448	C	-1.521158	-1.519607	-1.902488
O	-4.167266	2.745813	-2.670765	C	-6.653128	3.060199	1.744810
O	-2.357113	2.759073	-4.646221	C	-4.410848	6.578008	0.647212
O	-3.872299	0.392031	-3.911749	H	0.011398	5.203360	3.392401
O	-0.129519	1.691908	-3.574281	H	-4.684810	2.820107	-3.484072
O	-1.619315	-0.651007	-2.811491	C	-2.648909	3.968961	-4.849797
O	-5.510899	5.047123	-2.459659	C	-5.088184	0.154805	-3.669061
O	-3.069497	4.800651	-4.002791	C	-6.572798	4.374927	-2.349612
O	-1.710202	6.187338	-2.101086	H	1.769141	2.194371	-3.970729
O	-4.150178	6.432709	-0.577551	H	0.122476	7.015725	-2.175275
C	0.937664	2.217066	-3.235321	H	-2.726584	0.931762	4.735481
H	-0.103310	0.613011	-1.181985	H	-5.685613	-2.109573	0.602363
C	-0.489007	6.151032	-1.837916	H	-1.272325	-2.551241	-2.228944
H	-1.888611	5.668591	0.412671	H	-7.601805	3.246274	2.289500
O	-5.797150	-0.234738	-0.134147	H	-4.867596	7.544151	0.946421
O	-6.732584	2.380664	0.687326	H	-2.530511	4.337881	-5.890306
O	-6.683642	3.210628	-1.883102	H	-5.617843	-0.501467	-4.390333
O	-5.763339	0.595355	-2.703036	H	-7.503881	4.856481	-2.713747

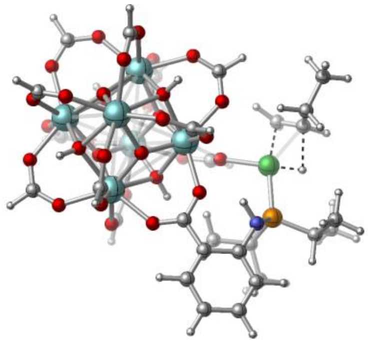

Zero-point correction= 0.776659 (Hartree/Particle)
Thermal correction to Energy= 0.854027
Thermal correction to Enthalpy= 0.854972
Thermal correction to Gibbs Free Energy= 0.675129
Sum of electronic and zero-point Energies= -5682.448132
Sum of electronic and thermal Energies= -5682.370764
Sum of electronic and thermal Enthalpies= -5682.369819
Sum of electronic and thermal Free Energies= -5682.549662

^{3}TS-E-F-UiO66 (*i*-Pr)
$E(scf)_{gas}$ = -5683.19475450 a.u.
$E(scf)_{CPCM(heptane)}$ = -5683.24207999 a.u.
ν_{min} = -688.61 cm^{-1}

Ni	2.586904	4.270274	-1.494504	P	3.538201	2.286679	-0.711616

N	3.163783	2.304011	0.957332	C	0.430002	1.592665	1.752883
H	2.411205	2.979350	1.097539	O	0.565775	2.768666	1.267370
C	2.949980	1.183992	1.792065	O	-0.675039	0.988744	1.824229
C	1.639256	0.853295	2.225881	Zr	-0.881115	4.327953	0.517013
C	4.029484	0.389760	2.204952	Zr	-2.797128	1.480157	1.396880
C	1.453822	-0.262584	3.062805	Zr	-3.496451	4.665743	2.895326
C	3.823820	-0.715857	3.029687	O	0.079308	5.192071	2.338145
H	5.036384	0.661010	1.882574	Zr	-3.142052	2.930006	-1.847870
C	2.533939	-1.042119	3.467809	Zr	-3.844252	6.104772	-0.361566
H	0.438241	-0.507041	3.378656	O	-2.422876	4.615726	-0.834116
H	2.374690	-1.901573	4.121973	O	0.480092	3.924637	-1.456455
C	2.940823	0.715503	-1.536698	O	-1.509565	2.346145	-0.341138
H	3.118182	0.958582	-2.600466	O	-2.156937	3.428098	1.846992
C	5.390852	2.247598	-0.849089	O	-0.223323	6.315470	-0.310294
C	5.813355	2.228726	-2.324854	O	-2.312023	5.876715	1.335510
H	5.476765	1.326221	-2.856425	Zr	-5.785193	3.241094	0.537251
H	5.419066	3.108944	-2.862356	O	-4.495417	2.675180	2.343098
H	6.911304	2.264245	-2.401200	O	-4.023457	2.268629	-0.090841
C	3.706909	-0.571080	-1.200094	O	-2.644495	1.222607	3.617478
H	3.323472	-1.394184	-1.824528	O	-4.480600	0.068775	1.696008
H	4.785534	-0.489522	-1.397279	O	-2.461504	-0.147455	-0.116534
H	3.569076	-0.859319	-0.148571	O	-5.503008	4.650700	3.849388
C	1.427656	0.532844	-1.344575	O	-1.579608	5.380992	3.856221
H	1.035596	-0.182239	-2.083893	O	-3.079530	3.231408	4.547680
H	1.196424	0.134851	-0.346504	O	-4.015734	6.811597	3.174592
H	0.893443	1.482651	-1.473742	O	-4.599318	4.865533	1.132755
C	5.996669	3.438884	-0.098742	C	-0.382613	5.452710	3.489134
H	7.095460	3.367874	-0.102999	O	-4.879903	4.291710	-1.290930
H	5.725241	4.389236	-0.587278	O	-3.395034	4.384640	-3.515253
H	5.656494	3.481331	0.946236	O	-4.961965	2.063613	-2.779952
H	5.718525	1.313786	-0.361661	O	-1.093554	3.150868	-2.883793
H	3.756701	6.811799	-3.770662	O	-2.644996	0.779840	-2.165469
C	3.905239	7.638152	-3.057223	O	-5.979117	6.646973	-0.652264
C	4.055535	7.116512	-1.631125	O	-3.833928	6.390182	-2.581520
H	4.798687	8.199875	-3.367881	O	-2.103467	7.463862	-0.804280
H	3.038331	8.313404	-3.143232	O	-4.240400	7.719404	1.123173
C	2.830043	6.444686	-1.049028	C	0.029839	3.592099	-2.616455
C	2.905366	5.652152	0.111918	H	-0.819196	1.746222	-0.650308
H	3.881385	5.424933	0.550622	C	-0.861597	7.312008	-0.760040
H	2.046183	5.590729	0.785823	H	-1.924151	6.700026	1.660645
H	1.875817	6.945954	-1.227915	O	-6.370250	1.176977	1.164224
H	2.477710	5.490436	-2.473663	O	-6.945184	3.748813	2.370366
H	4.920961	6.436463	-1.550885	O	-7.204965	4.842901	-0.084490
H	4.277175	7.958059	-0.948926	O	-6.636775	2.273717	-1.286272
H	4.678458	-1.319072	3.345484	H	-4.966141	2.232983	3.062536

C	-2.750225	2.016092	4.591105	H	0.752308	3.698514	-3.455328
C	-5.726790	0.153316	1.511765	H	-0.246516	8.151073	-1.152826
C	-2.500120	-0.192340	-1.373681	H	-2.541022	1.596605	5.597534
C	-6.652090	4.304260	3.462170	H	-6.315806	-0.770903	1.686342
C	-4.298012	7.750152	2.381677	H	-2.377761	-1.192960	-1.839352
H	0.354728	5.791991	4.246652	H	-7.490725	4.498672	4.162417
H	-5.502697	4.488186	-2.003889	H	-4.618277	8.708284	2.841075
C	-3.614664	5.626498	-3.557607	H	-3.624995	6.091991	-4.565447
C	-6.145902	1.888697	-2.379149	H	-6.827024	1.348471	-3.068990
C	-7.066041	6.041039	-0.446638	H	-7.996726	6.621647	-0.614384

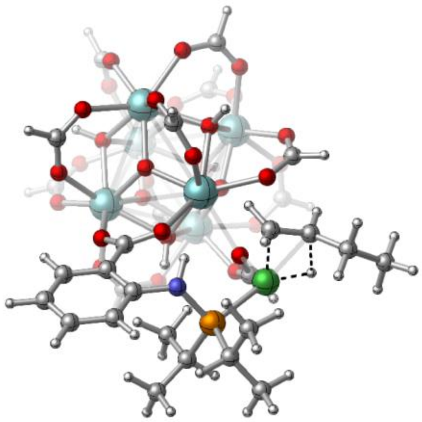

Zero-point correction= 0.774864 (Hartree/Particle)
Thermal correction to Energy= 0.852815
Thermal correction to Enthalpy= 0.853759
Thermal correction to Gibbs Free Energy= 0.670656
Sum of electronic and zero-point Energies= -5682.419890
Sum of electronic and thermal Energies= -5682.341940
Sum of electronic and thermal Enthalpies= -5682.340996
Sum of electronic and thermal Free Energies= -5682.524098

^{1}F-UiO66 (*i*-Pr)
$E(scf)_{gas}$ = -5683.23012855 a.u.
$E(scf)_{CPCM(heptane)}$ = -5683.27678147 a.u.
ν_{min} = 22.25 cm^{-1}

Ni	2.804062	4.131402	-0.583470	O	-3.753975	3.999993	3.648211
P	3.745890	2.203991	-0.857754	O	-0.032872	3.725109	2.253604
N	2.726998	1.084652	-0.058081	O	-1.707227	1.963918	3.379729
H	1.811861	1.527233	-0.044367	O	-2.065286	5.784605	2.473228
C	2.578516	-0.311821	-0.107087	O	-3.876556	4.266707	0.801069
C	1.273908	-0.877497	-0.214624	C	0.975890	3.571317	1.555229
C	3.672797	-1.178033	0.052439	O	-5.168264	4.025634	-1.334932
C	1.111699	-2.272105	-0.122556	O	-4.635306	3.999687	-3.954299
C	3.491569	-2.559267	0.093838	O	-6.382136	2.059509	-2.698760
H	4.673607	-0.761314	0.164714	O	-2.728181	2.100721	-4.222320
C	2.208708	-3.116313	0.013463	O	-4.462759	0.166212	-2.954942
H	0.098706	-2.674761	-0.178528	O	-5.283273	6.499801	-0.304462
H	2.070074	-4.197786	0.064160	O	-4.153350	5.938424	-2.907043
H	4.541552	7.676329	2.280638	O	-1.655890	6.350779	-1.950051
C	3.476079	7.488829	2.488429	O	-2.785014	6.929013	0.666842
C	3.000371	6.183182	1.831391	C	-1.481944	2.219330	-4.323274
H	3.348138	7.438609	3.580096	H	-1.959619	0.532024	-2.269194
H	2.901855	8.350610	2.114573	C	-0.562755	5.913067	-2.377730
C	3.189454	6.221245	0.341013	H	-0.803400	5.335274	0.284552
C	2.179278	6.272858	-0.572355	O	-6.408770	1.120719	1.484597
H	1.138758	6.282365	-0.240198	O	-5.817036	3.615058	2.828027
H	2.364068	6.512154	-1.623199	O	-6.652174	4.994545	0.666051
H	4.217801	6.387405	-0.000385	O	-7.266033	2.500254	-0.673214
H	4.017367	4.422733	-1.296282	H	-4.185061	1.608761	2.711604
H	3.576366	5.339365	2.251948	C	-1.700655	0.707161	3.277476
H	1.940810	6.005928	2.077243	C	-5.967201	-0.055858	1.563964
H	4.362683	-3.208350	0.212307	C	-4.265468	-0.886250	-2.291372
C	0.059869	-0.040726	-0.427500	C	-5.013730	3.975016	3.726800
O	0.234369	1.126859	-0.939264	C	-2.368894	6.840105	1.852087
O	-1.070988	-0.496945	-0.119454	H	1.949933	3.456874	2.073883
Zr	-0.942613	2.942911	-1.224599	H	-5.934073	4.440406	-1.754898
Zr	-3.042658	0.532980	0.336461	C	-4.514049	5.251950	-3.902173
Zr	-2.318603	3.613700	2.002212	C	-7.335080	2.132630	-1.875758
O	1.021083	3.541787	0.268789	C	-6.341402	6.154844	0.291564
Zr	-4.186826	2.335969	-2.534534	H	-1.050251	2.095221	-5.338745
Zr	-3.425755	5.432410	-0.868317	H	0.042863	6.604466	-3.001230
O	-2.735734	3.704025	-1.841630	H	-1.260322	0.143873	4.126883
O	-0.661492	2.452140	-3.390248	H	-6.668832	-0.825893	1.946512
O	-2.335323	1.232829	-1.719610	H	-4.588150	-1.837848	-2.762707
O	-1.789559	2.221901	0.535342	H	-5.455658	4.293380	4.693656
O	-0.056527	4.772463	-2.157591	H	-2.239437	7.794143	2.405163
O	-1.474449	4.659555	0.131729	H	-4.761186	5.814136	-4.826717
Zr	-5.583051	3.032014	0.684815	H	-8.340272	1.855282	-2.255035
O	-3.911684	1.997824	1.869792	H	-7.078034	6.959397	0.495355
O	-4.487853	1.743498	-0.554128	C	5.414262	2.054991	-0.061833
O	-2.148289	0.022244	2.320020	C	3.909690	1.768149	-2.654318
O	-4.806012	-0.449920	1.267651	H	5.704394	0.997429	-0.176018
O	-3.727222	-0.974804	-1.154504	H	4.555739	2.579481	-3.033868

C	6.460768	2.928048	-0.762886
H	7.430827	2.825549	-0.251765
H	6.610769	2.645936	-1.815741
H	6.175015	3.991459	-0.729966
C	5.290604	2.382279	1.431419
H	4.539642	1.750177	1.928269
H	6.257214	2.231622	1.936619
H	5.000165	3.437513	1.570601
C	4.572221	0.416781	-2.943064
H	3.946907	-0.420329	-2.600681
H	4.712489	0.303543	-4.030066
H	5.562996	0.320063	-2.473134
C	2.528472	1.901684	-3.309717
H	2.086741	2.896807	-3.139961
H	2.606626	1.753152	-4.397868
H	1.820017	1.160859	-2.912566

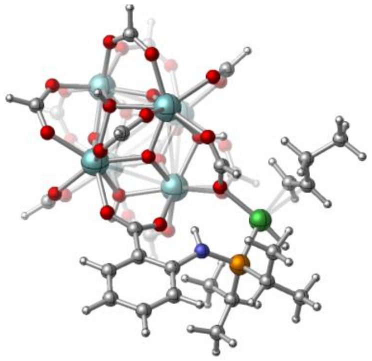

Zero-point correction= 0.777303 (Hartree/Particle)
Thermal correction to Energy= 0.855587
Thermal correction to Enthalpy= 0.856532
Thermal correction to Gibbs Free Energy= 0.672940
Sum of electronic and zero-point Energies= -5682.452826
Sum of electronic and thermal Energies= -5682.374541
Sum of electronic and thermal Enthalpies= -5682.373597
Sum of electronic and thermal Free Energies= -5682.557189

[3]F-UiO66 (*i*-Pr)

$E(scf)_{gas}$ = -5683.19645583 a.u.

$E(scf)_{CPCM(heptane)}$ = -5683.24394704 a.u.

ν_{min} = 20.18 cm^{-1}

Ni	2.222509	4.336317	-1.156333
P	3.676659	2.380982	-0.853100
N	2.673127	1.245333	-0.046961
H	1.756156	1.675713	0.006231
C	2.565429	-0.153176	-0.053923
C	1.278922	-0.776865	-0.082890
C	3.699664	-0.974523	0.068567
C	1.179144	-2.171949	0.070872
C	3.575765	-2.358861	0.172802
H	4.688712	-0.517353	0.100992
C	2.314031	-2.967505	0.187228
H	0.181619	-2.615548	0.072715
H	2.221919	-4.050452	0.287468
H	5.670151	6.562431	1.689731
C	4.761373	6.643756	2.307831
C	3.634196	5.760766	1.753487
H	5.022012	6.339877	3.332663
H	4.462598	7.703036	2.330855
C	3.272323	6.139845	0.348003
C	2.042008	6.503001	-0.072125
H	1.193372	6.518430	0.620580
H	1.884858	6.916501	-1.070309
H	4.101131	6.205651	-0.367300
H	2.999795	4.919631	-2.366721
H	3.970994	4.711229	1.761730
H	2.745859	5.819723	2.403392
H	4.477610	-2.969300	0.263995
C	0.026967	-0.006017	-0.315260
O	0.159325	1.134955	-0.893280
O	-1.091370	-0.476554	0.022293
Zr	-1.032366	2.917235	-1.156443
Zr	-3.122176	0.520714	0.382024
Zr	-2.485933	3.660987	1.998698
O	0.786183	3.502749	0.258510
Zr	-4.213049	2.258242	-2.560602
Zr	-3.519111	5.398348	-0.946185
O	-2.798687	3.662244	-1.859822
O	-0.682304	2.503527	-3.323713
O	-2.377369	1.188621	-1.677570
O	-1.908448	2.229617	0.582781
O	0.063028	4.763268	-1.938800
O	-1.621685	4.674280	0.129833
Zr	-5.701766	3.007955	0.600077
O	-4.054370	2.020549	1.857277
O	-4.555358	1.705679	-0.575941
O	-2.285105	0.080516	2.406009
O	-4.902459	-0.451711	1.281031

O	-3.745413	-1.019076	-1.096538
O	-3.967906	4.065725	3.590507
O	-0.186071	3.826069	2.264776
O	-1.874094	2.056428	3.414937
O	-2.281480	5.853753	2.415410
O	-4.017150	4.267309	0.734785
C	0.799857	3.667645	1.532620
O	-5.240173	3.966865	-1.430781
O	-4.624058	3.905263	-4.026589
O	-6.396139	1.961316	-2.785569
O	-2.702019	2.014261	-4.198988
O	-4.457747	0.074986	-2.935951
O	-5.400283	6.455951	-0.453776
O	-4.159025	5.862819	-3.005756
O	-1.621960	6.260739	-2.033167
O	-2.922604	6.946503	0.547414
C	-1.463508	2.208491	-4.273450
H	-1.972445	0.486613	-2.204656
C	-0.466943	5.879516	-2.288977
H	-0.952019	5.351258	0.282389
O	-6.528006	1.104468	1.417920
O	-6.002691	3.637625	2.722819
O	-6.787500	4.960691	0.506714
O	-7.339372	2.429525	-0.793827
H	-4.348861	1.647265	2.699216
C	-1.855237	0.796262	3.348434
C	-6.076300	-0.064788	1.535833
C	-4.257129	-0.961171	-2.248416
C	-5.229738	4.027403	3.635074
C	-2.556716	6.890825	1.753337
H	1.804106	3.675969	2.000675
H	-5.998410	4.363697	-1.880972
C	-4.491998	5.155509	-3.999185
C	-7.372886	2.040065	-1.990701
C	-6.473487	6.115302	0.118589
H	-1.000646	2.101014	-5.276769
H	0.197941	6.563889	-2.855708
H	-1.420970	0.262301	4.219305
H	-6.779552	-0.834772	1.915135
H	-4.550372	-1.927354	-2.708872
H	-5.701241	4.362202	4.582012
H	-2.449965	7.860629	2.282977
H	-4.699875	5.701113	-4.942874
H	-8.364404	1.746757	-2.392806
H	-7.220297	6.919000	0.284951
C	5.365144	2.294640	-0.067678
C	3.900981	1.853247	-2.632156
H	5.844460	1.390825	-0.478113
H	4.385888	2.745726	-3.067679
C	6.188762	3.515883	-0.502690
H	7.216120	3.436330	-0.114172
H	6.254377	3.611489	-1.597612
H	5.754948	4.449108	-0.110378
C	5.290475	2.143659	1.455304
H	4.727915	1.248880	1.756767
H	6.308273	2.065293	1.869456
H	4.811477	3.012739	1.931646
C	4.774400	0.622115	-2.896809
H	4.317070	-0.293401	-2.494285
H	4.888245	0.484959	-3.984378
H	5.787593	0.716925	-2.478215
C	2.510737	1.712870	-3.267148
H	1.909137	2.632254	-3.177463
H	2.606913	1.505501	-4.344414
H	1.934314	0.896250	-2.808396
